# Genome-Wide DNA Copy Number Analysis of Acute Lymphoblastic Leukemia Identifies New Genetic Markers Associated with Clinical Outcome

**DOI:** 10.1371/journal.pone.0148972

**Published:** 2016-02-12

**Authors:** Maribel Forero-Castro, Cristina Robledo, Rocío Benito, María Abáigar, Ana África Martín, Maryam Arefi, José Luis Fuster, Natalia de las Heras, Juan N. Rodríguez, Jonathan Quintero, Susana Riesco, Lourdes Hermosín, Ignacio de la Fuente, Isabel Recio, Jordi Ribera, Jorge Labrador, José M. Alonso, Carmen Olivier, Magdalena Sierra, Marta Megido, Luis A. Corchete-Sánchez, Juana Ciudad Pizarro, Juan Luis García, José M. Ribera, Jesús M. Hernández-Rivas

**Affiliations:** 1 IBSAL, IBMCC, University of Salamanca, CSIC, Cancer Research Center, Salamanca, Spain; 2 School of Biological Sciences (GEBIMOL), Pedagogical and Technological University of Colombia (UPTC), Tunja, Colombia; 3 Department of Hematology, University Hospital of Salamanca, Salamanca, Spain; 4 Department of Hematology, Clinical University Hospital of Valladolid, Valladolid, Spain; 5 Department of Pediatric Oncohematology, Clinical University Hospital Virgen de la Arrixaca, Murcia, Spain; 6 Department of Hematology, Virgen Blanca Hospital, León, Spain; 7 Department of Hematology, Juan Ramón Jiménez Hospital, Huelva, Spain; 8 Department of Hematology, Miguel Servet Hospital, Zaragoza, Spain; 9 Department of Pediatric Oncohematology, University Hospital of Salamanca, Salamanca, Spain; 10 Department of Hematology, Jerez Hospital, Jerez de la Frontera, Cádiz, Spain; 11 Department of Hematology, Río Hortega Hospital, Valladolid, Spain; 12 Department of Hematology, Nuestra Señora de Sonsoles Hospital, Avila, Spain; 13 Department of Hematology, ICO-Hospital Germans Trias i Pujol, Josep Carreras Research Institute, Badalona, Spain; 14 Department of Hematology, University Hospital of Burgos, Burgos, Spain; 15 Department of Hematology, Rio Carrión Hospital, Palencia, Spain; 16 Department of Hematology, General Hospital of Segovia, Segovia, Spain; 17 Department of Hematology, Virgen de la Concha Hospital, Zamora, Spain; 18 Department of Hematology, Bierzo Hospital, León/Ponferrada, Spain; 19 Cytometry Service (NUCLEUS Research Support Platform), University of Salamanca (USAL), Salamanca, Spain; 20 Institute of Health Science Studies of Castile and León (IESCYL), Salamanca, Spain; University of Sydney, AUSTRALIA

## Abstract

Identifying additional genetic alterations associated with poor prognosis in acute lymphoblastic leukemia (ALL) is still a challenge. Aims: To characterize the presence of additional DNA copy number alterations (CNAs) in children and adults with ALL by whole-genome oligonucleotide array (aCGH) analysis, and to identify their associations with clinical features and outcome. Array-CGH was carried out in 265 newly diagnosed ALLs (142 children and 123 adults). The NimbleGen CGH 12x135K array (Roche) was used to analyze genetic gains and losses. CNAs were analyzed with GISTIC and aCGHweb software. Clinical and biological variables were analyzed. Three of the patients showed chromothripsis (cth6, cth14q and cth15q). CNAs were associated with age, phenotype, genetic subtype and overall survival (OS). In the whole cohort of children, the losses on 14q32.33 (p = 0.019) and 15q13.2 (p = 0.04) were related to shorter OS. In the group of children without good- or poor-risk cytogenetics, the gain on 1p36.11 was a prognostic marker independently associated with shorter OS. In adults, the gains on 19q13.2 (p = 0.001) and Xp21.1 (p = 0.029), and the loss of 17p (p = 0.014) were independent markers of poor prognosis with respect to OS. In summary, CNAs are frequent in ALL and are associated with clinical parameters and survival. Genome-wide DNA copy number analysis allows the identification of genetic markers that predict clinical outcome, suggesting that detection of these genetic lesions will be useful in the management of patients newly diagnosed with ALL.

## Introduction

Acute lymphoblastic leukemia (ALL) is a malignancy of lymphoid progenitor cells characterized by marked heterogeneity at the molecular and clinical levels [1–3]. The WHO classification divides ALL with respect to the presence of genetic abnormalities and it is well known that many of them are strong independent predictive factors of outcome [4]. Most adult patients are considered to be high-risk with a disease-free survival rate of <40% at five years. In contrast, the cure rates are over 85% in pediatric ALLs [3, 5]. However, nearly one quarter of children with ALL display high-risk clinical features, and those with treatment failure or relapse have a worse outcome. Several clinical and genetic factors are routinely used to stratify patients into different risk groups, including white blood cell count, age, minimal residual disease and genetic features. However, resistant disease is not restricted to the high-risk group, and ignorance of the molecular events responsible for leukemogenesis may contribute to therapy failure [6]. Therefore, one of the challenges in the study of ALL is to identify hidden genomic lesions that contribute to the classification, prognosis and choice of risk-adapted treatment [6].

Genome-wide analysis has shown that most patients with ALL have acquired somatically cooperating oncogenic lesions in the leukemic bone marrow cells, which target critical cellular pathways that contribute to leukemogenesis. These include alterations of genes regulating lymphoid development, tumor suppressors, apoptosis regulators, and oncogenes [6–9].

Microarray platforms allow the construction of high-resolution maps of genome-wide copy number alterations (CNAs), which are a hallmark of cancer and are important for understanding the mechanisms of disease and for identifying clinical biomarkers, for example, recurrence risk and response to therapy [10–13]. The oligonucleotide arrays use short nucleotide probes, generally ranging from 20 to 100 nucleotides that are either spotted or synthesized on microarrays. This approach enables very high-resolution genome analysis [9, 13]. Recent studies using microarray methods have shown that a better understanding of genetic changes in leukemic cells is fundamental for a more accurate classification of ALL subtypes [14]. Nevertheless, about 30% of pediatric and 50% of adult ALL patients still lack defined genetic hallmarks of biological and clinical significance [15, 16]. Therefore, the aim of the present study was to characterize the presence of additional copy number changes through a whole-genome oligonucleotide aCGH analysis in children and adults with ALL, and to identify associations of these alterations with biological and clinical features, and with outcome. The present study demonstrates that genome-wide DNA copy number analysis allows the identification of genetic markers that predict clinical outcome in children and adult patients with ALL.

## Materials and Methods

### Patients and data collection

265 ALL patients referred from 19 Spanish centers to the Hematology Service of the University Hospital of Salamanca, Spain, between February 1996 and February 2014 were eligible for this study. All patients were of white ethnicity. The patients were treated in accordance with the risk-adapted protocols of PETHEMA (Programa Español de Tratamientos en Hematología) and SEHOP (Sociedad Española de Hematología y Oncología Pediátrica). Of the 265 ALL patients, 215 were diagnosed as B-lineage ALL (B-ALL), and 50 as T-lineage ALL (T-ALL). Of the 215 B-ALL patients, 115 (53.5%) were children (<18 years) and 100 (46.5%) were adults (≥18 years). Of the 50 T-ALL patients, 27 (54.0%) and 23 (46.0%) were pediatric and adult patients, respectively.

The diagnosis of ALL was based on morphological, immunophenotypic and genetic features of leukemic blast cells, as described previously [17]. The phenotypic diagnosis was performed by the Flow Cytometry Service of the University of Salamanca, based on the most normalized panels and technical protocols employed at the time, using total peripheral blood or bone marrow samples and staining-lyse-wash protocols. Between 1996 and 2002 the phenotypic diagnosis for the ALL included an acute leukemia orientation cell line screening test plus five triple-labelings in B-ALL (TdT/CD10/CD19, CD10/CD20/CD19, CD34/CD38/CD19, CD34/CD22/CD19 and CD19/CD34/CD45) [18] and another five triple-stainings for T-ALL (CD7/CD5/CD3, CD7/CD4/CD8, CD7/CD2/CD3, CD7/CD38/CD34 and TdT/CD7/surface or cytoplasmic (cy)CD3 antibodies conjugated with FITC/PE/PECy5 or PerCP, respectively) [19]. Between 2003 and 2009 a similar strategy was used that included four labelings: CD19/CyCD79a/CD45/CD34, nTdt/CyMPO/CD45/CD34 and CyCD3/CD7/CD45/CD34 like orientation “tube” and the following combinations to classify the leukemia: CD10/CD20/CD19/CD34, CD34/CD38/CD19/CD45, CD15/7.1/CD19/CD5, CyIgM/CD22/HLADR/CD19, CD65/CD13/CD19 and CD2/CD33/CD19 for B-ALL; and CD2/CD8/CD4/CD3, nTdt/CD7/CD3/CD5, TCRab/CD7/CD2/CD38, TCRgd/CD7/CD2/CD38, CD7/CD1a/HLADR/CD34, CD15/CD13/CD7/CD38, CD65/CD7/CD45/CD33, CD10/CD7/CD3/CD56 in T-ALL cases. Since 2009 the phenotypic studies have been performed by Euroflow panels [20], which are designed with five and four eight-color combinations in B and T-ALL, respectively.

Conventional cytogenetic analyses using the G-banding method were done as part of the routine work-up. Fluorescence in situ hybridization (FISH) studies were carried out on the same fixed cells from bone marrow (BM) and/or peripheral blood samples collected at the time of diagnosis. Genetic subtypes and cytogenetic risk groups were established on the basis of karyotype and/or positive FISH results from each patient.

Demographic information, clinical characteristics, risk classification, frontline therapy protocol used, response to therapy and survival were recorded. Risk groups were established according to frontline risk-adapted protocols from the SEHOP and PETHEMA groups. The presence of risk factors such as age group, genetic subtype, white blood cell count (WBC), lactate dehydrogenase (LDH) level, Eastern Cooperative Oncology Group (ECOG) performance status, minimal residual disease (MRD) levels measured during remission induction therapy and outcome were recorded. MRD levels in bone marrow at the end of induction therapy were estimated by flow cytometry [21].

The study was approved by the local ethical committee, the Comité Ético de Investigación Clínica, at the Hospital Universitario de Salamanca. Written informed consent was obtained from each patient or their legal guardian before entering the study.

### DNA isolation and oligonucleotide array comparative genomic hybridization

aCGH was performed on 265 samples of ALL at diagnosis. The genomic DNA was extracted from frozen bone marrow or fixed peripheral blood cell samples with a QIAmp DNA Mini Kit (Qiagen, Valencia, CA, USA) following the manufacturer’s instructions. All samples were tested on an aCGH 12X135K array platform (Roche NimbleGen, Madison, WI, USA). Gender-matched human DNA was used as a reference (Promega, Madison, WI, USA). After labeling, slide preparation, hybridization, scanning and image analysis were performed according to the instructions in the NimbleGen Array User Guide. Segmentation analysis was performed using the CGHweb tool [22]. Significant regions in common between cases were assessed by Genomic Identification of Significant Targets in Cancer (GISTIC) analysis [23]. The statistical significance of the aberrations was displayed as the FDR (false-discovery rate) q values obtained for each region. The method accounts for multiple-hypothesis testing using the FDR framework and assigns a q value to each result, reflecting the probability that the event is due to chance [23]. Values of q<0.05 were considered represent significant amplification and deletion peaks in children and adult patients. The Database of Genomic Variants from Toronto (DGV, http://dgv.tcag.ca/dgv/app/home) was used to exclude DNA variations located in regions with defined copy number variations. Thus, all copy number changes with more than 50% overlap with respect to those reported in DGV were excluded.

### Statistical methods

Continuous variables were summarized as their median and range; categorical variables were described as the frequency and percentage of subjects in each category. Differences between groups were compared by the Chi-square or Fisher’s exact test (for categorical variables), and Student’s independent samples t and Mann—Whitney U tests (for continuous variables), as appropriate. The Chi-square test or Fisher’s exact test were used to identify significant associations between CNA and prognostic factors in ALL. Gains and losses were handled separately. All tests were two-sided and values of p<0.05 were considered to be statistically significant.

The Kaplan—Meier method (log-rank test) was used to assess the relationship between CNAs with respect to overall survival (OS) and event-free survival (EFS) (with a cutoff of <0.05). In accordance with the PETHEMA group, OS was defined as the time from diagnosis to death or last follow-up, and the EFS was defined as the time from diagnosis to first event, including death during induction therapy, failure to achieve remission, relapse at any site, death during remission, or the development of a second malignant neoplasm. Observations on patients without events were censored at the date of the last consultation [24, 25]. Clinical and biological variables associated with worse prognosis in ALL and the most recurrent CNAs were considered in univariate analyses of OS. Cytogenetic risk and risk groups were established according to cytogenetic studies and frontline risk-adapted protocols, respectively. A Cox proportional hazards regression model was used to estimate the hazard ratios (HRs) and 95% confidence intervals (CIs) of risk factors in multivariate analysis to determine which variables were independently associated with OS. Analyses were done using SPSS, version 21.0 (IBM).

The materials, procedures and statistical analyses are described in greater detail in the Supplementary Patients and Methods (S1 File).

## Results

### Patient characteristics

Table 1 shows the characteristics of the patients with ALL included in this study. The median age was 16 years (range 0–84 years): 5 years (range 0–17 years) for the childhood patients (<18 years) and 42 years (range 18–84 years) for the adult patients (≥18 years). The mean percentage of blast counts in bone marrow was 85% (range 34–98%). 42.3% of the whole cohort of patients had a normal karyotype. The median follow-up for survivors of the entire series was 54 months (range 2–189 months); children had a higher 5-year OS rate than adult patients (85.7% *vs*. 41.3%, p<0.0001). Sex, age, phenotype, frontline risk-adapted protocols, risk group, outcome, clinical status, genetic subtype, karyotype, FISH and aCGH analysis of each patient are shown in Table A in S1 File.

**Table 1:** Characteristics of patients with ALL included in the study.

	Whole cohort		Children <18 years		Adults ≥18 years		p^9^
Characteristics	N	%	N	%	N	%	
All patients, n (%)	265	100	142	100	123	100	NA
Age at diagnosis (years), median (range)	16	(0–84)	5	(0–17)	42	(18–84)	NA
Immunophenotype							0.948
B-cell lineage (%)	215	81.1	115	81	100	81.3	
T-cell lineage (%)	50	18.9	27	19	23	18.7	
Sex							0.08
Male (%)	142	53.6	69	48.6	73	59.3	
Female (%)	123	46.4	73	51.4	50	40.7	
Bone marrow blast^1^, median (range)	85	(34–98)	85	(34–98)	85	(34–98)	0.478
WBC count (x10^9^/L), median (range)	18	(1–857)	13	(1–857)	25	(1–496)	0.059
Hb count (g/L), median (range)	94	(26–160)	79	(26–154)	105	(39–160)	**<0.0001**
Platelet count (x10^9^/L), median (range)	61	(2–580)	66	(3–556)	55	(2–580)	0.214
Elevated LDH (IU/L) level^2^ (%)	163	87.2	83	83.8	80	90.9	0.149
Down syndrome (%)	5	2	3	2.2	2	1.7	1.0
Cytogenetics							0.085
Normal, n (%)	112	42.3	67	47.2	45	36.6	
Altered, n (%)	141	53.2	69	48.6	72	58.5	
Not evaluable, n (%)	12	4.5	6	4.2	6	4.9	NA
Cytogenetic risk groups^3^							
Good risk^4^ (%)	112	44.3	45	33.1	8	6.8	**<0.0001**
Poor risk^5^ (%)	59	23.3	9	6.6	50	42.7	**<0.0001**
Others, n (%)	82	32.4	82	60.3	59	50.4	**<0.0001**
Risk group^6^							**<0.0001**
Low risk (%)	60	22.7	60	42.6	0	0	
Intermediate risk (%)	63	23.9	47	33.3	16	13	
High risk (%)	141	53.4	34	24.1	107	87	
MRD at the end of induction^7^							0.053
MRD <0.01% (%)	114	63.7	73	69.5	41	55.4	
MRD ≥0.01% (%)	65	36.3	32	30.5	33	44.6	
SCT (%)	69	32.9	28	23	41	46.6	**<0.0001**
SCT performed in first CR (%)	52	78.8	18	72	34	82.9	0.292
Relapse (%)	65	31	23	18.7	42	48.3	**<0.0001**
Very early relapse^8^ (%)	41	65.1	9	40.9	32	78	**0.014**
Patients alive in first CR (%)	124	58.8	93	79.5	31	33	**<0.0001**
Deaths (whole cohort) (%)	94	38.1	23	17.6	71	61.2	**<0.0001**
Median follow-up (range), months	54 (2–189)		66 (2–189)		38 (3–188)		**0.006**
5-yeas OS probability % (95% CI)	65.7 (NR)		85.7 (NR)		41.3 (18, 11.0–24.9)		**<0.0001**

^1^ By flow cytometry

^2^ Normal range: 135–214 IU/L

^3^ Applied to the whole cohort of patients who had successful cytogenetic study results (n = 253)

^4^ Includes patients with t(12;21)/*ETV6-RUNX1* translocation (E/T) and hyperdiploid karyotype (HD). It should be noted that no adult had the E/T translocation

^5^ Includes patients with t(9;22), t(v;11q23) and hypodiploidy

^6^ Risk-group stratification was established according to PETHEMA protocols based on age, WBC and cytogenetic subgroup

^7^ By flow cytometry

^8^ Time of relapse criteria: very early: earlier than 18 months after initial diagnosis and less than 6 months after cessation of frontline treatment, early: more than 18 months after initial diagnosis, but less than 6 months after cessation of frontline treatment; late: more than 6 months after cessation of frontline treatment

^9^ Probabilities are for comparisons between children and adult patients

**Abbreviations:** WBC, white blood cell; LDH, lactate dehydrogenase; B-ALL, B-cell lineage; T-ALL, T lineage, SCT; ECOG, Eastern Cooperative Oncology Group; MRD, minimal residual disease; SCT, Stem cell transplantation; CR, complete remission; OS, overall survival; NR, not reached; NC, not calculated; NA, not applicable. Figures in bold indicate statistically significant results (p<0.05)

### aCGH detects multiple genetic lesions in pediatric and adult ALL patients

Copy number alterations (CNAs) were present in 91.7% of the 265 patients with ALL (children: 95.1% *vs*. adults: 87.8%, p = 0.033). It is of note that although 112 patients were categorized into the group of normal karyotype and/or normal FISH by initial cytogenetic analysis, aCGH analysis revealed 104 of these cases (93%) to have genomic alterations. The median CNAs per patient was eight (range 0–253), losses being more common than gains (median 4 *vs*. 3). The median size of the losses was 2.20 Mb (range 0.10–199.5 Mb), while the median size of the copy number gains was 2.5 Mb (range 0.1–246.5 Mb).

**Fig 1:**
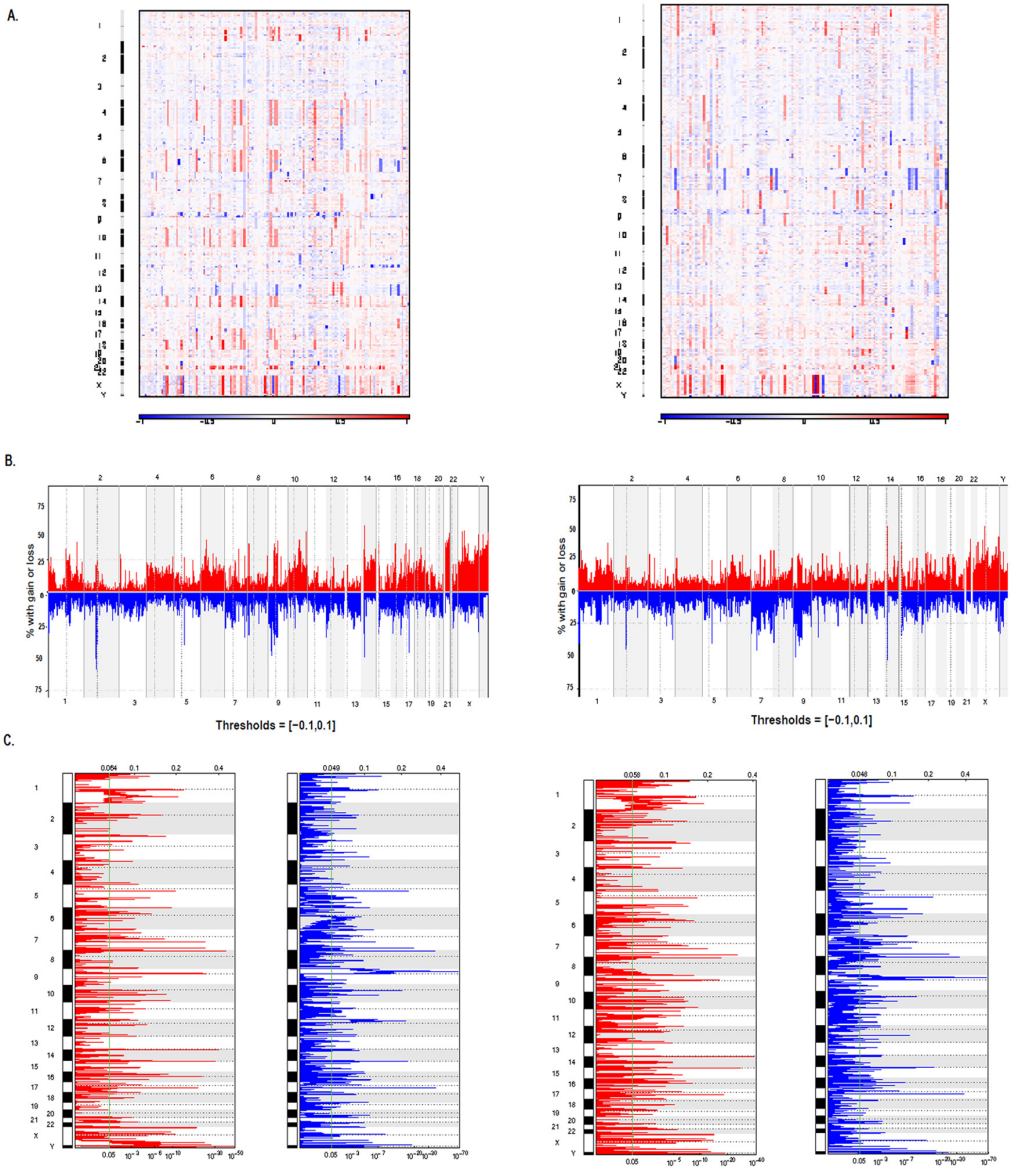
Patterns and frequencies of DNA copy alterations in ALL patients. (A) Log_2_-ratio copy number heatmap of array-based comparative genomic hybridization (aCGH) data in childhood (left) and adult (right) ALL. (gains: red; losses: blue). (B) Overall frequency of copy number changes in childhood (left) and adult (right) ALL. (gains: red; losses: blue) (C) Regions of significant recurrent amplification and deletion in childhood (left) and adult (right) ALL (q<0.05).

The patterns and frequencies of CNAs reproduced the genomic hallmarks typical of a cohort of children and adults with ALL (Fig 1A and 1B). Table B in S1 File shows pairwise comparisons of CNAs according to immunophenotypic, age and cytogenetic subgroups of ALL patients. These CNAs harbored key target genes involved in leukemogenesis, such as *EBF1* (5q33.3), *IKZF1* (7p12.2), *CDKN2A/B* (9p21.3), *ETV6* (12p13.2), *RB1* (13q14.2) and *TP53* (17p13.3-q11.1). Regions of significant recurrent amplification and deletion are shown in Fig 1C. In the whole cohort of children with ALL, there were 66 and 74 significant regions of gain and loss, respectively (Table C in S1 File), whereas in the whole cohort of adults with ALL, there were 81 and 79 regions of gain and loss, respectively (Table D in S1 File).

S1 Fig shows the log_2_-ratio copy number heatmap and regions of significant recurrent amplification and deletion in childhood and adult B-ALL and T-ALL, respectively (q<0.05). In the childhood B-ALL cohort, there were 41 and 49 significant regions of gain and loss, respectively (Table E in S1 File), whereas in the adult B-ALL cohort, there were 55 and 44 regions of gain and loss, respectively (Table F in S1 File). Meanwhile, in the childhood T-ALL cohort, there were 11 significant regions each of gain and loss, respectively (Table G in S1 File), whereas in the adult T-ALL cohort, there were 11 and 5 regions of gain and loss, respectively (Table H in S1 File).

In childhood B-ALL patients, the significant peaks identified by GISTIC included the deletions of *SETD2*/3p21.31; *FYN*, *FOXO3a*, *GRIK2*, *EPHA7*, *BLIMP1*/6q16.3; *KMT2A*(*MLL*)/11q23.3; *ETV6*/12p13.2 and *E2A(TCF3)*/19p13.3 loci, whereas in the adult B-ALL cohort, the significant peaks included the deletions of *MUC4*/3q29; *EBF1*/5q33.3; *RB1*/ 13q14.2 and *CREBBP*/16p13.3 loci. Both groups of B-ALL patients shared recurrent CNAs on *IKZF1*/7p12.2; *CDKN2A*, *CDKN2B*, *IFN*, *MTAP*/ 9p21.3; *BTG1*/12q21.33; *MTA1*/14q32.33, *VPREB1*/22q11.22 and *IL3RA*, *CRLF2*, *CSF2RA*, *CSF2RA*, *P2RY8*, *ASMTL* and *SLC25A6*/ Xp22.33 loci. In both childhood and adult T-ALL patients, the significant peaks identified by GISTIC commonly included the deletions of 9p21.3 (*CDKN2A*, *CDKN2B*, *MTAP* and *DMRTA1* loci).

### Genomic copy number changes are associated with clinical-biological parameters

Table 2 shows the correlation between gains and losses assessed by aCGH and the most relevant clinical and biological characteristics in children and adult patients with ALL. In the pediatric group, we note that patients with gains on 1p36.11 (p = 0.04), 1q32.1 (p = 0.016) or 7p22.3 (p = 0.033) had poor-risk cytogenetics, whereas those with gains on 1q32.1 (p = 0.04) or 15q24.1 (p = 0.025) and losses on 14q32.33 (p = 0.005) had leukocytosis. In the adult group, patients with losses on 3q26.32 (p = 0.019), 7q35 (p = 0.004) or 13q14.2 (p = 0.003) were ≥55 years of age. Finally, adult patients with gains on 19q13.2 (p = 0.04) or losses on 12p13.33 (p = 0.04) had refractory disease or relapse.

**Table 2:** Association of DNA/chromosomal aberrations with clinical characteristics in the whole cohort of patients with ALL.

Age group	Leukocytosis		Poor-risk cytogenetics^1^		Elevated LDH^2^		≥55 years		High-risk group		Resistant ALL^3^	
	Gains	Losses	Gains	Losses	Gains	Losses	Gains	Losses	Gains	Losses	Gains	Losses
Children (n = 142)	1p34.3	14q32.33	1p36.11	None	16p12.1	None	NA	NA	None	None	None	None
	1q32.1		1q32.1									
	15q24.1		7p22.3									
Adults (n = 123)	None	None	19q13.2	None	9q22.1	15q13.2	3p25.1	3q26.32	18q21.1	12q24.21-q24.22	19q13.2	12p13.33
					Xp22.33		5p15.33	7q35		Xq26.3		
							6p21.1	13q14.2				
							6q27					
							20q13.31					

p<0.05 for all correlations. Abbreviations: NA, not applicable; LDH, lactate dehydrogenase.

^1^Includes t(9;22), t(v;11q23) and hypodiploidy;

^2^ Normal range: 135–214 IU/L,

^3^ Includes refractory and/or relapsed disease.

In particular, in childhood B-ALL patients, the gain of 1q32.1 was associated with leukocytosis (p = 0.002), and the deletions of 11q23.3 (p = 0.036), 12q24.33 (p = 0.003), 14q32.22 (p = 0.007) and 20q13.33 (p = 0.036) were associated with MRD positivity (≥0.01%) after induction, whereas, in adult B-ALL patients, the gains of 5p15.33 (p = 0.005), 6p21.1 (p = 0.013), 6q27 (p = 0.014) and 11q25 (p = 0.030) and the loss of 13q14.2 (p = 0.013) were associated with age ≥55 years. In T-ALL patients, the gain of Xq28 was associated with resistant childhood T-ALL (p = 0.014) due to refractory and/or relapsed disease. There were no associations between CNAs and clinical characteristics in adult T-ALL patients.

### Copy number alterations influence the survival of patients with ALL

**Fig 2:**
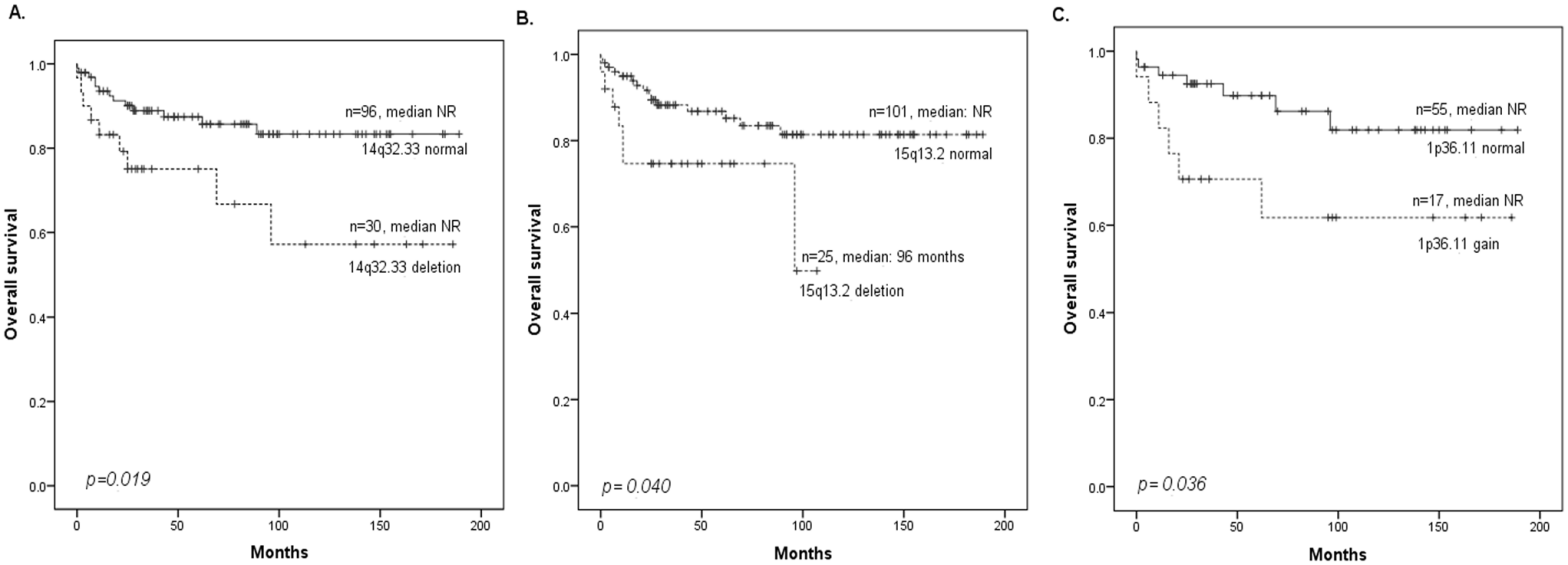
Kaplan—Meier plots demonstrating the effect of copy number changes on overall survival (OS) in children with ALL. (A) OS of the whole cohort of children with ALL with respect to the presence of deletions in 14q32.33. (B) OS of the whole cohort of children with ALL with respect to the presence of deletions in 15q13.2. (C) OS of the children with ALL without chromosomal abnormalities associated with good-risk (hyperdiploid and t(12;21)) or poor risk (t(9;22), t(v;11q23) and hypodiploidy) with respect to the presence of gains of 1p36.11.

Iand J Tables in S1 File summarize the CNAs associated with shorter OS in each group of patients. In the whole cohort of children with ALL (n = 142), lesions associated with shorter OS were losses on 14q32.33 (p = 0.019) or 15q13.2 (p = 0.04). Particularly in the group of children without chromosomal abnormalities associated with good risk (t(12;21) translocation and hyperdiploid) or poor risk (t(9;22), t(v;11q23) and hypodiploidy) (n = 82), the copy number changes associated with shorter OS were the gain on 1p36.11 (p = 0.036) and the losses on 6p25.3 (p = 0.032), 15q13.2 (p = 0.008), 16p13.11 (p = 0.021) or 17p13.1 (p = 0.027) (Table I in S1 File and Fig 2). Although the losses on 1p12 (p = 0.027) and 7p12.2 (p = 0.031) were associated with shorter EFS, there were no associations between CNAs and OS in children with B-ALL (Table K in S1 File). By contrast, the gain on Xq28 was associated with shorter OS in children with T-ALL (p = 0.008). There were no associations between CNAs and EFS in children with T-ALL (Table L in S1 File).

In addition, the present study confirmed in the whole cohort of children with ALL the well known associations of particular clinical and biological variables with worse prognosis, such as the high-risk group (5-year OS, 66% *vs*. 92%, p<0.0001), poor-prognosis frontline therapy due to refractoriness or relapse events (5-year OS, 46% *vs*. 98%, p<0.0001) and age over 10 years (5-year OS, 65% *vs*. 93%, p<0.0001). Multivariate analysis of the group of children without good- or poor-risk cytogenetics showed that the gain on 1p36.11 continued to be a statistically significant prognostic marker (HR 8.2; 95% CI 1.8–37.5, p = 0.006) associated with shorter OS (Table 3).

**Table 3:** Univariate and multivariate analysis of overall survival in children with ALL.

**Whole cohort of children with ALL (n = 142)**							
**Variable**	**Category**	**Univariate**			**Multivariate**		
		HR	95.0% CI	p	HR	95.0% CI	p
Risk group	High risk	4.6	2.0–10.7	**<0.0001**	3.1	1.2–8.2	**0.021**
Age	>10 years	6.4	2.5–16.7	**<0.0001**	3.8	1.3–10.8	**0.014**
14q32.33	Loss	2.7	1.1–6.3	**0.024**	1.6	0.6–4.1	0.361
15q13.2	Loss	2.5	1.0–6.2	**0.047**	2.7	1.0–7.4	0.064
**Children without poor-risk and good-risk cytogenetics**^1^ **(n = 82)**							
**Variable**	**Category**	**Univariate**			**Multivariate**		
		HR	95.0% CI	p	HR	95.0% CI	p
Age	>10 years	9.3	2.0–42.2	**0.004**	10.7	2.1–54.8	**0.004**
1p36.11	Gain	3.0	1.0–9.0	**0.047**	8.2	1.8–37.5	**0.006**
6p25.3	Loss	3.4	1.0–10.9	**0.044**	5.2	0.9–29.3	0.063
15q13.2	Loss	4.0	1.3–12.4	**0.015**	4.9	0.8–31.8	0.095
16p13.11	Loss	3.3	1.1–9.9	**0.031**	0.5	0.1–2.9	0.410
17p13.1	Loss	3.5	1.1–11.3	**0.038**	0.8	0.2–3.8	0.773

^1^Includes 82 children with normal cytogenetics (n = 67) and other abnormalities (n = 15). This group excludes children with good-risk cytogenetics: hyperdiploid and t(12;21) and poor-risk cytogenetics: t(9;22), t(v;11q23) and hypodiploidy.

**Fig 3:**
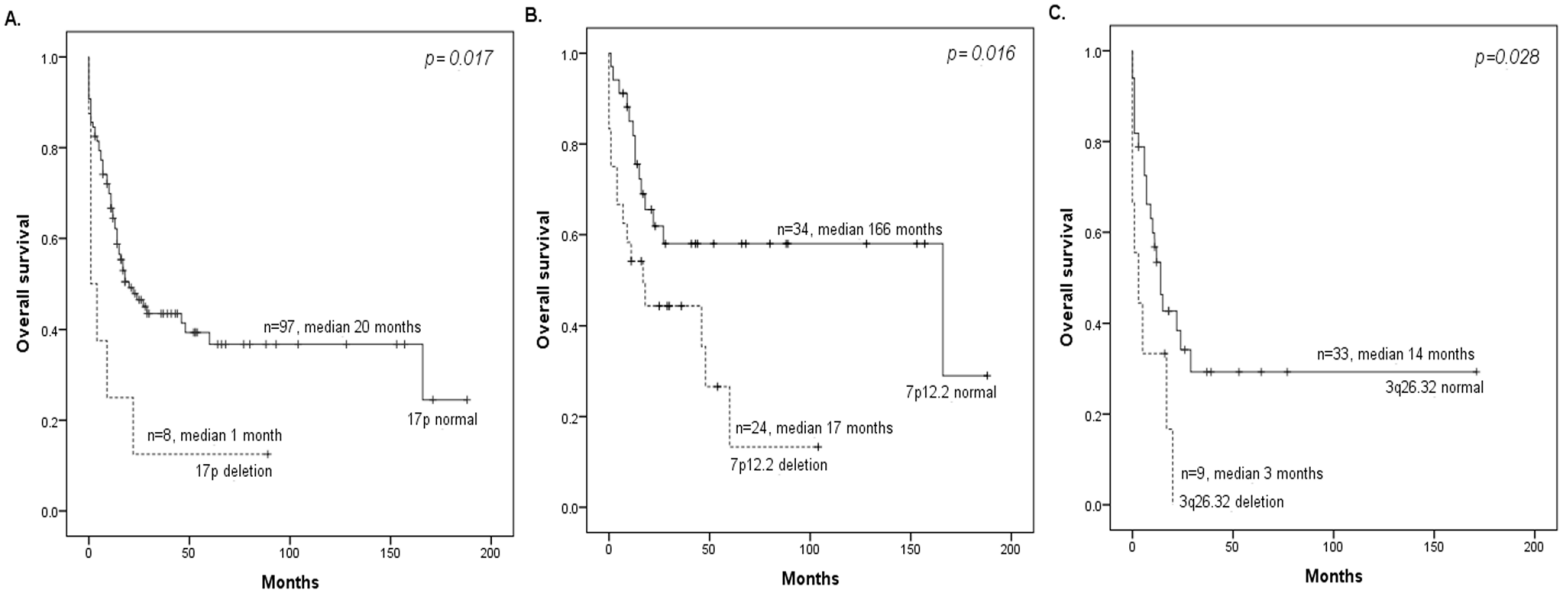
Kaplan—Meier plots demonstrating the effect of copy number changes on overall survival (OS) in adult patients with ALL. (A) OS of the whole cohort of adults with ALL with respect to the presence of deletions in 17p. (B) OS of adults without poor-risk with respect to the presence of deletions in 7p12.2. (C) OS of adults with ALL classified in the poor-risk cytogenetic group with respect to the presence of deletions in 3q26.32. NR, not reached.

The survival analysis of whole cohort of adults with ALL (n = 123) showed that the gains on 2p13.3 (p = 0.033), 6p21.1 (p = 0.013), 11p15.1 (p = 0.035), 19q13.2 (p = 0.011) and Xp21.1 (p = 0.002), as well as the losses on 3q22.3 (p = 0.002), 3q26.32 (p = 0.013), 8q21.13 (p = 0.007), 13q14.2 (p = 0.002) and 17p (p = 0.017) had a negative effect on OS. In particular, in the group of adults without poor-risk cytogenetic abnormalities (n = 67), the gains on 6p21.1 (p = 0.013), 11p15.1 (p = 0.039), 19p13.2 (p = 0.004), 19q13.2 (p = 0.001) and Xp21.1 (p = 0.005), as well as the losses on 3q22.3 (p = 0.04), 3q26.32 (p = 0.023), 7p12.2 (p = 0.016), 13q14.2 (p = 0.001) and 17p (p = 0.021) were associated with shorter OS. Finally, in the group of adults with poor-risk cytogenetics (n = 50), the gains on 5q31 (p = 0.019) and 10p15.3 (p = 0.01), as well as the losses on 1q22 (p = 0.005), 3q22.3 (p = 0.018), 3q26.32 (p = 0.028), 11q23.3 (p = 0.04) and 16q22.1 (p = 0.017) adversely affected the OS (Table J in S1 File and Fig 3).

In adult B-ALL patients the gains on 2p11.2 (p = 0.023), 3p25.3 (p = 0.018), 6p21.1 (p<0.0001), 6q27 (p = 0.027), 10q22.3 (p = 0.007), 11q13.1 (p = 0.027), 18q21 (p<0.0001) and Xq28 (p = 0.024), as well as the losses on 4p14 (p = 0.002), 8q21.13 (p = 0.012), 13q14.2 (p = 0.009) and 15q13.2 (p = 0.003) were associated with shorter OS. Likewise, the gains on 1p36.13 (p = 0.034), 3p25.3 (p = 0.018), 4p16.1 (p = 0.030), 6p21.1(p<0.0001), 10q22.3(p = 0.019), 11q13.1(p = 0.011), 12q13.13 (p = 0.019), 18q21.1(p<0.0001), 21q22.3(p = 0.029), Xq28(p = 0.008), as well as the losses on 1p12(p = 0.034), 4p14 (p = 0.002), 8q21.13 (p = 0.023), 15q13.2 (p = 0.009), 16q22.1 (p = 0.009) were associated with shorter EFS (Table M in S1 File). By contrast, no significant differences between CNAs and shorter OS and EFS were observed in adult T-ALL patients.

The aCGH analysis also confirmed the clinical parameters commonly associated with shorter OS in adults, such as poor prognosis frontline therapy due to refractoriness or relapse events (5-year OS, 21.6% *vs*. 77.5%, p<0.0001), poor-risk cytogenetic abnormalities (5-year OS, 31% *vs*. 50%, p = 0.018) and age ≥55 years (5-year OS, 18% *vs*. 51%, p<0.0001). Multivariate analysis of the whole cohort of ALL adults indicated that the gains on 19q13.2 (HR, 2.8; 95% CI: 1.5–5.3; p = 0.001) and Xp21.1 (HR, 2.2; 95% CI: 1.1–4.4; p = 0.029) and the loss of 17p (HR, 3.3; 95% CI: 1.3–8.7; p = 0.014) were independent markers of poor prognosis in terms of OS. Particularly, in the group of adults without poor risk cytogenetics, the gain on Xp21.1 (HR, 3.7; 95% CI: 1.2–11.5; p = 0.024) and the loss of 17p (HR, 4.0; 95% CI: 1.2–13.7; p = 0.025) were also independent prognostic factors of shorter OS. Finally, the loss on 3q26.32 was the only predictor of OS in the group of adults with poor-risk cytogenetics (HR, 3.2; 95% CI: 1.2–8.3; p = 0.018) (Table 4).

**Table 4:** Univariate and multivariate analysis of overall survival in adults with ALL.

**Whole cohort of adults with ALL (n = 123)**							
**Variable**	**Category**	**Univariate**			**Multivariate**		
		HR	95.0% CI	p	HR	95.0% CI	p
Age	≥55 years	2.7	1.6–4.5	**<0.0001**	3.2	1.7–6.0	**<0.0001**
Cytogenetic risk group	Poor risk	1.8	1.1–3.1	**0.022**	3.0	1.6–5.7	**<0.0001**
2p13.3	Gain	1.8	1.0–3.2	**0.039**	1.5	0.7–3.2	0.281
3q22.3	Loss	2.2	1.3–3.7	**0.003**	1.1	0.5–2.2	0.872
3q26.32	Loss	1.9	1.1–3.1	**0.017**	1.2	0.6–2.5	0.554
6p21.1	Gain	1.9	1.1–3.1	**0.016**	0.8	0.4–1.6	0.460
8q21.13	Loss	2.1	1.0–3.6	**0.010**	1.8	0.9–3.4	0.088
11p15.1	Gain	1.7	1.0–3.0	**0.042**	0.8	0.4–1.6	0.485
13q14.2	Loss	2.3	1.3–4.1	**0.003**	1.5	0.7–3.2	0.268
17p13.3-p11.2	Broad loss	2.5	1.1–5.5	**0.024**	3.3	1.3–8.7	**0.014**
19q13.2	Gain	1.9	1.1–3.1	**0.014**	2.8	1.5–5.3	**0.001**
Xp21.1	Gain	2.2	1.3–3.6	**0.003**	2.2	1.1–4.4	**0.029**
**Adults without poor-risk cytogenetics**^1^ **(n = 67)**							
**Variable**	**Category**	**Univariate**			**Multivariate**		
		HR	95.0% CI	p	HR	95.0% CI	p
Age	≥55 years	2.9	1.4–6.1	**0.004**	2.5	1.0–6.1	**0.042**
3q22.3	Loss	2.1	1.0–4.3	**0.050**	0.9	0.2–3.8	0.890
3q26.32	Loss	2.3	1.1–4.8	**0.029**	0.7	0.2–2.0	0.485
6p21.1	Gain	2.5	1.2–5.4	**0.017**	0.7	0.2–2.5	0.617
7p12.2	Loss	2.4	1.0–5.0	**0.020**	1.3	0.5–3.3	0.618
11p15.1	Gain	2.2	1.0–4.6	**0.046**	0.8	0.3–2.4	0.729
17p13.3-p11.2	Broad loss	2.9	1.1–7.8	**0.029**	4.0	1.2–13.7	**0.025**
13q14.2	Loss	3.3	1.5–7.1	**0.003**	1.5	0.3–6.4	0.587
19p13.2	Gain	2.9	1.3–6.2	**0.007**	1.2	0.4–3.7	0.767
19q13.2	Gain	3.4	1.6–7.5	**0.002**	4.3	0.9–19.4	0.059
Xp21.1	Gain	2.9	1.3–6.5	**0.007**	3.7	1.2–11.5	**0.024**
**Adults with poor-risk cytogenetics**^2^ **(n = 50)**							
**Variable**	**Category**	**Univariate**			**Multivariate**		
		HR	95.0% CI	p	HR	95.0% CI	p
Age	≥55 years	2.2	1.0–4.6	**0.046**	2.2	0.9–5.2	0.089
1q22	Loss	2.8	1.3–6.3	**0.010**	2.2	0.7–6.5	0.170
3q22.3	Loss	2.4	1.1–5.3	**0.026**	1.0	0.4–2.9	0.973
3q26.32	Loss	2.4	1.0–5.6	**0.039**	3.2	1.2–8.3	**0.018**
5q31	Gain	2.4	1.1.5.4	**0.028**	1.9	0.8–4.9	0.172
10p15.3	Gain	2.9	1.2–6.9	**0.017**	2.2	0.8–5.9	0.118
16q22	Loss	2.4	1.1–5.1	**0.024**	2.4	0.9–6.2	0.072

^1^Includes patients without t(9;22), t(v;11q23) and hypodiploidy.

^2^Includes patients with t(9;22), t(v;11q23) and hypodiploidy.

It should be noted that some CNAs were commonly associated with both high-risk clinical characteristics and OS. Thus, in the whole cohort of children with ALL, the loss on 14q32.33 was associated with leukocytosis (p = 0.005) and OS (p = 0.019), although this alteration was not an independent risk factor in the multivariate analysis. Meanwhile, in the whole cohort of adults with ALL, there were more CNAs commonly associated with both high-risk clinical characteristics and OS. Particularly, the gain on 19q13.2 was correlated with poor-risk cytogenetics (p = 0.036) and resistant ALL due to refractory and/or relapse disease (p = 0.044). This alteration was also associated with shorter OS (p = 0.011) in the whole cohort of adults with ALL and was an independent prognostic factor in multivariate analysis (HR, 2.8, 95% CI: 1.5–5.3; p = 0.001). The loss on 3q26.32 was associated with older age (≥55 years) (p = 0.019) and reduced OS (p = 0.028) in adults with poor-risk cytogenetics, and was also of independent prognostic value (HR, 3.2, 95% CI: 1.2–8.3; p = 0.018) in these patients. Others alterations, such as the gain on 6p21.1 (p = 0.008) and the loss on 13q14.2 (p = 0.003) were associated with older age (≥55 years) and OS (p = 0.013 and p = 0.002), respectively, but were not of independent prognostic value in ALL adults.

### Chromothripsis in ALL patients

Chromothripsis (cth) was observed in three of the 265 ALL patients. All three patients were adults (a 57-year-old woman, and two men, aged 25 and 54 years). Cth involved chromosomes 6 (B-ALL), 14q (T-ALL), and 15q (B-ALL), respectively (S2 Fig). There was an absolute, though not statistically significant, difference in the number of CNAs between the patients with and without cth (median of 26 CNAs per case *vs*. median of 7 CNAs per case, p = 0.126). It is of note that all patients had features associated with poor prognosis. The female B-ALL patient with cth(6) had a complex karyotype with isochromosome 9q, which involved losses in *CDKN2A*, *JAK2* and *PAX5* loci, and also had focal deletions of *BTG1* and *RB1* loci. She was treated with a high-risk protocol and received stem cell transplantation during first remission, remaining in complete remission (CR) until her death. The male T-ALL patient with cth(14q) had focal *IKZF1* deletion and was treated with a high-risk protocol, attaining complete remission after induction. He did not experience any event and was alive in CR at last follow-up. Finally, the Down syndrome B-ALL patient had cth(15q) and died during induction. This patient had an altered karyotype and focal deletions in the *IKZF1* and *BTG1* genes.

## Discussion

The risk stratification of ALL is mainly based on genetic analyses. However, the presence of cryptic aberrations may contribute to therapy failures. Therefore, one of the current challenges in the study of ALL is to identify hidden genomic lesions that may be related to patient outcome [3, 6]. High-density resolution aCGH has been used in hematological malignancies to identify submicroscopic copy number gains and losses across the genome [26]. In this study, new genetic imbalances were observed in 91.7% of ALL patients and related to the prognosis of childhood and adult ALL. Most of the patients with normal cytogenetics had CNAs by aCGH, indicating that, in most cases, ALL originates from cooperating genetic lesions, and that consequently the chromosomal classification of ALL is not representative of the whole leukemic clone [5–7, 10, 15, 27–31].

Microarray analysis also revealed specific patterns of genomic lesions related to the immunophenotypic, cytogenetic and age subgroups, suggesting that the clinical heterogeneity observed in ALL could be explained, at least partially, by the presence of secondary chromosomal abnormalities [7, 15, 32–35]. In agreement with previous studies using array-CGH or SNP array methods [7, 10, 15, 27, 28, 30–33, 36–42], many small (<5 Mb) genetic lesions detected in children and adults with ALL harbored biological and clinically relevant ALL-related genes, such as lymphoid transcription factors (*PAX5*, *IKZF1* and *EBF1*), transcriptional regulators and coactivators (*ETV6* and *ERG*), tumor suppressors (*CDKN2A/B*, *RB1* and *TP53*), as well as putative regulators of apoptosis (*BTG1*) (C-H Tables in S1 File). This confirms that several pathways are deregulated in ALL [34, 35].

In this study we have identified visible and cryptic imbalances associated with poor-risk clinical features and survival. In children, the gain on 1q32.1 was associated with leukocytosis and poor-risk cytogenetics. Duplications in 1q have been reported in B-cell precursor ALL and Burkitt lymphoma [43]. This abnormality has been associated with B-cell precursor ALL and seems to promote clonal evolution during the progress of hematological disorders [44]. Previous studies have indicated that, although the effect of the dup(1)(q32q21) on prognosis in ALL has not yet been defined, the early relapse in some cases might indicate the dismal prognosis of this alteration. It is important to note that the long arm of chromosome 1 is associated with high chromosomal instability in hematological neoplasias, probably because of specific chromatin properties of this gene-rich region. Furthermore, gene dosage effects might play a role in the specific effects of gains of 1q [44].

In our study, cryptic deletions of 14q32 were associated with leukocytosis and shorter OS in whole cohort of children. Previous studies have reported that miRNA clusters are deleted in some ALL cases bearing cryptic deletions at 14q32 [45]. The downregulation of miRNA clusters may influence the expression level of target genes that modify critical cellular pathways, such as the B-cell lymphoid differentiation pathway (e.g., *BCL11a* gene, a transcription factor involved in lymphoid differentiation, upstream of the transcription factors *EBF1* and *PAX5*). Thus, the loss of heterozygosity on the 14q32/miRNA cluster may be another mechanism involved in lymphoid B-cell transformation and differentiation, and so could be used as a diagnostic marker and therapeutic target in subsets of ALL [45].

Losses on 15q13 were associated with significantly shorter OS in our whole cohort of children with ALL. This region harbors the leukemia-related gene *TJP1 (ZO-1)*, which encodes a protein involved in signal transduction at cell-cell junctions. Although *TJP1 (ZO-1)* deletions are infrequent in leukemia, previously have detected the hypermethylated status of *TJP1 (ZO-1)* gene promoter region in newly diagnosed acute leukemia and relapse disease patients [46]. This status is closely correlated with the pathogenesis and progression of the disease, so the *TJP1 (ZO-1)* gene has been proposed as being a clinical molecular marker of leukemia [46].

In adult ALL, we observed that the presence of deletions on 13q14 was associated with old age and shorter OS in the whole cohort of adults and those adults without poor-risk cytogenetics [28]. Band 13q14 contains the *RB1* gene. This tumor suppressor is rarely reported to be deleted in T-ALL. In contrast, deletion of *RB1* has been detected in 30% of B-ALL and nearly 60% of B-CLL cases. Thus, the *RB1* pathway is a potential target for therapy of ALL [47]. Furthermore, in our whole cohort of adults, the losses on 17p and gains on 19q13.2 and Xp21.1 were identified as risk factors independently associated with significantly shorter OS, the 17p deletion being the strongest predictor of poor survival in adults with ALL. The tumor suppressor gene *TP53* is affected by 17p deletions and plays a crucial role in cell cycle regulation and apoptosis after DNA damage, and its role in tumorigenesis is well recognized in solid and hematological malignancies [48]. *TP53* abnormalities have been associated with resistance to treatment and worse prognosis of patients in several tumors. In ALL, *TP53* gene abnormalities are important in relapse in childhood and adult ALL, in which they independently predict the high risk of treatment failure in a substantial number of patients [49]. The presence of *TP53* alterations has been associated with a reduced response rate to induction therapy and correlated with shortened survival duration, even after successful reinduction therapy [50].

We also observed that the deletions in the 7p12.2 region were associated with shorter EFS in children with B-ALL and shorter OS in adults not included in the poor cytogenetics risk group. This region harbored the *IKZF1* gene, which encodes a zinc finger transcription factor that is required for the earliest stages of lymphoid lineage commitment, and is expressed in stem cells and multipotent progenitors; the loss of *IKZF1* leads to an arrest at an even earlier stage of lymphoid development [51]. Previous studies in pediatric patients with ALL have showed that the genetic alteration of *IKZF1* is associated with a very poor outcome in children with B-cell—progenitor ALL [51]. Likewise, in Philadelphia-negative adults have also shown that *IKZF1* deletions are associated with a worse 5-year OS in univariate analysis but are not an independent risk factor in multivariate analysis [52, 53]. *IKZF1* deletions are secondary events, so their effect on outcome is likely to depend not only on the therapy delivered, but also on the nature of the primary chromosomal abnormality. As in other studies, it was difficult to assess whether the 7p12.2 deletions contribute to the poor outcomes seen in adults with poor-risk cytogenetics, because the incidence of *IKZF1* deletions is linked to the primary chromosomal abnormality (Ph+), which probably has a greater impact in this group of patients [52]. Larger studies are needed to determine whether deletion of the *IKZF1* gene itself causes the poor outcome seen in these patients or if the effect is driven solely by the primary genetic event [52].

We established that the losses on 3q26.32 were associated with inferior OS in adults with ALL. Particularly, in the group of adults with poor cytogenetics risk, this loss was selected as a risk factor independently associated with shorter OS. The 3q26.32 region harbors the *TBL1XR1* gene, which codes for an F-box-like protein responsible for regulating the nuclear hormone repressor complex stability. *TBL1XR1* is focally deleted in pediatric ALL [32, 33]. However, in our study this loss also significantly affected the adult group. Glucocorticoids (GCs) exert anti-leukemic effects through the induction of apoptosis and/or cell cycle arrest, and are therefore a central component in the treatment of lymphoid malignancies, particularly childhood ALL [54]. Previous studies have indicated that loss of *TBL1XR1* is a driver of glucocorticoid resistance in ALL and that epigenetic therapy may have applications for restoring drug sensitivity at relapse [55].

In addition, high-resolution oligonucleotide aCGH also revealed three of the 265 ALL patients to have chromothripsis. The overall incidence of this abnormality may be around 1% of ALL cases. This submicroscopic genetic abnormality is not detectable with standard cytogenetics or FISH. Chromothripsis is caused by a process whereby localized genomic regions are shattered and rearranged in a one-off catastrophic event [56–66] in which the presence of juxtaposed gains and losses leads to an absolutely abnormal genetic architecture [56–66]. Chromothripsis has been reported in a few specific subgroups of ALL, such as early T-cell precursor ALL (ETP ALL) [67], and sporadic and rob(15;21)c-associated (constitutional Robertsonian translocation between chromosomes 15 and 21) iAMP21 B-ALL [68, 69]. However, no cases with a high-hyperdiploid karyotype showed chromothripsis [70]. Several studies suggest that chromothripsis is associated with an aggressive malignant phenotype and rapid disease progression in the context of the respective tumor type [56]. In this study we reported some cases of this genetic chaos affecting different chromosomes, both in B and T-ALL. In these cases, chromothripsis was associated with multiple structural rearrangements, hidden microdeletions involving the *IKZF1*, *BTG1* and *RB1* genes, and broad deletions in *CDKN2A* and *PAX5* due to the loss of chromosome 9p. However, there was no association between the presence of chromothripsis with an aggressive malignant phenotype and/or rapid disease progression. In fact, two patients were long-term survivors.

In summary, the present study demonstrates that genome-wide DNA copy number analysis allows the identification of genetic markers that predict clinical outcome, suggesting that detection of these genetic lesions will be useful for managing patients newly diagnosed with ALL. The comprehensive analysis of these lesions during diagnosis and treatment will provide new and relevant information about how these lesions may influence the course of the disease and its response to treatment.

## Supporting Information

S1 File Supplementary Patients and Methods. Patient characteristics, clinical status, cytogenetics, and aCGH analysis of the studied patients **(Table A)**. Pairwise comparisons of CNAs according to immunophenotypic, age and cytogenetic subgroups of ALL patients **(Table B)**. Regions of significant recurrent amplification and deletion in the whole cohort of children with ALL (n = 142) (q<0.05) **(Table C)**. Regions of significant recurrent amplification and deletion in the whole cohort of adults with ALL (n = 123) (q<0.05 **(Table D**). Regions of significant recurrent amplification and deletion in the whole cohort of children with B-ALL (n = 115) (q<0.05) **(Table E**). Regions of significant recurrent amplification and deletion in the whole cohort of adults with B-ALL (n = 100) (q<0.05) **(Table F**). Regions of significant recurrent amplification and deletion in the whole cohort of children with T-ALL (n = 27) (q<0.05) **(Table G)**. Regions of significant recurrent amplification and deletion in the whole cohort of adults with T-ALL (n = 23) (q<0.05) **(Table H)**. CNAs associated with shorter OS in the groups of child patients with ALL **(Table I)**. CNAs associated with shorter OS in the groups of adult patients with ALL **(Table J)**. CNAs associated with shorter EFS in children with B-ALL **(Table K)**. CNAs associated with shorter OS in children with T-ALL **(Table L)**. CNAs associated with shorter OS and EFS in adults with B-ALL **(Table M)**.
(DOCX)

S1 Fig Patterns and frequencies of DNA copy alterations in B-ALL and T-ALL patients. (A). Log2-ratio copy number heatmap of array-based comparative genomic hybridization (aCGH) data in child B-ALL (n = 115)/adult B-ALL (n = 100) and child T-ALL (n = 27)/adult T-ALL (n = 23) (gains: red; losses: blue). (B). Regions of significant recurrent amplification and deletion in child B-ALL (n = 115)/adult B-ALL (n = 100) and child T-ALL (n = 27)/adult T-ALL (n = 23) patients (q<0.05).
(TIF)

S2 Fig Chromothripsis patterns in three newly diagnosed ALL patients. As is typical of chromothripsis (cth), copy-number profiles showed multiple oscillations between two DNA copy-number states. (A) B-ALL patient with cth on chromosome 6. (B) T-ALL patient with cth on chromosomes 14. (C) B-ALL patient with cth on chromosome 15.
(TIF)
