# Supplementary material for: Genome-Wide DNA Copy Number Analysis of Acute Lymphoblastic Leukemia Identifies New Genetic Markers Associated with Clinical Outcome
Source: PLoS One. 2016 Feb 12;11(2):e0148972. doi: 10.1371/journal.pone.0148972 (PMC4752220; doi:10.1371/journal.pone.0148972)
Supplement: S1 File — Patient characteristics, clinical status, cytogenetics, and aCGH analysis of the studied patients (Table A). Pairwise comparisons of CNAs according to immunophenotypic, age and cytogenetic subgroups of ALL patients (Table B). Regions of significant recurrent amplification and deletion in the whole cohort of children with ALL (n = 142) (q<0.05) (Table C). Regions of significant recurrent amplification and deletion in the whole cohort of adults with ALL (n = 123) (q<0.05 (Table D). Regions of significant recurrent amplification and deletion in the whole cohort of children with B-ALL (n = 115) (q<0.05) (Table E). Regions of significant recurrent amplification and deletion in the whole cohort of adults with B-ALL (n = 100) (q<0.05) (Table F). Regions of significant recurrent amplification and deletion in the whole cohort of children with T-ALL (n = 27) (q<0.05) (Table G). Regions of significant recurrent amplification and deletion in the whole cohort of adults with T-ALL (n = 23) (q<0.05) (Table H). CNAs associated with shorter OS in the groups of child patients with ALL (Table I). CNAs associated with shorter OS in the groups of adult patients with ALL (Table J). CNAs associated with shorter EFS in children with B-ALL (Table K). CNAs associated with shorter OS in children with T-ALL (Table L). CNAs associated with shorter OS and EFS in adults with B-ALL (Table M). (DOCX) [file pone.0148972.s001.docx]

**Supporting information (S1 File)**

**Genome-Wide DNA Copy Number Analysis of Acute Lymphoblastic Leukemia Identifies New Genetic Markers Associated with Clinical Outcome**

**Table of contents of supplementary patients and methods**

[*Supplementary Patients and Methods……………………………………………………………………..*5](#_Toc420930907)

[DNA isolation and oligonucleotide array comparative genomic hybridizations…………….....5](#_Toc420930908)

[Oligonucleotide array comparative genomic hybridizations……………………………………5](#_Toc420930909)

[Array-CGH data analysis methods………………………………………………………………6](#_Toc420930910)

**List of supplementary tables**

[*Supplementary tables…………………………………………………………………………………..…..* 8](#_Toc420930942)

[Table A. Patient characteristics, clinical status, cytogenetics, and aCGH analysis of the studied patients………………………………………………………](#_Toc420930944)…………………………………..8

[Table B. Pairwise comparisons of CNAs according to immunophenotypic, age and cytogenetic subgroups of ALL patients…………………………………………………………………… 30](#_Toc420930945)

[Table C. Regions of significant recurrent amplification and deletion in the whole cohort of children with ALL (n=142) (q<0.05)…………………………………………………………..32](#_Toc420930946)

[Table D. Regions of significant recurrent amplification and deletion in the whole cohort of adults with ALL (n=123) (q<0.05)……………………………………………………………………34](#_Toc420930947)

[Table E. Regions of significant recurrent amplification and deletion in the whole cohort of children with B-ALL (n=115) (q<0.05)……………………………………………… ….. 37](#_Toc420930948)

[Table F. Regions of significant recurrent amplification and deletion in the whole cohort of adults with B-ALL (n=100) (q<0.05)……………………………………………………………….. 38](#_Toc420930949)

[Table G. Regions of significant recurrent amplification and deletion in the whole cohort of children with T-ALL (n=27) (q<0.05)… …………………………………………………….. 40](#_Toc420930948)

[Table H. Regions of significant recurrent amplification and deletion in the whole cohort of adults with T-ALL (n=23) (q<0.05)……………………………………………………………………..41](#_Toc420930949)

[Table I. CNAs associated with shorter OS in the groups of child patients with ALL…………………………………………………………………………………………….. .41](#_Toc420930948)

[Table J. CNAs associated with shorter OS in the groups of adult patients with ALL……………………………………………………………………………………………... 42](#_Toc420930949)

Table K. CNAs associated with shorter EFS in children with B-ALL………………………….44

Table L. CNAs associated with shorter OS in children with T-ALL……………………………44

Table M. CNAs associated with shorter OS and EFS in adults with B-ALL…………………...44

**Supplementary Information References**

**Supplementary patients and methods**

**DNA isolation and oligonucleotide array comparative genomic hybridizations**

The genomic DNA was extracted from frozen bone marrow or peripheral blood fixed cell samples using QIAamp DNA Mini Kit (Qiagen, Valencia, CA, USA) following the manufacturer’s instructions. DNA quality was assessed by A260/A280 ratio on NanoDrop ND-1000 spectrophotometer (NanoDrop Technologies, Wilmington, DE, USA) and by 1% agarose gel electrophoresis. A260/A280 ≥ 1.8 and A260/A230 ≥ 1.9 ratios were required for optimal labeling of DNA samples.

FISH with LSI *ETV6-RUNX1 (TEL-AML1)*, LSI *BCR/ABL* and LSI *KMT2A* (*MLL*) rearrangement probes was performed in most patients according to standard methods (Vysis, Abbott Park, IL, USA). In particular patients additional locus-specific (LSI) (e.g., LSI *PBX- TCF3 (E2A)*), centromeric (CEP) and whole chromosome painting (WCP; Vysis, Abbott Park, IL, USA) probes were used to clarify the cytogenetic study. Reverse transcriptase–polymerase chain reaction (RT-PCR) assays from leukemic cells were performed to identify *ETV6-RUNX1 (TEL-AML1)*, *BCR-ABL1*, *KMT2A (MLL)-AF4*, and *TCF3 (E2A)-PBX1* fusion genes, as previously described [[1](#_ENREF_1)].

For cytogenetic categorization, pediatric and adult patients were classified into cytogenetic subgroups according to the recurring aneuploidies and chromosomal translocations in ALL: low hyperdiploidy (47-50 chromosomes), high hyperdiploidy (>50 chromosomes), low hypodiploidy (<44 chromosomes), high hypodiploidy (44-45 chromosomes), t(12;21)(p12,q22) *ETV6-RUNX1 (TEL-AML1)* fusion, t(9;22)(q34;q11) *BCR-ABL1* fusion, t(1;19)(q23;p13) *TCF3(E2A)-PBX1* fusion, t(11;v)(q23;v) *KMT2A(MLL)* rearrangement, normal karyotype and/or normal FISH and other miscellaneous abnormalities (pseudo-diploid karyotype, other single or double chromosomal abnormalities, and complex karyotype with three or more abnormalities).

**Oligonucleotide array comparative genomic hybridizations**

In brief, patient DNA and normal control DNA (Human Genomic DNA: Male/Female, Promega, Madison, WI, USA) samples were denatured and labeled in parallel with Cy3 for the test group and Cy5 for the control group, each through a random priming method, using Klenow fragments (NimbleGen Dual-Color DNA Labeling Kit, Roche NimbleGen, Inc., Madison, WI, USA). Following cleanup and quantification, the test and sex-matched reference DNA samples were combined in equimolar amounts and loaded into one of the twelve filled ports on the microarray slide. Hybridization was carried out in a NimbleGen Hybridization Chamber for 16-20 hours at 42°C. Subsequently, the microarray slide was washed, dried and scanned at 2-μm resolution using a NimbleGen MS 200 microarray scanner. To avoid slide batch spotting bias, samples were hybridized in random order.

**Array-CGH data analysis methods**

Array image files (532.tif and 635.tif; 532 = Cy3, 635= Cy5) generated by the MS 200 Data Collection Software were imported into Nexus Copy Number software (version 4.1) (Biodiscovery, Inc., Hawthorne, CA, USA) for analysis. Log_2_ values of the raw data were normalized using the loess algorithm implemented in the R package. Quality control measures consisted of checking the consistency of signal distributions across samples; unsupervised clustering was performed to check outlier samples using SIMFIT statistical software (*www.simfit.org.uk*) (data not shown). The batch effect was corrected with ComBat package in R. Segmentation analysis was performed in each case using the CGHweb R package to compare DNA copy number segmentations derived from eight of the most common algorithms [[2](#_ENREF_2)]. A heatmap panel of the segmented profiles for each method and a consensus profile were generated. Each genomic region exhibiting a copy number change was examined using the University of California at Santa Cruz Genome Browser (*http://genome.cse. ucsc.edu*) tool to determine the location and significance of the change. The Database of Genomic Variants from Toronto (DGV, *http://dgv.tcag.ca/dgv/app/home*) was used to exclude lesions associated with copy number variations; thus, all copy number changes with more than 50% overlap with respect to those reported in DGV were excluded. Sex chromosomes were included from array-CGH data analysis. The CNAs ≥0.5 Mb and detected by at least five consecutive aCGH probes were retained for copy number analysis. Large-scale or broad copy number alterations corresponded to regions larger than 50% of a chromosome arm. Significant regions in common between cases were assessed using Genomic Identification of Significant Targets in Cancer (GISTIC) analysis [[3](#_ENREF_3)]. A value of q<0.05 was used to identify significant amplification and deletion peaks in children and adult patients. All genome-based data reported in this manuscript correspond to NCBI build 36 (hg18- Mar. 2006). The primary aCGH microarray data have been deposited in the NCBI Gene Expression Omnibus (GEO, http://www.ncbi.nlm.nih.gov/geo/) and are accessible through GEO Series accession number GSE75671. The data are also available at <http://www.ncbi.nlm.nih.gov/geo/query/acc.cgi?token=ufczceaqvvudfup&acc=GSE75671>.

**Supplementary tables**

**Table A. Patient characteristics, clinical status, cytogenetics, and aCGH analysis of the studied patients.**

| Patient ID | Sex/Age^1^(years) | Phenotype | Moment evaluated | Risk group | Frontline therapy | Outcome | Clinical status | aCGH findings | | | | Genetic subtype | Karyotype | Positive *FISH* results | Positive molecular biology results |
| --- | --- | --- | --- | --- | --- | --- | --- | --- | --- | --- | --- | --- | --- | --- | --- |
|  |  |  |  |  |  |  |  | aCGH | Gains (≥5Mb) | Losses (≥5Mb) | Cth event |  |  |  |  |
| 1 | F/1 | B-ALL | Diagnosis | IR | PETHEMA LAL-RI/96 | CR | CR-A | A | 1q |  |  | Hyper (>50) | F | TEL gain-15% AML1 gain-15% MLL gain-16% ABL gain-10% BCR gain-10% | UNKN |
| 2 | M/2 | B-ALL | Diagnosis | IR | PETHEMA LAL-RI/96 | CR | CR-A | A | 6 10 14 18 21 X |  |  | Hyper (>50) | 52-54,XY,+5,+10,+15,+20,+21,+22[5]/46,XY[6] | ND | UNKN |
| 3 | F/2 | B-ALL | Diagnosis | IR | LAL/SEHOP-PETHEMA 2013 | CR | CR-A | A | 4 5 6 8 10 14 21 X | 19 20 22 |  | Hyper (>50) | F | ETV6 gain-16% RUNX1 gain 55% | N |
| 4 | F/3 | B-ALL | Diagnosis | IR | PETHEMA LAL-RI/96 | CR | CR-A | A | 10 21 18 |  |  | Hyper (>50) | 51,XX,+21,4mar[4]/46,XX[17] | AML1 gain-94% ABL gain-51% MLL gain-25.5% | UNKN |
| 5 | F/4 | B-ALL | Diagnosis | HR | SEHOP/LAL/AR-SHOP-2005 | CR | CR-A | A | 10 14 21 |  |  | Hyper (>50) | 50-52,XX,+10,+17,+18,+21,+mar[3]/46,XX[8] | AML1 gain-48.5% | UNKN |
| 6 | F/4 | B-ALL | Diagnosis | LR | SEHOP/LAL-SHOP-2005 | CR | CR-A | A | 14 21 |  |  | Hyper (>50) | 50-53,XX,+9,+21,+21[10] | AML1 gain-81% ABL gain-83% | UNKN |
| 7 | F/4 | B-ALL | Diagnosis | LR | UNKN | UNKN | Rel-D | A | 21 |  |  | Hyper (>50) | 50-54,XX [12]/106,XXXX[1]/46,XX[1] | ND | UNKN |
| 8 | F/5 | B-ALL | Diagnosis | LR | SEHOP/LAL-SHOP-2005 | CR | CR-A | A | 14 21 |  |  | Hyper (>50) | 46,XX[10] | TEL gain-84% AML1 gain-84% | UNKN |
| 9 | F/5 | B-ALL | Diagnosis | LR | PETHEMA LAL-BR-01 | CR | Rel-CR-A | A | 4 6 10 14 17 18 21 X |  |  | Hyper (>50) | 50-52,XX,+2,+5,+7,+14,+17,+18,+21,+mar[2]/46,XX[18] | AML1 gain-80% | UNKN |
| 10 | F/5 | B-ALL | Diagnosis | IR | PETHEMA-SEHOP/LAL/IR | CR | CR-A | A |  |  |  | Hyper (>50) | 53-56,XX[5]/68-75,XX[2]/46,XX[13] | TEL gain-30% AML1 gain-30% MLL gain-31% ABL gain-25% | UNKN |
| 11 | F/7 | B-ALL | Diagnosis | LR | SEHOP/LAL-SHOP-2005 | Refr | Refr-D | A |  | 13 |  | Hyper (>50) | 46,XX[5] | AML1 gain-14% MLL gain-13% | UNKN |
| 12 | F/8 | B-ALL | Diagnosis | IR | PETHEMA LAL-RI/96 | CR | CR-A | A |  | 7p |  | Hyper (>50) | 61-65,XXX[4]/46,XX[15] | TEL gain-43% AML1 gain-43% ABL gain-43% BCR gain-43% | UNKN |
| 13 | M/9 | B-ALL | Diagnosis | LR | PETHEMA LAL-BR-01 | CR | CR-A | A | 1q 4 6 9p 10 14 17 18 21 X | 8p |  | Hyper (>50) | 46,XY[23] | AML1 gain-95% MLL gain-10% | UNKN |
| 14 | F/10 | B-ALL | Diagnosis | IR | PETHEMA LAL-RI/96 | CR | CR-A | A | 10 | 4p 7 9 16 17 20 |  | Hyper (>50) | 75-90,XXXX[8]/46,XX[4] | AML1 gain-76% BCR gain-80% MLL gain-58% | UNKN |
| 15 | F/12 | B-ALL | Diagnosis | HR | PETHEMA ALL/IR-HR | CR | UNKN | A | 17 21 |  |  | Hyper (>50) | 52-58,XX,del(1)(p21),+add(1)(q31),+5,+6,+8,+9,+10,+11,+17,+18,+19,+22,+mar [16]/46,XX[3] | N | UNKN |
| 16 | M/1 | B-ALL | Diagnosis | HR | BFM | CR | UNKN | A |  |  |  | Hyper (47-50) | 47,XY,+21[3] /46,XY[18] | N | UNKN |
| 17 | F/1 | B-ALL | Diagnosis | IR | LAL/SEHOP-PETHEMA 2013 | CR | CR-A | A | 19p |  |  | Hyper (47-50) | 50,XX,+19,+12,+14,+mar[5]/46,XX[15] | N | UNKN |
| 18 | F/6 | B-ALL | Diagnosis | LR | SEHOP/LAL-SHOP-2005 | CR | CR-A | A | 4 5 6 7q* X | 7p* 16 19 22 |  | Hyper (47-50) | F | AML1 gain-42% | UNKN |
| 19 | F/4 | B-ALL | Diagnosis | IR | LAL/SEHOP-PETHEMA 2013 | CR | CR-ED | A |  |  |  | Hyper (47-50) | 49,XX,+10,+19,+20[7]/46,XX[3] | N | ND |
| 20 | F/4 | B-ALL | Diagnosis | IR | PETHEMA LAL-RI/96 | CR | CR-A | A | 21 X | 9p 20q |  | Hyper (47-50) | F | AML1 gain-100% | UNKN |
| 21 | M/2 | B-ALL | Diagnosis | IR | SEHOP/LAL-SHOP/1994 | CR | CR-A | A | X | 12p |  | Hyper (47-50) | 48,XY,+11,+17[3]/44,XY,-13,-15[2] /46,XY[14] | N | UNKN |
| 22 | F/5 | B-ALL | Diagnosis | IR | PETHEMA LAL-RI/96 | CR | CR-A | A | 21 |  |  | Hyper (47-50) | 46,XX[10] | AML1 gain-32% | UNKN |
| 23 | M/6 | B-ALL | Diagnosis | LR | UNKN | CR | Rel-CR-A | A | 6 4 7q* 21 X | 7p* 19 22 |  | Hyper (47-50) | 50,XY,+1,+4,+21,+2mar[5]/46,XY[5] | ND | UNKN |
| 24 | F/14 | B-ALL | Diagnosis | LR | PETHEMA LAL-BR-01 | CR | Rel-D | A | 18 21 X |  |  | Hyper (47-50) | F | AML1 gain-85% | UNKN |
| 25 | F/2 | B-ALL | Diagnosis | LR | PETHEMA LAL-AR/2005 | CR | CR-A | A | 1q 4 6 14 17 18 21 X |  |  | *ETV6-RUNX1 (TEL-AML1)* | 46,XX[10] | AML1 gain-46% TEL/AML1 31% | UNKN |
| 26 | M/2 | B-ALL | Diagnosis | LR | SEHOP/LAL-SHOP/1994 | CR | CR-A | A |  |  |  | *ETV6-RUNX1 (TEL-AML1)* | 46,XY[10] | TEL/AML1 94% | ND |
| 27 | M/3 | B-ALL | Diagnosis | LR | SEHOP/LAL-SHOP/1999 | CR | CR-A | A |  | 13 |  | *ETV6-RUNX1 (TEL-AML1)* | 46,XY[10] | TEL/AML1 95.5% | UNKN |
| 28 | F/3 | B-ALL | Diagnosis | IR | PETHEMA LAL-RI/96 | CR | CR-A | A | 21 |  |  | *ETV6-RUNX1 (TEL-AML1)* | 46,XX[12] | AML1 gain-63% TEL/AML112% | TEL/AM1 Positive |
| 29 | F/3 | B-ALL | Diagnosis | LR | SEHOP/LAL-SHOP-2005 | CR | CR-A | A |  |  |  | *ETV6-RUNX1 (TEL-AML1)* | 46,XX[20] | TEL/AML1 12% | TEL/AM1 Positive |
| 30 | F/3 | B-ALL | Diagnosis | IR | PETHEMA LAL-RI/96 | CR | UNKN | A |  |  |  | *ETV6-RUNX1 (TEL-AML1)* | 46,XX[10] | TEL/AML1 97.5% | UNKN |
| 31 | M/4 | B-ALL | Diagnosis | LR | SEHOP/LAL-SHOP-2005 | CR | CR-A | A | 21 |  |  | *ETV6-RUNX1 (TEL-AML1)* | 47,XY+21[4]/46,XY[16] | AML1 gain-82% TEL/AML198% | TEL/AM1 Positive |
| 32 | M/4 | B-ALL | Diagnosis | LR | PETHEMA LAL-BR-01 | CR | CR-A | A |  | 8p 12p |  | *ETV6-RUNX1 (TEL-AML1)* | F | TEL loss-100% TEL/AML1 100% | UNKN |
| 33 | F/4 | B-ALL | Diagnosis | IR | PETHEMA LAL/89 | CR | CR-A | A | 17 19 | X |  | *ETV6-RUNX1 (TEL-AML1)* | 46,XX[10] | TEL/AML1 88% | UNKN |
| 34 | F/5 | B-ALL | Diagnosis | LR | BFM 95 | CR | Refr-CR-A | A | 4 6 14 17q*** 18 21 X | 17p*** |  | *ETV6-RUNX1 (TEL-AML1)* | 51-58,XX,+5,+6,+9,+10,+21[14]/46,XX[6] | AML1 gain-89% | TEL/AM1 Positive |
| 35 | M/5 | B-ALL | Diagnosis | IR | LAL/SEHOP-PETHEMA 2013 | CR | CR-A | A | 16 Xq | 6q 13 |  | *ETV6-RUNX1 (TEL-AML1)* | 46,XY[16] | TEL/AML1 97% | UNKN |
| 36 | F/5 | B-ALL | Diagnosis | LR | PETHEMA LAL/89 | CR | CR-A | A | 19 |  |  | *ETV6-RUNX1 (TEL-AML1)* | 46,XX[10] | TEL/AML1 96% | UNKN |
| 37 | M/5 | B-ALL | Diagnosis | LR | SEHOP/LAL-SHOP-2005 | CR | CR-A | A |  |  |  | *ETV6-RUNX1 (TEL-AML1)* | 46,XY[20] | TEL/AML1 87% | UNKN |
| 38 | F/5 | B-ALL | Diagnosis | LR | SEHOP/LAL-SHOP-2005 | CR | CR-A | A |  | X |  | *ETV6-RUNX1 (TEL-AML1)* | F | TEL/AML1 81% MYC Normal | TEL/AM1 Positive |
| 39 | F/8 | B-ALL | Diagnosis | LR | SEHOP/LAL-SHOP-2005 | CR | CR-A | A | 21 | 13 |  | *ETV6-RUNX1 (TEL-AML1)* | F | TEL/AML1 61% | TEL/AM1 Positive |
| 40 | M/9 | B-ALL | Diagnosis | IR | PETHEMA LAL-RI/96 | CR | CR-A | A | 19p |  |  | *ETV6-RUNX1 (TEL-AML1)* | F | TEL/AML1 83% | TEL/AM1 Positive |
| 41 | F/14 | B-ALL | Diagnosis | LR | UNKN | CR | CR-A | A | 21 | 6q |  | *ETV6-RUNX1 (TEL-AML1)* | ND | TEL/AML1 96% | TEL/AM1 Positive |
| 42 | M/14 | B-ALL | Diagnosis | LR | BFM | CR | CR-A | A | 21 |  |  | *ETV6-RUNX1 (TEL-AML1)* | 46,XY[15] | TEL gain-33% AML1 gain-76% TEL/AML180% MLL gain-26% ABL gain-48% BCR gain-60% | TEL/AM1 Positive |
| 43 | F/15 | B-ALL | Diagnosis | LR | BFM | CR | CR-A | N |  |  |  | *ETV6-RUNX1 (TEL-AML1)* | 47,XX,+21c[15]/46,XX[3] | AML1 gain-100% TEL/AML188.5% | UNKN |
| 44 | F/8 | B-ALL | Diagnosis | HR | PETHEMA LAL-AR/2003 with Imatinib | CR | Rel-D | N |  |  |  | *BCR-ABL1* | 46,XX,t(9;22)(q34;q11)[18] | BCR/ABL 82% | Minor BCR/ABL Positive |
| 45 | F/9 | B-ALL | Diagnosis | HR | European Pediatric Protocol/ALL-Ph+ | CR | CR-A | A |  |  |  | *BCR-ABL1* | F | MLL gain-5% | BCR/ABL Positive |
| 46 | F/0 | B-ALL | Diagnosis | HR | SEHOP/LAL-SHOP/1999 | CR | CR-A | A |  |  |  | *KMT2A(MLL)-R* | 46,XX,del(1)(q31),t(4;11)(q21;q23)[5]/46,XX[5] | KMT2A(MLL)-R 83% | UNKN |
| 47 | F/0 | B-ALL | Diagnosis | HR | PETHEMA LAL-AR/2005 | CR | Rel-D | N |  |  |  | *KMT2A(MLL)-R* | 47,XX,t(4;11)(q21;q23),+mar[23] | KMT2A(MLL)-R 83% | UNKN |
| 48 | F/0 | B-ALL | Diagnosis | HR | PETHEMA LAL-AR/2005 | CR | CR-A | A |  |  |  | *KMT2A(MLL)-R* | 46,XX,inv(11)(q12q23),del(12)(p13)[7]/46,XX[13] | KMT2A(MLL)-R 93% | UNKN |
| 49 | F/0 | B-ALL | Diagnosis | HR | BFM | NE | UNKN | A |  |  |  | *KMT2A(MLL)-R* | 40-42,XX[6]/46,XX[6] | KMT2A(MLL)-R 18% | MLL/AF4 Positive |
| 50 | M/14 | B-ALL | Diagnosis | HR | SEHOP/LAL-SHOP/1999 | CR | CR-D | A |  |  |  | *KMT2A(MLL)-R* | 38-40,XY[6]/46,XY[6] | KMT2A(MLL)-R 96% | ND |
| 51 | M/16 | B-ALL | Diagnosis | HR | PETHEMA LAL-AR/2011 | CR | CR-A | A |  | 17 19 22 |  | *KMT2A(MLL)-R* | 46,XY,t(4;11)(q21;q23)[7]/46,XY[3] | KMT2A(MLL)-R 83% | MLL/AF4 Positive |
| 52 | M/5 | B-ALL | Diagnosis | LR | BFM 95 | CR | Refr-CR-A | A | 1q | X |  | *TCF3(E2A)-PBX1* | F | TCF3/PBX1 75% | E2A/PBX1 Positive |
| 53 | F/12 | B-ALL | Diagnosis | HR | PETHEMA LAL-AR/2003 | CR | Rel-D | A | 1q | 9p 17p |  | *TCF3(E2A)-PBX1* | 46,XX[15] | TCF3 loss-89% TCF3/PBX1 89% | ND |
| 54 | M/14 | B-ALL | Diagnosis | IR | PETHEMA LAL-RI/96 | CR | CR-A | A |  |  |  | *TCF3(E2A)-PBX1* | 46,XY[10] | N | E2A/PBX1 Positive |
| 55 | M/4 | B-ALL | Diagnosis | IR | PETHEMA LAL-RI/96 | CR | CR-A | A | 18 21 X | 9p 20q |  | Others | 47,XY,+mar (C or D)[8]/46,XY[2] | ND | UNKN |
| 56 | M/4 | B-ALL | Diagnosis | LR | SEHOP/LAL/SHOP-2005 | CR | CR-A | A | 4 6p 10 14 18 21 X | 13 |  | Others | ND | AML1 gain-95% | UNKN |
| 57 | F/5 | B-ALL | Diagnosis | LR | SEHOP/LAL-SHOP-2005 | CR | CR-A | A | 4 6p 10 14 17 18 21 |  |  | Others | ND | AML1 gain-43% | N |
| 58 | M/4 | B-ALL | Diagnosis | LR | UNKN | UNKN | UNKN | A |  |  |  | Others | 46,XY,add(14)(q32)[4]/46,XY[6] | ND | UNKN |
| 59 | M/5 | B-ALL | Diagnosis | LR | BFM 95 | CR | Refr-CR-Rel | A |  |  |  | Others | 46,XY,add(12)(q22)[16] /46,XY[4] | ND | UNKN |
| 60 | F/6 | B-ALL | Diagnosis | LR | PETHEMA LAL-BR-01 | CR | CR-A | A | 10 14 17 21 |  |  | Others | 46,XX,add(3)(q)(21)[2]/46,XX[6] | ND | UNKN |
| 61 | F/7 | B-ALL | Diagnosis | IR | PETHEMA LAL-RI/96 | CR | CR-A | A |  | 9p |  | Others | 46,XX,del(6)(q15q23)[6]/46,XX[4] | ND | UNKN |
| 62 | F/15 | B-ALL | Diagnosis | IR | PETHEMA LAL-RI/96 | CR | Rel-D | A | 7q**** | 7p**** 19 |  | Others | 46,XX,t(7;15)(p13;q12)[3] /46,XX[7] | N | ND |
| 63 | F/15 | B-ALL | Diagnosis | LR | Hoelzer protocol | CR | CR-A | A |  | X |  | Others | 46,XX,t(3;17)(q13;p13)[10]/46,XX[2] | ND | UNKN |
| 64 | F/UNKN | B-ALL | Diagnosis | HR | PETHEMA LAL-AR/93 | NA | ED | A |  | 18 X |  | Normal | 46,XX,?inv(3)(p13;q21)[1]/46,XX[20] | ND | ND |
| 65 | F/0 | B-ALL | Diagnosis | HR | PETHEMA LAL-AR/93 | CR | CR-A | A |  |  |  | Normal | 46,XX[23] | N | UNKN |
| 66 | M/UNKN | B-ALL | Diagnosis | IR | UNKN | CR | CR-A | N |  |  |  | Normal | 46,XY[20] | N | UNKN |
| 67 | F/1 | B-ALL | Diagnosis | IR | LAL/SEHOP-PETHEMA 2013 | CR | CR-A | A | 4 5 6 8 10 14 17 18 21 |  |  | Normal | 46,XX[20] | ND | UNKN |
| 68 | F/1 | B-ALL | Diagnosis | IR | PETHEMA LAL-RI/96 | CR | CR-A | A |  | 7p |  | Normal | chrb 46,XX[5] /46,XX[15] | N | UNKN |
| 69 | M/1 | B-ALL | Diagnosis | HR | SEHOP/LAL-SHOP-2005 | CR | CR-A | A |  |  |  | Normal | F | N | N |
| 70 | F/1 | B-ALL | Diagnosis | IR | SEHOP/LAL/SHOP-2005 | CR | CR-A | A | 1q |  |  | Normal | 46,XX[14] | N | N |
| 71 | M/1 | B-ALL | Diagnosis | LR | UNKN | CR | CR-A | A |  | 9p X |  | Normal | 46,XY[12] | ND | UNKN |
| 72 | F/2 | B-ALL | Diagnosis | LR | PETHEMA LAL/89 | CR | CR-A | A |  | X |  | Normal | 46,XX[10] | ND | UNKN |
| 73 | M/2 | B-ALL | Diagnosis | HR | BFM | CR | UNKN | A |  | 9p |  | Normal | 46,XY[12] | N | ND |
| 74 | F/2 | B-ALL | Diagnosis | LR | BFM | CR | UNKN | A | X | 20q |  | Normal | 46,XX[12] | N | ND |
| 75 | M/3 | B-ALL | Diagnosis | LR | PETHEMA LAL-BR-01 | CR | CR-A | A | 4 10 14 18 21 X |  |  | Normal | 46,XY[25] | N | UNKN |
| 76 | F/4 | B-ALL | Diagnosis | LR | SEHOP/LAL-SHOP/1999 | CR | CR-A | A |  |  |  | Normal | 46,XX[21] | N | N |
| 77 | F/3 | B-ALL | Diagnosis | LR | PETHEMA LAL-BR-01 | CR | CR-A | A |  |  |  | Normal | 46,XX[14] | N | UNKN |
| 78 | F/3 | B-ALL | Diagnosis | LR | PETHEMA LAL-BR-01 | CR | CR-A | A |  | 12p |  | Normal | F | N | UNKN |
| 79 | M/3 | B-ALL | Diagnosis | LR | PETHEMA LAL-BR-01 | CR | CR-A | A | X |  |  | Normal | F | AML1 gain-82% | N |
| 80 | M/4 | B-ALL | Diagnosis | IR | PETHEMA LAL-RI/96 | CR | CR-A | N |  |  |  | Normal | 46,XY[10] | N | ND |
| 81 | M/4 | B-ALL | Diagnosis | LR | SEHOP/LAL-SHOP-2005 | CR | CR-A | A |  |  |  | Normal | 46,XY[21] | N | ND |
| 82 | F/4 | B-ALL | Diagnosis | LR | UNKN | UNKN | A | A |  |  |  | Normal | 46,XX[13] | N | ND |
| 83 | M/4 | B-ALL | Diagnosis | LR | UNKN | CR | CR-A | A |  |  |  | Normal | 46,XY[20] | N | UNKN |
| 84 | M/4 | B-ALL | Diagnosis | LR | PETHEMA LAL-BR-01 | CR | CR-A | A | 17q |  |  | Normal | 47,XY,+21c[15] | AML1 gain 97.5% | UNKN |
| 85 | F/4 | B-ALL | Diagnosis | IR | PETHEMA LAL-RI/96 | CR | Rel-D | A | 1q 4 6 7q* 9 10 14 17 21 | 7p* |  | Normal | 46,XX[10] | N | ND |
| 86 | M/4 | B-ALL | Diagnosis | LR | UNKN | UNKN | UNKN | A |  | 9p 20q |  | Normal | F | N | UNKN |
| 87 | M/5 | B-ALL | Diagnosis | LR | SEHOP/LAL-SHOP/1999 | CR | CR-A | A |  |  |  | Normal | 46,XY[10] | N | ND |
| 88 | F/5 | B-ALL | Diagnosis | LR | SEHOP/LAL-SHOP/1999 | CR | CR-A | A |  |  |  | Normal | F | N | UNKN |
| 89 | F/5 | B-ALL | Diagnosis | IR | PETHEMA LAL-RI/96 | CR | CR-A | A |  |  |  | Normal | 46,XY[8] | ND | UNKN |
| 90 | F/5 | B-ALL | Diagnosis | IR | LAL/SEHOP-PETHEMA IR 2012 | CR | CR-A | A |  |  |  | Normal | 38-40,XX[5] (Uninformative karyotype) | N | UNKN |
| 91 | M/5 | B-ALL | Diagnosis | LR | SHOP/LLA-BR/2001 | CR | Rel-CR-A | A |  |  |  | Normal | F | N | UNKN |
| 92 | F/6 | B-ALL | Diagnosis | LR | PETHEMA LAL-BR-01 | CR | CR-A | A |  |  |  | Normal | F | N | N |
| 93 | F/6 | B-ALL | Diagnosis | IR | PETHEMA LAL/89 | CR | CR-A | N |  |  |  | Normal | 46,XX[10] | N | UNKN |
| 94 | M/6 | B-ALL | Diagnosis | LR | PETHEMA LAL/89 | CR | CR-A | A |  | 6q 8p |  | Normal | F | N | ND |
| 95 | F/6 | B-ALL | Diagnosis | IR | PETHEMA LAL-RI/96 | CR | CR-A | A |  | 13 |  | Normal | 46,XX[22] | N | UNKN |
| 96 | F/6 | B-ALL | Diagnosis | IR | PETHEMA LAL-RI/96 | CR | CR-A | N |  |  |  | Normal | 46,XX[10] | N | UNKN |
| 97 | F/7 | B-ALL | Diagnosis | IR | SEHOP/LAL-SHOP-2005 | CR | Refr-CR-A | A | 14 21 | 13 |  | Normal | 46,XX[21] | N | N |
| 98 | M/7 | B-ALL | Diagnosis | LR | SEHOP/LAL-SHOP-2005 | CR | Rel-CR-A | A |  | 9p |  | Normal | 46,XY[22] | N | N |
| 99 | M/7 | B-ALL | Diagnosis | LR | UNKN | CR | CR-A | A |  | 12p 18p |  | Normal | F | N | UNKN |
| 100 | F/8 | B-ALL | Diagnosis | HR | PETHEMA LAL-Ph/2000 | CR | Refr-CR-A | A |  |  |  | Normal | 46,XX[10] | ND | UNKN |
| 101 | M/8 | B-ALL | Diagnosis | HR | PETHEMA LAL-AR/2005 | CR | CR-A | A |  |  |  | Normal | 46,XY[10] | N | N |
| 102 | M/9 | B-ALL | Diagnosis | IR | PETHEMA LAL-RI/96 | CR | CR-A | A |  |  |  | Normal | 46,XY[10] | N | UNKN |
| 103 | F/10 | B-ALL | Diagnosis | HR | BFM | PR | A | A |  |  |  | Normal | 46,XX[8] | N | N |
| 104 | M/10 | B-ALL | Diagnosis | IR | PETHEMA LAL-RI/96 | CR | Rel-D | A | 19p |  |  | Normal | 46,XY[12] | N | UNKN |
| 105 | M/12 | B-ALL | Diagnosis | LR | SEHOP/LAL/SHOP-97 | CR | Rel-D | A |  |  |  | Normal | 46,XY[13] | ND | UNKN |
| 106 | M/14 | B-ALL | Diagnosis | IR | PETHEMA LAL-RI/96 | CR | CR-ED | A |  |  |  | Normal | F | N | N |
| 107 | F/14 | B-ALL | Diagnosis | HR | LAL/SEHOP-PETHEMA 2013 | Refr | Refr-D | A |  |  |  | Normal | 46,XX[10] | N | ND |
| 108 | M/17 | B-ALL | Diagnosis | IR | PETHEMA LAL-RI/96 | CR | CR-A | A | 21 |  |  | Normal | 46,XY[10] | ND | ND |
| 109 | M/16 | B-ALL | Diagnosis | HR | UNKN | CR | Rel-CR-A | A | 10 |  |  | Normal | 46,XY[10] | ND | UNKN |
| 110 | F/16 | B-ALL | Diagnosis | HR | PETHEMA LAL-AR/2003 | CR | CR-A | A |  | 7p 9p |  | Normal | F | N | N |
| 111 | M/16 | B-ALL | Diagnosis | HR | PETHEMA LAL-AR/93 | CR | Refr-CR-A | A | 10q 14 17 18 21 22 X |  |  | Normal | 46,XY[10] | N | ND |
| 112 | M/16 | B-ALL | Diagnosis | IR | PETHEMA LAL-RI/2008 | CR | CR-A | A | 14 |  |  | Normal | 46,XX[12] | N | N |
| 113 | M/17 | B-ALL | Diagnosis | IR | PETHEMA LAL-RI/2008 | CR | CR-A | A |  | 7p 9p 12p |  | Normal | 46,XY[12] | N | N |
| 114 | F/17 | B-ALL | Diagnosis | HR | PETHEMA LAL-AR/93 | CR | Rel-CR-D | A | 5p 10 | 17 19 |  | Normal | 46,XX[10] | N | ND |
| 115 | F/17 | B-ALL | Diagnosis | IR | PETHEMA LAL-RI/1995 | CR | CR-A | A | 21 | 12p |  | Normal | ND | N | ND |
| 116 | F/19 | B-ALL | Diagnosis | HR | PETHEMA LAL Ph/2008 | CR | CR-A | A |  |  |  | *BCR-ABL1* | 80,XXXX[1]/46,XX[12] | BCR/ABL 78% | Major b2-a3 BCR/ABL Positive |
| 117 | M/19 | B-ALL | Diagnosis | HR | PETHEMA LAL Ph/2008 | CR | CR-A | A |  | 7 |  | *BCR-ABL1* | 46,XY,t(9;22)(q34;q11)[15]/45,XY,-7,t(7;9;22)(q21;q34;q11),add(15)(q21)[3] | BCR/ABL 86% | ND |
| 118 | F/24 | B-ALL | Diagnosis | HR | PETHEMA LAL Ph/2008 | NA | ED | A |  |  |  | *BCR-ABL1* | 48,XX,t(9;22)(q34;q11),+17,+21[6] /46,XX[14] | BCR/ABL 68% | Minor BCR/ABL Positive |
| 119 | M/27 | B-ALL | Diagnosis | HR | UNKN | CR | D | A | 2 5 8 10 22 |  |  | *BCR-ABL1* | 53,XY,+2,+5,+8,t(9;22)(q34;q11),+10,+13,+20,+der(9)t(9;22)(q34;q11)[15] | ND | ND |
| 120 | M/29 | B-ALL | Diagnosis | HR | PETHEMA LAL-Ph/2000/Imatinib | CR | Rel-D | A |  |  |  | *BCR-ABL1* | 46,XY,t(9;22)(q34;q11)[10] | BCR/ABL 91% | Major BCR/ABL Positive |
| 121 | M/29 | B-ALL | Diagnosis | HR | PETHEMA LAL Ph/2008 | CR | CR-D | A | 7q* | 7p* |  | *BCR-ABL1* | 48,XY,+5,add(7)(p13),t(9;22)(q34;q11),+der(22)t(9;22)(q34;q11/46,XY[5] | BCR/ABL 94%. | Minor BCR/ABL Positive |
| 122 | M/30 | B-ALL | Diagnosis | HR | PETHEMA LAL Ph/2008 | CR | CR-A | A |  | 8p 9p |  | *BCR-ABL1* | F | ABL loss-28% BCR/ABL 97% | UNKN |
| 123 | M/34 | B-ALL | Diagnosis | HR | PETHEMA LAL Ph-2008/Clinical Trial CSTI571BES02 | CR | CR-A | A |  |  |  | *BCR-ABL1* | F | BCR/ABL 98.5% | Major BCR/ABL Positive |
| 124 | F/34 | B-ALL | Diagnosis | HR | PETHEMA LAL-Ph/2000/Imatinib | PR (CR later on) | Rel-D | A |  | 17 19 |  | *BCR-ABL1* | 46,XX,t(9;22)(q34;q11)[8]/46,XX[2] | BCR/ABL 82% | Minor BCR/ABL Positive |
| 125 | M/34 | B-ALL | Diagnosis | HR | PETHEMA LAL Ph/2008 | CR | CR-A | A | 17 X |  |  | *BCR-ABL1* | 46,XY,t(9;22)(q34;q11)[7] /46,XY[5] | ND | Minor BCR/ABL Positive |
| 126 | M/37 | B-ALL | Diagnosis | HR | PETHEMA LAL-AR/93 | CR | Rel-D | A |  | 7p 9p |  | *BCR-ABL1* | 46,XY[8] | BCR/ABL 80% | BCR/ABL Positive |
| 127 | M/40 | B-ALL | Diagnosis | HR | PETHEMA LAL Ph/2008 | CR | CR-A | A |  |  |  | *BCR-ABL1* | F | BCR/ABL 14% | ND |
| 128 | M/40 | B-ALL | Diagnosis | HR | Second-generation TKI | UNKN | Rel-CR-A | A |  |  |  | *BCR-ABL1* | 46,XY[20] | N | Major BCR/ABL Positive |
| 129 | F/45 | B-ALL | Diagnosis | HR | PETHEMA LAL Ph/2008 | CR | CR-ED | A | 2 4 6 10 14 21 | 12p |  | *BCR-ABL1* | F | BCR/ABL 98% | Minor BCR/ABL Positive |
| 130 | M/46 | B-ALL | Diagnosis | HR | PETHEMA LAL Ph/2008 | CR | CR-D | A |  | 7p 9p |  | *BCR-ABL1* | F | BCR/ABL 76% | Major BCR/ABL Positive |
| 131 | M/47 | B-ALL | Diagnosis | HR | PETHEMA LAL-AR/93 | Refr | Refr-D | A | 8 |  |  | *BCR-ABL1* | 46,XY,t(9;22)(q34;q11)[7]/47,XY,+8,t(9;22)(q34;q11)[2]/46,XY[5] | BCR/ABL 65% | ND |
| 132 | M/51 | B-ALL | Diagnosis | HR | PETHEMA LAL Ph/2008 | NA | ED | A | 17q*** | 17p*** |  | *BCR-ABL1* | F | BCR/ABL 93% | UNKN |
| 133 | F/52 | B-ALL | Diagnosis | HR | PETHEMA LAL Ph-2007/ PETHEMA LAL Ph-2008 | CR | CR-A | A |  | 7 |  | *BCR-ABL1* | 46,XX,t(9;22)(q34;q11)[15]/46,XX[5] | BCR/ABL 71% | UNKN |
| 134 | M/57 | B-ALL | Diagnosis | HR | PETHEMA LAL Ph/2008 | CR | CR-A | A |  | 11q |  | *BCR-ABL1* | F | BCR/ABL 64% MLL loss-61% | UNKN |
| 135 | M/58 | B-ALL | Diagnosis | HR | PETHEMA LAL-07OLD | UNKN | Rel-D | A |  |  |  | *BCR-ABL1* | 46,XY,t(9;22)(q34;q11)[11]/46,XY[9] | BCR/ABL 87% | UNKN |
| 136 | F/59 | B-ALL | Diagnosis | HR | LAL-070PH | CR | CR-A | A |  |  |  | *BCR-ABL1* | 46,XX,t(9;22)(q34;q11)[5]/46,XX[10] | ABL loss-77% BCR/ABL 77% | UNKN |
| 137 | M/61 | B-ALL | Diagnosis | HR | UNKN | CR | CR-D | A |  |  |  | *BCR-ABL1* | 46,XY,t(4;12)(p13;q12),t(9;22)(q34;q11)[15]/46,XY[5] | BCR/ABL 81% | UNKN |
| 138 | F/64 | B-ALL | Diagnosis | HR | UNKN | UNKN | A | A | X |  |  | *BCR-ABL1* | 46,XX,t(9;22)(q34;q11)[22] | BCR/ABL 88% | UNKN |
| 139 | M/65 | B-ALL | Diagnosis | HR | PETHEMA LAL-Ph/2000/Imatinib | CR | Refr-ED | N |  |  |  | *BCR-ABL1* | 46,XY,t(9;22)(q34;q11)[5]/46,XY[2] | BCR/ABL 94% | UNKN |
| 140 | M/66 | B-ALL | Diagnosis | HR | PETHEMA LAL-Ph/2000 | NA | ED | A | 8q 19 |  |  | *BCR-ABL1* | 47,XY,+2,del(6)(q21),t(9;22)(q34;q11)[20] | BCR/ABL 62% | UNKN |
| 141 | M/70 | B-ALL | Diagnosis | HR | PETHEMA LAL-RI/96 with Glivec | Refr | Refr-ED | A |  |  |  | *BCR-ABL1* | 46,XY,t(9;22)(q34;q11)[8]/46,XY[2] | BCR/ABL 87% | UNKN |
| 142 | M/70 | B-ALL | Diagnosis | HR | No therapy | No therapy | NT-ED | A |  |  |  | *BCR-ABL1* | 46,XY,t(9;22)(q34;q11)[13]/46,XY[8] | BCR/ABL 93% | UNKN |
| 143 | M/70 | B-ALL | Diagnosis | HR | PETHEMA LAL-AR/89 | Refr | Refr-ED | A | 19 |  |  | *BCR-ABL1* | F | BCR/ABL 54% | UNKN |
| 144 | F/71 | B-ALL | Diagnosis | HR | No therapy | No therapy | NT-ED | A |  |  |  | *BCR-ABL1* | F | BCR/ABL 92% | UNKN |
| 145 | F/71 | B-ALL | Diagnosis | HR | UNKN | UNKN | UNKN | A |  |  |  | *BCR-ABL1* | ND | BCR/ABL 80% | UNKN |
| 146 | M/76 | B-ALL | Diagnosis | HR | PETHEMA LAL-07FRAIL/EWALL-PH-01 | CR | CR-D | A | 4 18 X | 19 |  | *BCR-ABL1* | 55-60,XY,+Y,+4,+5,+7,+8,t(9;22)(q34;q11),+9,+11,add(12)(p12)[15] | ABL gain-89% BCR/ABL 89% MLL gain-28% | UNKN |
| 147 | M/84 | B-ALL | Diagnosis | HR | No therapy | No therapy | NT-ED | A | Xp | 9 |  | *BCR-ABL1* | F | BCR/ABL 78% | UNKN |
| 148 | M/UNKN | B-ALL | Diagnosis | HR | UNKN | Refr | Refr | A |  | 9 |  | *BCR-ABL1* | 46,XY[6] | BCR/ABL 82.5% | ND |
| 149 | F/23 | B-ALL | Diagnosis | HR | PETHEMA LAL-AR/2003 | CR | Rel-D | A |  |  |  | *KMT2A(MLL)-R* | 46,XX,t(4;11)(q21;q23)[17] /46,XX[4] | KMT2A(MLL)-R 65% | UNKN |
| 150 | F/31 | B-ALL | Diagnosis | HR | GMALL | CR | Rel-D | A |  |  |  | *KMT2A(MLL)-R* | 46,XX,t(4;11)(q21;q23)[10] | N | UNKN |
| 151 | M/35 | B-ALL | Diagnosis | HR | PETHEMA LAL-AR/2003 | CR | Rel-CR-A | N |  |  |  | *KMT2A(MLL)-R* | 46,XY,t(11;19)(q23;p13)[10]/46,XY[5] | KMT2A(MLL)-R 81.5% | UNKN |
| 152 | F/41 | B-ALL | Diagnosis | HR | PETHEMA LAL-AR/2003 | CR | CR-ED | N |  |  |  | *KMT2A(MLL)-R* | F | KMT2A(MLL)-R 81.5% | UNKN |
| 153 | M/46 | B-ALL | Diagnosis | HR | PETHEMA LAL Ph/2008 | CR | CR-D | N |  |  |  | *KMT2A(MLL)-R* | F | KMT2A(MLL)-R 95.5% | UNKN |
| 154 | F/47 | B-ALL | Diagnosis | HR | PETHEMA LAL-AR/93 | UNKN | D | A |  |  |  | *KMT2A(MLL)-R* | 46,XX,t(4;11)(q21;q23)[14] | N | UNKN |
| 155 | M/55 | B-ALL | Diagnosis | HR | PETHEMA LAL/89 | NA | ED | A |  |  |  | *KMT2A(MLL)-R* | 46,XY,t(4;11)(q21;q23)[3] /46,XY[10] | KMT2A(MLL)-R 75% | UNKN |
| 156 | M/60 | B-ALL | Diagnosis | HR | PETHEMA LAL-AR/2003 | CR | CR-ED | A | 6 |  |  | *KMT2A(MLL)-R* | F | KMT2A(MLL)-R 73% | UNKN |
| 157 | F/69 | B-ALL | Diagnosis | HR | LAL-070PH | CR | UNKN | N |  |  |  | *KMT2A(MLL)-R* | 46,XX,t(4;11)(q21;q23)[9]/46,XX[11] | KMT2A(MLL)-R 60% | UNKN |
| 158 | M/74 | B-ALL | Diagnosis | HR | PETHEMA LAL-07OLD | CR | Rel-D | N |  |  |  | *KMT2A(MLL)-R* | ND | KMT2A(MLL)-R 39% | UNKN |
| 159 | F/80 | B-ALL | Diagnosis | HR | PETHEMA LAL/OLD-FRA | CR | Rel-D | N |  |  |  | *KMT2A(MLL)-R* | 46,XX[15] | KMT2A(MLL)-R 93% | UNKN |
| 160 | M/19 | B-ALL | Diagnosis | IR | PETHEMA LAL-IR/2011 | CR | Rel-CR-D | A |  | 7 |  | Hypo (44-45) | 45,XY,-7[14]/46,XY[6] | Chr7 loss 72% | UNKN |
| 161 | M/20 | B-ALL | Diagnosis | IR | UNKN | CR | CR-A | N |  |  |  | Hypo (44-45) | 44,XY,-21,-22[4] /46,XY[9] | N | UNKN |
| 162 | M/34 | B-ALL | Diagnosis | HR | PETHEMA LAL-AR/2003 | CR | CR-A | A |  |  |  | Hypo (<44) | 39-42,XY[cp12] | N | UNKN |
| 163 | M/36 | B-ALL | Diagnosis | HR | PETHEMA LAL-AR/93 | PR (CR later on) | Rel-D | N |  |  |  | Hypo (<44) | 34-44,XY[8]/46,XY[9] | ND | UNKN |
| 164 | F/52 | B-ALL | Diagnosis | HR | UNKN | CR | Rel-D | A |  | 9p |  | Hypo (<44) | 39-42,XX,add(12)(p13)[5]/46,XX,add(12)(p13)[2]/46,XX[9] | ND | ND |
| 165 | F/71 | B-ALL | Diagnosis | HR | PETHEMA LAL/89 | CR | Rel-D | N |  |  |  | Hypo (<44) | 33-40,XX,add(4)(q33)[9] /46,XX[1] | ND | UNKN |
| 166 | M/18 | B-ALL | Diagnosis | IR | PETHEMA LAL-RI/2008 | CR | CR-A | A | 4 9q 21 X | 8p |  | Hyper (>50) | F | AML1 gain-39% ABL gain-41% | UNKN |
| 167 | M/29 | B-ALL | Diagnosis | IR | UNKN | CR | CR-ED | A | 1 6 10 11 12 18 19 21 22 X | 3 5 7 8 9 13 15 16 17 20 |  | Hyper (>50) | 57-66,XY[2]/46,XY[18] | BCR gain-71% MLL gain-68% | UNKN |
| 168 | F/31 | B-ALL | Diagnosis | IR | UNKN | NA | ED | A | 6 19 21 22 |  |  | Hyper (>50) | 58-62,XX,+1,+2,+3,+5,+10,+10,+11,+12,+14,+16,+20,+21,+21,+22[17] /46,XX[3] | ABL gain-71% MLL gain-54% | UNKN |
| 169 | F/47 | B-ALL | Diagnosis | HR | PETHEMA Burkimab-08 | ND | Rel-CR-A | A | 6 8 13 14 19 21 | 17q X |  | Hyper (>50) | 46,XX[10] | AML1 gain 90% ETO gain 90% MYC gain 62% IGH gain 60% | UNKN |
| 170 | M/58 | B-ALL | Diagnosis | HR | PETHEMA LAL-AR/2003 | CR | Rel-CR-D | A | 1 8 19 21 X | 3 7 9 12 13 |  | Hyper (>50) | F | BCR gain-42% MLL gain-60% | UNKN |
| 171 | M/61 | B-ALL | Diagnosis | HR | UNKN | CR | Rel-D | A | 1 2 6 10 11 12 14 18 21 X | 7 9 13 15 16 17 20 |  | Hyper (>50) | F | BCR gain-79% MLL gain-85.5% | UNKN |
| 172 | F/38 | B-ALL | Diagnosis | HR | PETHEMA LAL-AR/2003 | CR | Rel-CR-D | A |  |  |  | Hyper (47-50) | 46,XX[10] | ABL gain-30% | N |
| 173 | M/58 | B-ALL | Diagnosis | HR | PETHEMA LAL-AR/93 | CR | Rel-D | A |  | 12p |  | Hyper (47-50) | 47,XY,+18[4]/46,XY[16] | ND | UNKN |
| 174 | F/22 | B-ALL | Diagnosis | HR | PETHEMA LAL-AR 2011 | CR | CR-A | A | 1q |  |  | TCF3(E2A)-PBX1 | F | N | E2A/PBX1 Positive |
| 175 | M/23 | B-ALL | Diagnosis | IR | PETHEMA LAL-RI/96 | CR | CR-A | A | 1q |  |  | TCF3(E2A)-PBX1 | 46,XY,der(19)t(1;19)(q23;p13)[2] | N | ND |
| 176 | F/36 | B-ALL | Diagnosis | HR | PETHEMA LAL-AR/2003 | CR | CR-A | A |  | 13q |  | TCF3(E2A)-PBX1 | F | TCF3/PBX1 75% | E2A/PBX1 Positive |
| 177 | M/25 | B-ALL | Diagnosis | IR | ALL Adult protocol-Adaptation of GMALL05/93 protocol | NA | ED | A |  | 8p | cth 15q | Others | 47,XY,del(14)(q12),+21c[8]/47,XY,+21c[17] | N | UNKN |
| 178 | F/41 | B-ALL | Diagnosis | HR | PETHEMA LAL-AR/2011 | CR | Refr-CR-A | N |  |  |  | Others | 47,XX,+mar[7]/46,XX[10] | N | UNKN |
| 179 | F/57 | B-ALL | Diagnosis | HR | PETHEMA LAL-AR/93 | CR | CR-D | A | 5 9q** | 9p** 20p | cth 6 | Others | 45,XX,del(1)(p13p31),-4,del(7)(q21q31),i(9)(q10),der(14) add(14)(q32),-15,+r(15)[cp7]/46,XX[3] | ND | ND |
| 180 | M/64 | B-ALL | Diagnosis | HR | PETHEMA LAL-AR/2003 | CR | Rel-CR-D | A | 8 | 17q 19 |  | Others | 47,XY,+mar[4]/46,XY[15] | N | N |
| 181 | M/77 | B-ALL | Diagnosis | HR | No therapy | No therapy | NT-ED | A | 4 | 6q 8p |  | Others | 47,XY,+4,-6,i(6)(p10),add(11)(q23),+mar[8]/46,XY[12] | N | ND |
| 182 | F/23 | B-ALL | Diagnosis | IR | PETHEMA LAL-RI/2008 | CR | Rel-CR-A | A | 5p 9q** | 9p** |  | Others | 46,XX,del(1)(q21),i(9)(q10)[15] /46,XX[5] | ABL gain-29% | N |
| 183 | M/41 | B-ALL | Diagnosis | HR | NHL 2002 GMALL | CR | CR-A | A |  | X |  | Others | 46,XY,dup(1)(q21q41)[20] | N | N |
| 184 | M/20 | B-ALL | Diagnosis | HR | PETHEMA LAL-AR/93 | PR (CR later on) | Rel-D | A | X |  |  | Normal | F | N | ND |
| 185 | M/22 | B-ALL | Diagnosis | HR | PETHEMA LAL-AR/2003 | CR | CR-A | N |  |  |  | Normal | F | N | UNKN |
| 186 | M/22 | B-ALL | Diagnosis | IR | PETHEMA LAL-RI/2008 | CR | CR-A | A |  | 7 |  | Normal | 46,XY[20] | N | N |
| 187 | M/23 | B-ALL | Diagnosis | IR | UNKN | CR | UNKN | A | 1q |  |  | Normal | F | N | N |
| 188 | M/27 | B-ALL | Diagnosis | IR | UNKN | CR | Rel-CR-A | A |  |  |  | Normal | ND | N | UNKN |
| 189 | M/27 | B-ALL | Diagnosis | HR | PETHEMA LAL-AR/2003 | CR later on | Ref-Rel-CR-A | A | 21 |  |  | Normal | F | N | N |
| 190 | M/31 | B-ALL | Diagnosis | HR | PETHEMA LAL-AR/2003 | CR | CR-A | A | 19p |  |  | Normal | F | N | N |
| 191 | M/32 | B-ALL | Diagnosis | HR | FLAG-IDA | PR (CR later on) | Rel-CR-D | A | 21 X |  |  | Normal | 46,XY[19] | N | ND |
| 192 | M/34 | B-ALL | Diagnosis | HR | PETHEMA LAL-AR/2003 | Refr | Refr-D | A |  |  |  | Normal | 46,XY[15] | N | N |
| 193 | F/36 | B-ALL | Diagnosis | HR | PETHEMA LAL-AR/2011 | CR | CR-A | A |  |  |  | Normal | F | N | N |
| 194 | M/37 | B-ALL | Diagnosis | HR | PETHEMA LAL-AR/2003 | CR | Rel-CR-A | A | 19p |  |  | Normal | F | N | ND |
| 195 | M/38 | B-ALL | Diagnosis | HR | GMALL | CR | Rel-D | N |  |  |  | Normal | 46,XY[10] | N | N |
| 196 | F/39 | B-ALL | Diagnosis | HR | PETHEMA LAL-AR/2003 | CR | CR-A | A |  | 7 |  | Normal | F | N | ND |
| 197 | F/39 | B-ALL | Diagnosis | HR | PETHEMA LAL-AR/2003 | CR | Rel-CR-D | A | 1q 10p 19p | 9p 17p |  | Normal | 46,XX[22] | N | N |
| 198 | M/40 | B-ALL | Diagnosis | HR | PETHEMA LAL-AR/2003 | CR | CR-A | A | 10 |  |  | Normal | F | N | N |
| 199 | M/40 | B-ALL | Diagnosis | HR | No therapy | No therapy | NT-D | A |  |  |  | Normal | F | AML1 gain-95.5% | ND |
| 200 | F/42 | B-ALL | Diagnosis | HR | PETHEMA LAL-AR/2003 | CR | CR-A | A | 4 5p 6 18 21 X | 17p |  | Normal | F | N | N |
| 201 | F/42 | B-ALL | Diagnosis | HR | PETHEMA LAL-AR/93 | Refr | Refr-ED | A |  | 17 19 22 |  | Normal | 46,XX[15] | N | ND |
| 202 | F/43 | B-ALL | Diagnosis | HR | UNKN | CR | CR-A | A | 19p |  |  | Normal | F | N | ND |
| 203 | M/45 | B-ALL | Diagnosis | HR | PETHEMA LAL-AR/2003 | ND | Rel-D | A | 1q X |  |  | Normal | F | N | ND |
| 204 | M/46 | B-ALL | Diagnosis | HR | UNKN | UNKN | UNKN | A | 19p |  |  | Normal | F | N | ND |
| 205 | F/53 | B-ALL | Diagnosis | HR | PETHEMA LAL-AR/93 | CR | CR-A | N |  |  |  | Normal | 46,XX[20] | N | ND |
| 206 | F/56 | B-ALL | Diagnosis | IR | PETHEMA LAL-RI/96 | CR | CR-A | A |  |  |  | Normal | 46,XX[12] | ND | N |
| 207 | M/60 | B-ALL | Diagnosis | HR | No therapy | No therapy | NT-ED | A | 21 | 7 9p 20 |  | Normal | 46,XY[21] | N | ND |
| 208 | F/63 | B-ALL | Diagnosis | HR | PETHEMA LAL-07OLD | CR | Rel-D | A |  |  |  | Normal | F | N | ND |
| 209 | M/66 | B-ALL | Diagnosis | IR | PETHEMA LAL-RI/96 | CR | CR-ED | A | X | 3 4 7 15 16 17 |  | Normal | 46,XY[28] | N | ND |
| 210 | F/71 | B-ALL | Diagnosis | HR | PETHEMA LAL-AR/93 | ND | ED | A |  |  |  | Normal | F | N | ND |
| 211 | F/72 | B-ALL | Diagnosis | HR | No therapy | No therapy | NT-ED | A | 1 6 14 18 21 | 7 9 15 16 17 20 |  | Normal | 46,XX[14] | ND | UNKN |
| 212 | F/74 | B-ALL | Diagnosis | HR | PETHEMA LAL-AR/93 | NA | ED | A |  | 20q |  | Normal | F | N | UNKN |
| 213 | F/79 | B-ALL | Diagnosis | HR | PETHEMA LAL-07OLD | CR | Rel-D | A |  |  |  | Normal | 46,XX[15] | N | N |
| 214 | F/79 | B-ALL | Diagnosis | HR | UNKN | ND | Rel-D | A | X |  |  | Normal | 46,XX[10] | N | UNKN |
| 215 | F/82 | B-ALL | Diagnosis | HR | No therapy | No therapy | NT-ED | A | 21 |  |  | Normal | 46,XX[18] | N | UNKN |
| 216 | M/1 | T-ALL | Diagnosis | LR | UNKN | CR | A | A | 10 |  |  | Normal | 46,XY[18] | ND | UNKN |
| 217 | M/3 | T-ALL | Diagnosis | LR | UNKN | CR | A | A |  |  |  | Normal | 46,XY[20] | ND | UNKN |
| 218 | F/5 | T-ALL | Diagnosis | AR | PETHEMA LAL-AR/2005 | CR | CR-A | A |  |  |  | Normal | 46,XX[10] | N | UNKN |
| 219 | M/5 | T-ALL | Diagnosis | LR | UNKN | CR | CR-A | A |  | 19p X |  | Normal | 46,XY[18] | ND | ND |
| 220 | M/6 | T-ALL | Diagnosis | LR | UNKN | CR | CR-A | A |  |  |  | Normal | 46,XY[16] | ND | UNKN |
| 221 | M/10 | T-ALL | Diagnosis | LR | SEHOP/LAL/SHOP-2005 | CR | CR-A | A |  |  |  | Normal | 46,XY[20] | N | UNKN |
| 222 | M/12 | T-ALL | Diagnosis | LR | UNKN | UNKN | Rel-A | A |  |  |  | Normal | 46,XY[5] | N | UNKN |
| 223 | M/14 | T-ALL | Diagnosis | IR | PETHEMA LAL-RI/96 | CR | Rel-D | A |  |  |  | Normal | ND | N | ND |
| 224 | F/14 | T-ALL | Diagnosis | HR | PETHEMA LAL-AR/2005 | CR | Rel-D | A |  |  |  | Normal | F | N | ND |
| 225 | M/14 | T-ALL | Diagnosis | LR | UNKN | CR | CR-A | A |  |  |  | Normal | 46,XY[20] | ND | ND |
| 226 | F/15 | T-ALL | Diagnosis | IR | UNKN | UNKN | UNKN | A | 21 | 9 |  | Normal | 46,XX[12] | ND | UNKN |
| 227 | M/17 | T-ALL | Diagnosis | HR | PETHEMA LAL-AR/93 | CR | CR-A | A |  |  |  | Normal | F | N | ND |
| 228 | M/17 | T-ALL | Diagnosis | HR | PETHEMA LAL-AR/2003 | NA | ED | A |  |  |  | Normal | 46,XY[15] | ND | ND |
| 229 | M/10 | T-ALL | Diagnosis | LR | UNKN | UNKN | UNKN | A | 9q** | 9p** |  | Normal | F | N | ND |
| 230 | M/15 | T-ALL | Diagnosis | HR | PETHEMA LAL-AR/93 | CR | CR-A | A |  |  |  | Normal | 46,XY[17] | N | ND |
| 231 | M/3 | T-ALL | Diagnosis | UNKN | UNKN | CR | CR-A | A | 16 17 19 20q 22 |  |  | Abnormal | 40-43,XY[10]/46,XY[4] | ND | ND |
| 232 | M/9 | T-ALL | Diagnosis | HR | UNKN | CR | Rel-SM-D | A |  | 11q |  | Abnormal | 92,XXYY[6]/46,XY[2] | BCR gain 98% ABL gain 98% | UNKN |
| 233 | M/12 | T-ALL | Diagnosis | LR | UNKN | CR | CR-A | A |  |  |  | Abnormal | 46,XY[18] | TEL gain-12% | FLT3-ITD Positive Mutation |
| 234 | M/13 | T-ALL | Diagnosis | HR | PETHEMA LAL-AR/2003 | Refr | Refr-ED | A |  | 4q X |  | Abnormal | 46,XY,der(2),t(9;12)(p13;q12)[18]/46,XY[2] | wcp9 -9 loss-90% | UNKN |
| 235 | M/15 | T-ALL | Diagnosis | IR | PETHEMA LAL-RI/96 | CR | CR-A | A |  |  |  | Abnormal | 46,XY,t(1;11)(p21;p14)[12]/46,XY[3] | wcp1 clonal 100% wcp11 clonal 100% | ND |
| 236 | F/16 | T-ALL | Diagnosis | IR | UNKN | PR | Refr-Rel-D | A | 4p 8 11 13 14 19 | Xp |  | Abnormal | 51-53,XX,+12,+13,+14,+20,+21 [15]/46,XX[3] | ND | UNKN |
| 237 | M/3 | T-ALL | Diagnosis | IR | UNKN | CR | CR-A | A |  |  |  | NE | F | ND | UNKN |
| 238 | F/3 | T-ALL | Diagnosis | IR | PETHEMA LAL-RI/96 | CR | Rel-CR-A | A |  | 19p |  | NE | F | ND | UNKN |
| 239 | M/5 | T-ALL | Diagnosis | IR | PETHEMA LAL-RI/96 | CR | CR-A | A | 13 |  |  | NE | F | ND | UNKN |
| 240 | M/7 | T-ALL | Diagnosis | IR | PETHEMA LAL-RI/96 | CR | CR-A | A |  | 9p |  | NE | F | ND | ND |
| 241 | M/11 | T-ALL | Diagnosis | HR | SEHOP/LAL/SHOP-99 | CR | Refr-Rel-D | A |  |  |  | NE | F | ND | UNKN |
| 242 | M/17 | T-ALL | Diagnosis | AR | PETHEMA LAL-AR/2003 | CR | CR-A | A | 1q | 9p X |  | NE | F | ND | UNKN |
| 243 | M/18 | T-ALL | Diagnosis | HR | PETHEMA LAL-AR/93 | CR | Refr-Rel-D | A |  |  |  | Normal | 46,XY[10] | N | ND |
| 244 | M/21 | T-ALL | Diagnosis | IR | UNKN | UNKN | Rel-CR-A | A |  |  |  | Normal | 46,XY[10] | ND | ND |
| 245 | M/24 | T-ALL | Diagnosis | HR | PETHEMA LAL-AR/2003 | CR | Rel-CR-A | A | X |  |  | Normal | 46,XY[19] | ND | UNKN |
| 246 | F/24 | T-ALL | Diagnosis | IR | Hoelzer protocol | CR | CR-A | A | 8 16p |  |  | Normal | 46,XX[12] | N | UNKN |
| 247 | M/28 | T-ALL | Diagnosis | HR | PETHEMA LAL-AR/93 | CR | Rel-D | A |  |  |  | Normal | 46,XY[10] | ND | ND |
| 248 | M/41 | T-ALL | Diagnosis | HR | PETHEMA LAL-AR/2003 | Refr | Refr-Rel-D | A |  |  |  | Normal | F | N | ND |
| 249 | F/43 | T-ALL | Diagnosis | HR | PETHEMA LAL-AR/2003 | CR | CR-A | A | 1q 21 |  |  | Normal | 46,XX[10] | N | N |
| 250 | F/46 | T-ALL | Diagnosis | HR | PETHEMA LAL-AR/2003 | CR | Refr-CR-D | A | 19 |  |  | Normal | 46,XX[7] | ND | ND |
| 251 | M/54 | T-ALL | Diagnosis | HR | PETHEMA LAL-AR 2011 | CR | CR-A | A |  |  | cth 14q | Normal | F | N | N |
| 252 | M/69 | T-ALL | Diagnosis | HR | PETHEMA LAL-07OLD | CR | Rel-D | A |  |  |  | Normal | 46,XY[20] | N | ND |
| 253 | F/70 | T-ALL | Diagnosis | HR | PETHEMA LAL-07FRAIL | CR | Rel-D | A | 8 |  |  | Normal | Not clonal: 48,XX,del(2)(p13),del(5)(q12q31),+2mar[1]/46,XX[19] | N | N |
| 254 | F/70 | T-ALL | Diagnosis | HR | PETHEMA LAL-07OLD | CR | Rel-CR-A | N |  |  |  | Normal | 46,XX[12] | N | ND |
| 255 | M/34 | T-ALL | Diagnosis | HR | PETHEMA LAL-AR/93 | CR | CR-A | A |  |  |  | Normal | 46,XY[10] | ND | ND |
| 256 | M/83 | T-ALL | Diagnosis | HR | UNKN | CR later on | Rel-D | A | 21 |  |  | Abnormal | 46,XY,t(11;12)(q13;p12)[4]/46,XY[11] | N | ND |
| 257 | F/38 | T-ALL | Diagnosis | HR | PETHEMA LAL-AR/2003 | CR later on | Refr-Rel-D | A | 17 | 19 |  | Abnormal | 47,XX,del(11)(q13q23),+21 [4]/46,XX [17] | N | N |
| 258 | F/58 | T-ALL | Diagnosis | HR | PETHEMA LAL-AR/2003 | CR | Refr-CR-A | A |  | 7p 12p |  | Abnormal | 46,XX,t(3;12)(p21;p12)[5]/46,XX[6] | N | ND |
| 259 | F/50 | T-ALL | Diagnosis | HR | PETHEMA LAL-AR/2003 | CR | Rel-CR-A | A |  |  |  | Abnormal | 46,XX,del(4)(p12)[12] | ND | ND |
| 260 | F/18 | T-ALL | Diagnosis | IR | UNKN | UNKN | UNKN | A |  |  |  | NE | F | ND | ND |
| 261 | M/28 | T-ALL | Diagnosis | HR | PETHEMA LAL-AR/2003 | CR | CR-D | A |  |  |  | NE | F | ND | ND |
| 262 | M/31 | T-ALL | Diagnosis | HR | PETHEMA LAL-AR/93 | CR | Rel-D | A |  |  |  | NE | F | ND | ND |
| 263 | M/33 | T-ALL | Diagnosis | HR | PETHEMA LAL-AR/2003 | CR | CR-A | A | 19 |  |  | NE | F | ND | ND |
| 264 | M/35 | T-ALL | Diagnosis | HR | PETHEMA LAL-AR/2003 | CR | CR-A | A |  |  |  | NE | F | ND | ND |
| 265 | F/55 | T-ALL | Diagnosis | HR | UNKN | Refr | Refr-ED | A |  |  |  | NE | F | ND | ND |
| ^1^ At the time of diagnosis | | | | | | | | | | | | | | | |
| **Abbreviations:** B-ALL, B-cell lineage; T-ALL, T lineage; LR, Low risk; IR, Intermediate risk; HR, high risk; TKI, tyrosine-kinase inhibitors; MRD, minimal residual disease; CR, complete remission; ER, early relapse; ED, early death; Refr, refractory; N, aCGH Normal; A, aCGH Altered; Cth, chromothripsis; Hyper (>50), High hyperdiploidy (>50 chromosomes); Hyper (47-50), low hyperdiploidy (47-50 chromosomes), Hypo (<44), low hypodiploid (<44 chromosomes); Hypo (44-45), high hypodiploidy (44-45 chromosomes); NE, not evaluable; ND, not done; UNKN, Unknown; F, failed/karyotype not successful or metaphases were not acquired; FISH, fluorescence in situ hybridization; NA: not applicable. *Consistent with an isochromosome 7q, i(7q). **Consistent with an isochromosome 9q, i(9q).***Consistent with an isochromosome 17q, i(17q). ****Consistent with pseudodiploid karyotype with unbalanced translocation involving gain of 7q and loss of 7p. | | | | | | | | | | | | | | | |

**Table B. Pairwise comparisons of CNAs according to immunophenotypic, age and cytogenetic subgroups of ALL patients**

| Pairwise comparison parameter | Pairwise comparison | Chromosomal sites | B-ALL children (n=115) | T-ALL children (n=27) | *p* | Candidate genes | Type of CNA |
| --- | --- | --- | --- | --- | --- | --- | --- |
|  |  |  | % | % |  |  |  |
| Immunophenotype | Immunophenotype in ALL children | 12p13.2 | **27.5** | 0 | 0.002 | *ETV6* | CN Loss |
|  |  | 18q12-q21 | **14.7** | 0 | 0.043 | *SMAD2* | CN Gain |
|  |  | 21 | **27.8** | 3.7 | 0.005 | *RUNX1, ERG* | CN Gain |
|  |  | X | **13.9** | 0 | 0.042 | *DMD, STAG2, PHF6* | CN Gain |
|  |  | 1q21 | 8.6 | **29.6** | 0.007 |  | CN Gain |
|  |  | 2q14.1 | 0.9 | **11.1** | 0.022 |  | CN Loss |
|  |  | 3q26.1-q26.2 | 0.9 | **11.1** | 0.022 |  | CN Loss |
|  |  | 4q21 | 0.0 | **11.1** | 0.006 |  | CN Loss |
|  |  | 5q32-q35.3 | 0.0 | **11.1** | 0.006 |  | CN Loss |
|  |  | 6q13 | 1.7 | **14.8** | 0.012 |  | CN Loss |
|  |  | 8q24.3 | 1.7 | **14.8** | 0.012 |  | CN Gain |
|  |  | 9p21.3 | 33.0 | **70.4** | <0.0001 | *MLLT3, PTPLAD2, IFN, MTAP, CDKN2A/B* and *DMRTA1* | CN Loss |
|  |  | 11q14.3 | 0.9 | **14.8** | 0.005 |  | CN Gain |
|  |  | 13q | 0.0 | **7.4** | 0.035 | *PDS5B, RB1, TRIM13* | CN Gain |
|  |  | 17q21.31 | 15.6 | **37** | 0.028 |  | CN Gain |
|  |  | 19p13.2 | 5.2 | **22.0** | 0.011 | *DNM2, EPOR* | CN Loss |
|  |  | 20q13.12 | 0.0 | **7.4** | 0.035 |  | CN Gain |
|  | Pairwise comparison | Chromosomal sites | B-ALL adults (n=100) | T-ALL adults (n=23) | *p* | Candidate genes | Type of CNA |
|  |  |  | % | % |  |  |  |
|  | Immunophenotype in ALL adults | 7p12.2 | **47.0** | 8.6 | 0.001 | *IKZF1* | CN Loss |
|  |  | 12q21.33 | **21.0** | 0.0 | 0.012 | *BTG1* | CN Loss |
|  |  | 5q33.3 | **23.0** | 0.0 | 0.007 | *EBF1* | CN Loss |
|  |  | 21q21.3 | **16.0** | 0.0 | 0.041 |  | CN Gain |
|  |  | 2p16 | 1.0 | **13.0** | 0.02 |  | CN Loss |
|  |  | 5q23.2-q23.3 | 20.0 | **13.0** | 0.045 |  | CN Loss |
|  |  | 5q33.1-q33.2 | 2.0 | **13.0** | 0.045 |  | CN Loss |
|  |  | 10p15 | 3.0 | **17.0** | 0.023 |  | CN Loss |
|  |  | 10q22.1 | 2.0 | **13.0** | 0.045 |  | CN Loss |
|  |  | 10q26.3 | 6.0 | **21.7** | 0.032 |  | CN Loss |
|  |  | 11q12.2-q13.2 | 1.0 | **13.0** | 0.02 |  | CN Gain |
|  |  | 12p13.33 | 2.0 | **13.0** | 0.045 |  | CN Loss |
|  |  | 14q31.2-q32.11 | 0.0 | **13.0** | 0.006 |  | CN Loss |
|  |  | 17q21.33 | 4.0 | **26.0** | 0.003 |  | CN Gain |
|  |  | 17q25.3 | 3.0 | **26.0** | 0.001 |  | CN Gain |
|  |  | 19q13.32-q13.33 | 7.0 | **21.7** | 0.047 |  | CN Gain |
| Pairwise comparison parameter | Pairwise comparison | Chromosomal sites | B-ALL children (n=127) | B-ALL adults (n=123) | p | Candidate genes | Type of CNA |
|  |  |  | % | % |  |  |  |
| Age group | Age group B-ALL patients | 12p13.2 | **28.0** | 7.0 | <0.0001 | *ETV6* | CN Loss |
|  |  | 21 | **29.5** | 12.0 | 0.002 | *RUNX1, ERG* | CN Gain |
|  |  | 1q32.1 | 4.3 | **12.0** | 0.045 |  | CN Gain |
|  |  | 3p14 | 0.9 | **10.0** | 0.003 |  | CN Loss |
|  |  | 5q33.3 | 11.3 | **23.0** | 0.022 | *EBF1* | CN Loss |
|  |  | 7p12.2 | 18.0 | **47.0** | <0.0001 | *IKZF1* | CN Loss |
|  |  | 9p21.3 | 33.0 | **47.0** | 0.037 | *CDKN2A/B, MTAP* | CN Loss |
|  |  | 12q21.33 | 9.6 | **21.0** | 0.022 | *BTG1* | CN Loss |
|  | Pairwise comparison | Chromosomal sites | T-ALL children (n=27) | T-ALL adults (n=23) | p | Candidate genes | Type of CNA |
|  |  |  | % | % |  |  |  |
|  | Age group T-ALL patients | 1q21 | **29.6** | 4.3 | 0.028 |  | CN Gain |
|  |  | 4q21.22 | **22.2** | 0.0 | 0.025 |  | CN Loss |
|  |  | 9p21.3 | **37.0** | 8.7 | 0.024 |  | CN Loss |
|  |  | 9p21.2-p21.1 | **29.6** | 4.3 | 0.028 |  | CN Loss |
|  |  | 7p21.3 | 0.0 | **21.7** | 0.016 |  | CN Loss |
|  | Pairwise comparison | Chromosomal sites | E/R (n=19) | non-E/R (n=72) | p | Candidate genes | Type of CNA |
|  |  |  | % | % |  |  |  |
| Cytogenetic subgroup ^1^ | Cytogenetic subgroup in B-ALL children | 1q21.3-q22 | **21.0** | 4.1 | 0.032 |  | CN Gain |
|  |  | 6p21.33-p21.32 | **26.3** | 6.9 | 0.03 |  | CN Gain |
|  |  | 6p25 | **31.5** | 9.7 | 0.026 |  | CN Gain |
|  |  | 10q21 | **10.5** | 0.0 | 0.042 |  | CN Loss |
|  |  | 12p13.2 | **68.4** | 15.0 | <0.0001 | *ETV6* | CN Loss |
|  |  | 13q21.31-q21.33 | **21.0** | 2.7 | 0.016 |  | CN Loss |
|  |  | 13q31.1-q31.3 | **21.0** | 2.7 | 0.016 |  | CN Loss |
|  |  | 16p13-p12 | **21.0** | 4.1 | 0.03 |  | CN Gain |
|  |  | 17q21.31-q21.33 | **26.3** | 5.6 | 0.017 |  | CN Gain |
|  |  | 21 | **37.0** | 15.0 | 0.049 | *RUNX1* | CN Gain |
|  |  | 7p12.2 | 0.0 | **22.2** | 0.022 | *IKZF1* | CN Loss |
|  | Pairwise comparison | Chromosomal sites | Ph+ (n=33) | Ph- (n=67) | p | Candidate genes | Type of CNA |
|  |  |  | % | % |  |  |  |
|  | Cytogenetic subgroup in B-ALL adults | 2p15 | **9.0** | 0.0 | 0.034 |  | CN Gain |
|  |  | 2q21 | **9.0** | 0.0 | 0.034 |  | CN Gain |
|  |  | 7p12.2 | **72.7** | 34.3 | <0.0001 | *IKZF1* | CN Loss |
|  |  | 1q | 0.0 | **13.4** | 0.028 |  | CN Gain |
|  |  | 1p13.3-p12 | 0.0 | **13.4** | 0.028 |  | CN Gain |
|  |  | 3p14 | 0.0 | **13.4** | 0.028 |  | CN Loss |
|  |  | 19p13.2 | 0.0 | **16.4** | 0.014 | *E2A, DNM2, JAK3* | CN Gain |
|  |  | 21 | 3.0 | **17.9** | 0.037 | *RUNX1, ERG* | CN Gain |
|  |  |  |  |  |  |  |  |
| ^1^In the pairwise cytogenetic subgroups analysis, the pediatric B-ALL patients *ETV6-RUNX1* (*E/R*, n=19) and non-*ETV6-RUNX1* (non-*E/R*, n=72, 24 patients with hyperdiploid cytogenetics in this group were excluded) were grouped. The adult ALL samples were separated into *BCR-ABL1* (Ph+, n=33) and non-*BCR-ABL1* (Ph-, n=67) groups. **Abbreviations**: E/R, *ETV6-RUNX1*; non-E/R, non- *ETV6-RUNX1*; Ph+, *BCR-ABL*, Ph-, *BCR-ABL.* | | | | | | | |
|  |  |  |  |  |  |  |  |

**Table C. Regions of significant recurrent amplification and deletion in the whole cohort of children with ALL (n=142) (q<0.05)**

| **Unique name** | **Cytoband** | **Wide peak limits** | **Peak boundaries (Mb)** | **q** | **Candidate target gene(s)** | **Alterations referenced in other studies** |
| --- | --- | --- | --- | --- | --- | --- |
| Amplification Peak 1 | 1p36.33 | chr1:1-1069257 | 1069256 | 3.18E-06 |  |  |
| Amplification Peak 2 | 1p36.11 | chr1:25405392-25640963 | 235571 | 5.74E-06 |  |  |
| Amplification Peak 3 | 1p34.3 | chr1:37152758-37398602 | 245844 | 1.80E-04 |  |  |
| Amplification Peak 4 | 1p12 | chr1:120306172-120407161 | 100989 | 8.34E-11 |  |  |
| Amplification Peak 5 | 1q21.1 | chr1:146905298-147304143 | 398845 | 4.01E-14 |  |  |
| Amplification Peak 6 | 1q32.1 | chr1:203130267-203227787 | 97520 | 4.93E-12 |  |  |
| Amplification Peak 7 | 2p11.2 | chr2:85729984-85973845 | 243861 | 0.00011869 |  |  |
| Amplification Peak 8 | 2p11.2 | chr2:87405248-87764945 | 359697 | 8.25E-07 |  |  |
| Amplification Peak 9 | 2p11.2 | chr2:88834969-89339118 | 504149 | 5.97E-04 |  |  |
| Amplification Peak 10 | 2q11.2 | chr2:95908727-96058869 | 150142 | 5.63E-08 |  |  |
| Amplification Peak 11 | 2q13 | chr2:113586821-113782979 | 196158 | 1.26E-03 |  |  |
| Amplification Peak 12 | 3p25.1 | chr3:13384272-13853277 | 469005 | 6.63E-09 |  |  |
| Amplification Peak 13 | 3q21.1 | chr3:124512683-124656479 | 143796 | 5.22E-03 |  |  |
| Amplification Peak 14 | 4p16.1 | chr4:9713139-9906598 | 193459 | 0.0007674 |  |  |
| Amplification Peak 15 | 4q34.3 | chr4:180069922-180567652 | 497730 | 1.43E-03 |  |  |
| Amplification Peak 16 | 5p15.33 | chr5:1-929826 | 929825 | 1.80E-04 |  |  |
| Amplification Peak 17 | 5q13.2 | chr5:69793739-70188314 | 394575 | 1.69E-11 |  |  |
| Amplification Peak 18 | 5q31.1 | chr5:131278970-131714645 | 435675 | 1.15E-05 |  |  |
| Amplification Peak 19 | 6p25.3 | chr6:1-288976 | 288975 | 2.34E-10 |  | [[4](#_ENREF_4)] |
| Amplification Peak 20 | 6p21.1 | chr6:40900320-41122077 | 221757 | 3.18E-06 |  |  |
| Amplification Peak 21 | 6q15 | chr6:91624605-92003556 | 378951 | 0.0005263 |  |  |
| Amplification Peak 22 | 7p22.3 | chr7:1-649546 | 649545 | 3.54E-04 |  |  |
| Amplification Peak 23 | 7p15.1 | chr7:30653704-30992557 | 338853 | 9.11E-05 |  |  |
| Amplification Peak 24 | 7q11.23 | chr7:73815282-73936338 | 121056 | 1.07E-08 |  |  |
| Amplification Peak 25 | 7q22.1 | chr7:101852347-102125707 | 273360 | 4.79E-24 |  |  |
| Amplification Peak 26 | 7q34 | chr7:141642831-142018083 | 375252 | 4.02E-05 |  |  |
| Amplification Peak 27 | 7q35 | chr7:143528315-143708603 | 180288 | 2.48E-24 |  |  |
| Amplification Peak 28 | 8p23.1 | chr8:6736494-7199608 | 463114 | 1.4884E-06 |  |  |
| Amplification Peak 29 | 8p23.1 | chr8:7228102-7759993 | 531891 | 4.61E-40 |  |  |
| Amplification Peak 30 | 8q24.23 | chr8:139620139-139742036 | 121897 | 1.80E-04 |  | [[5](#_ENREF_5)] |
| Amplification Peak 31 | 9p13.1 | chr9:38897716-38987239 | 89523 | 1.51E-22 |  |  |
| Amplification Peak 32 | 9p11.2 | chr9:45182226-45997389 | 815163 | 1.15E-20 |  |  |
| Amplification Peak 33 | 9q12 | chr9:65625053-65719707 | 94654 | 1.65E-24 |  |  |
| Amplification Peak 34 | 9q12-q13 | chr9:68227271-70145497 | 1918226 | 2.55E-15 |  |  |
| Amplification Peak 35 | 9q22.1 | chr9:90611836-90878920 | 267084 | 0.000078775 |  |  |
| Amplification Peak 36 | 10q22.3 | chr10:81024277-81215128 | 190851 | 4.70E-09 |  |  |
| Amplification Peak 37 | 10q26.13 | chr10:125713327-125792184 | 78857 | 5.58E-10 |  |  |
| Amplification Peak 38 | 11p15.1 | chr11:17460839-17684391 | 223552 | 4.63E-04 |  |  |
| Amplification Peak 39 | 11q12.2 | chr11:60300942-60464995 | 164053 | 2.28E-05 |  |  |
| Amplification Peak 40 | 11q14.3 | chr11:88164034-89562653 | 1398619 | 6.73E-04 |  |  |
| Amplification Peak 41 | 12p13.31 | chr12:6893638-7052627 | 158989 | 2.37E-04 |  |  |
| Amplification Peak 42 | 12q13.13 | chr12:50841555-51486371 | 644816 | 0.000015141 |  |  |
| Amplification Peak 43 | 14q11.2 | chr14:21848303-21941919 | 93616 | 8.40E-34 |  |  |
| Amplification Peak 44 | 14q32.33 | chr14:104206007-104764059 | 558052 | 1.42E-10 |  |  |
| Amplification Peak 45 | 15q11.2 | chr15:18964148-19647347 | 683199 | 7.89E-31 |  |  |
| Amplification Peak 46 | 15q24.1 | chr15:72212081-72532025 | 319944 | 1.19E-04 |  |  |
| Amplification Peak 47 | 16p12.1 | chr16:22491335-22628366 | 137031 | 2.77E-07 |  |  |
| Amplification Peak 48 | 16p11.2 | chr16:31950858-33366588 | 1415730 | 3.84E-22 |  |  |
| Amplification Peak 49 | 16q22.2 | chr16:69405876-69640686 | 234810 | 7.6565E-06 |  |  |
| Amplification Peak 50 | 17p13.1 | chr17:7648062-7782605 | 134543 | 3.72E-03 |  |  |
| Amplification Peak 51 | 17p11.2 | chr17:18881246-18952836 | 71590 | 6.88E-05 |  |  |
| Amplification Peak 52 | 17q21.31 | chr17:41720894-41854797 | 133903 | 2.85E-20 |  |  |
| Amplification Peak 53 | 17q21.33 | chr17:45218233-46123361 | 905128 | 1.03E-02 |  |  |
| Amplification Peak 54 | 18p11.21 | chr18:14020876-14370055 | 349179 | 2.47E-02 |  |  |
| Amplification Peak 55 | 18q21.1 | chr18:42363427-42516716 | 153289 | 5.97E-04 |  |  |
| Amplification Peak 56 | 20q13.31 | chr20:54999411-55202985 | 203574 | 0.033558 |  |  |
| Amplification Peak 57 | 21q11.2 | chr21:1-14322834 | 14322833 | 9.76E-04 |  |  |
| Amplification Peak 58 | 21q22.3 | chr21:45037375-45396415 | 359040 | 2.07E-04 |  |  |
| Amplification Peak 59 | 22q11.21 | chr22:18883524-19202917 | 319393 | 1.03E-02 |  |  |
| Amplification Peak 60 | Xp22.33 | chrX:3727161-3928515 | 201354 | 1.34E-19 |  |  |
| Amplification Peak 61 | Xp21.1 | chrX:36644121-36711384 | 67263 | 3.32E-10 |  |  |
| Amplification Peak 62 | Xp11.23 | chrX:48424305-48481125 | 56820 | 1.07E-08 |  |  |
| Amplification Peak 63 | Xp11.22 | chrX:52520031-52580680 | 60649 | 1.9761E-11 |  |  |
| Amplification Peak 64 | Xq26.3 | chrX:134667634-134767793 | 100159 | 7.55E-24 |  |  |
| Amplification Peak 65 | Xq28 | chrX:151741367-151984941 | 243574 | 8.98E-10 |  |  |
| Amplification Peak 66 | Xq28 | chrX:153012612-153202329 | 189717 | 1.04E-27 |  |  |
| Deletion Peak 1 | 1p36.33 | chr1:580412-1099611 | 519199 | 4.63E-05 |  |  |
| Deletion Peak 2 | 1p36.22 | chr1:9886550-10636708 | 750158 | 4.67E-04 |  |  |
| Deletion Peak 3 | 1p36.11 | chr1:25161288-25772739 | 611451 | 1.78E-08 |  |  |
| Deletion Peak 4 | 1q12-q21.1 | chr1:120407163-143108109 | 22700946 | 1.56E-03 |  |  |
| Deletion Peak 5 | 1q21.1 | chr1:145938613-147852780 | 1914167 | 8.61E-08 |  |  |
| Deletion Peak 6 | 1q22 | chr1:153508933-153923044 | 414111 | 1.31E-05 |  |  |
| Deletion Peak 7 | 2p14-p13.3 | chr2:70038109-70531061 | 492952 | 0.0061498 |  |  |
| Deletion Peak 8 | 2p11.2 | chr2:86919832-88108759 | 1188927 | 1.76E-04 |  |  |
| Deletion Peak 9 | 2q11.2-q11.1 | chr2:88677247-95006650 | 6329403 | 1.38E-03 |  |  |
| Deletion Peak 10 | 2q13 | chr2:111639109-112392452 | 753343 | 3.26E-04 |  |  |
| Deletion Peak 11 | 3p21.31 | chr3:48982985-50458040 | 1475055 | 9.83E-04 |  | [[5](#_ENREF_5)] |
| Deletion Peak 12 | 3q26.32 | chr3:176961046-179762212 | 2801166 | 4.47E-06 | *TBL1XR1* | [[5](#_ENREF_5)] |
| Deletion Peak 13 | 4p14 | chr4:39168517-40039471 | 870954 | 1.73E-03 |  |  |
| Deletion Peak 14 | 4q35.2 | chr4:187879106-191128000 | 3248894 | 0.011207 |  |  |
| Deletion Peak 15 | 5p15.33 | chr5:1-929826 | 929825 | 1.73E-03 |  |  |
| Deletion Peak 16 | 5p13.3 | chr5:31570157-32421460 | 851303 | 6.57E-03 |  |  |
| Deletion Peak 17 | 5q13.2 | chr5:68897383-70841211 | 1943828 | 8.08E-18 |  |  |
| Deletion Peak 18 | 5q33.2 | chr5:153833880-154219759 | 385879 | 7.39E-05 |  | [[5](#_ENREF_5)] |
| Deletion Peak 19 | 6p25.3 | chr6:1-340019 | 340018 | 4.21E-06 |  |  |
| Deletion Peak 20 | 6p21.31 | chr6:34469176-34963211 | 494035 | 5.12E-03 |  |  |
| Deletion Peak 21 | 6q13 | chr6:73961018-74362831 | 401813 | 0.000077703 |  |  |
| Deletion Peak 22 | 6q21-q22.1 | chr6:90661774-116561558 | 25899784 | 5.06E-03 | *EPHA7, FYN* | [[4](#_ENREF_4), [6](#_ENREF_6)] |
| Deletion Peak 23 | 7p22.3 | chr7:767316-1462916 | 695600 | 7.26E-07 |  |  |
| Deletion Peak 24 | 7p12.2 | chr7:50016384-50491610 | 475226 | 5.31E-05 | *IKZF1* | [[4](#_ENREF_4), [5](#_ENREF_5)] |
| Deletion Peak 25 | 7p11.2 | chr7:55173046-57214733 | 2041687 | 8.61E-04 |  |  |
| Deletion Peak 26 | 7q22.1 | chr7:101816673-102245823 | 429150 | 3.32E-07 |  |  |
| Deletion Peak 27 | 7q34 | chr7:141583326-142286761 | 703435 | 2.38E-13 |  | [[5](#_ENREF_5)] |
| Deletion Peak 28 | 7q35 | chr7:143561053-143738775 | 177722 | 2.2338E-20 |  |  |
| Deletion Peak 29 | 8p23.1 | chr8:6864662-8228003 | 1363341 | 3.42E-36 |  |  |
| Deletion Peak 30 | 8q21.13 | chr8:81223792-81718685 | 494893 | 3.96E-05 |  |  |
| Deletion Peak 31 | 9p21.3 | chr9:21494039-22007872 | 513833 | 1.06E-67 | *CDKN2A/B, MTAP* | [[4-9](#_ENREF_4)] |
| Deletion Peak 32 | 9p13.1-p12 | chr9:38409289-40748747 | 2339458 | 1.38E-10 |  | [[5](#_ENREF_5), [6](#_ENREF_6)] |
| Deletion Peak 33 | 9q12 | chr9:43013849-67632305 | 24618456 | 5.52E-11 |  |  |
| Deletion Peak 34 | 9q34.11 | chr9:130799172-132004095 | 1204923 | 4.04E-03 |  |  |
| Deletion Peak 35 | 10p12.31 | chr10:21468576-21871051 | 402475 | 0.0092374 |  |  |
| Deletion Peak 36 | 10p11.21 | chr10:35949172-38165551 | 2216379 | 2.81E-02 |  |  |
| Deletion Peak 37 | 10q11.22 | chr10:46418280-47224204 | 805924 | 5.68E-15 |  |  |
| Deletion Peak 38 | 11p11.2 | chr11:47396598-47973063 | 576465 | 4.03E-04 |  |  |
| Deletion Peak 39 | 11q11.12-q11 | chr11:48352876-54878808 | 6525932 | 1.39E-03 |  |  |
| Deletion Peak 40 | 11q14.3 | chr11:87678990-91751828 | 4072838 | 1.03E-02 |  |  |
| Deletion Peak 41 | 11q23.3 | chr11:116435107-116667558 | 232451 | 4.04E-03 |  | [[4](#_ENREF_4), [6](#_ENREF_6)] |
| Deletion Peak 42 | 12p13.33 | chr12:864084-1550190 | 686106 | 1.3947E-07 |  |  |
| Deletion Peak 43 | 12p13.2 | chr12:11240452-12193068 | 952616 | 1.69E-16 | *ETV6* | [[4](#_ENREF_4), [5](#_ENREF_5), [8](#_ENREF_8)] |
| Deletion Peak 44 | 12p13.1 | chr12:12571973-13139493 | 567520 | 1.01E-16 | *CDKN1B* |  |
| Deletion Peak 45 | 12q21.33-q22 | chr12:90094910-91349761 | 1254851 | 7.70E-04 | *BTG1* |  |
| Deletion Peak 46 | 12q24.31 | chr12:120613852-121332676 | 718824 | 2.96E-04 |  |  |
| Deletion Peak 47 | 12q24.33 | chr12:130166382-131795104 | 1628722 | 5.22E-04 |  |  |
| Deletion Peak 48 | 13q14.3 | chr13:49259207-50197149 | 937942 | 2.10E-03 | *TRIM13* | [[4](#_ENREF_4), [5](#_ENREF_5)] |
| Deletion Peak 49 | 13q34 | chr13:113355489-114142980 | 787491 | 0.00059908 |  |  |
| Deletion Peak 50 | 14q13.2 | chr14:34143096-34842445 | 699349 | 1.35E-04 |  |  |
| Deletion Peak 51 | 14q32.33 | chr14:104960404-106368585 | 1408181 | 2.56E-09 | *MTA1* |  |
| Deletion Peak 52 | 15q11.2 | chr15:1-19358754 | 19358753 | 3.60E-17 |  |  |
| Deletion Peak 53 | 15q13.2 | chr15:27889901-28759043 | 869142 | 1.49E-04 | *TJP1 (ZO-1)* |  |
| Deletion Peak 54 | 16p13.3 | chr16:949866-1610547 | 660681 | 3.53E-05 |  |  |
| Deletion Peak 55 | 16p13.11 | chr16:14885722-15040092 | 154370 | 1.99E-05 |  |  |
| Deletion Peak 56 | 16q11.2 | chr16:31738698-45186808 | 13448110 | 0.00020154 |  |  |
| Deletion Peak 57 | 16q22.1 | chr16:67278758-67808331 | 529573 | 2.22E-06 |  | [[4](#_ENREF_4)] |
| Deletion Peak 58 | 17p13.1 | chr17:6928001-7397956 | 469955 | 2.10E-03 |  | [[4](#_ENREF_4), [5](#_ENREF_5)] |
| Deletion Peak 59 | 17p11.2 | chr17:18623862-19091255 | 467393 | 7.77E-05 |  | [[4](#_ENREF_4), [5](#_ENREF_5)] |
| Deletion Peak 60 | 17q21.31 | chr17:41604760-41854797 | 250037 | 7.38E-37 |  |  |
| Deletion Peak 61 | 18q11.2 | chr18:16931030-18008789 | 1077759 | 3.35E-04 |  |  |
| Deletion Peak 62 | 19p13.3 | chr19:1009412-1780982 | 771570 | 6.54E-06 | *TCF3* |  |
| Deletion Peak 63 | 19q13.2 | chr19:42949242-43680694 | 731452 | 0.011207 |  |  |
| Deletion Peak 64 | 20q13.12 | chr20:45215850-45776192 | 560342 | 4.33E-06 |  |  |
| Deletion Peak 65 | 20q13.33 | chr20:61021639-62435964 | 1414325 | 2.50E-04 |  |  |
| Deletion Peak 66 | 21q11.2 | chr21:1-14098327 | 14098326 | 2.31E-03 |  |  |
| Deletion Peak 67 | 22q11.21 | chr22:19660347-20447121 | 786774 | 2.79E-03 | *MAPK1* |  |
| Deletion Peak 68 | 22q11.22 | chr22:20926300-21558295 | 631995 | 3.13E-05 | *VPREB1* |  |
| Deletion Peak 69 | 22q12.1 | chr22:26644459-27818005 | 1173546 | 1.90E-03 |  |  |
| Deletion Peak 70 | Xp22.33 | chrX:1360605-1680989 | 320384 | 0.000022682 | *ASMTL, CRLF2, SLC25A6, IL3RA, CSF2RA, P2RY8* |  |
| Deletion Peak 71 | Xp11.22 | chrX:52322502-52745817 | 423315 | 2.20E-09 |  |  |
| Deletion Peak 72 | Xq24 | chrX:118587105-119029760 | 442655 | 5.73E-06 |  |  |
| Deletion Peak 73 | Xq26.3 | chrX:134537499-134889218 | 351719 | 1.44E-06 |  |  |
| Deletion Peak 74 | Xq28 | chrX:153010365-153299880 | 289515 | 1.40E-19 |  |  |

**Table D. Regions of significant recurrent amplification and deletion in the whole cohort of adults with ALL (n=123) (q<0.05)**

| **Unique name** | **Cytoband** | **Wide peak limits** | **Peak boundaries (Mb)** | **q** | **Candidate target gene(s)** | **Alterations referenced in other studies** |
| --- | --- | --- | --- | --- | --- | --- |
| Amplification Peak 1 | 1p36.22 | chr1:11375397-11758027 | 382630 | 2.32E-08 |  |  |
| Amplification Peak 2 | 1p34.2 | chr1:41843832-42073927 | 230095 | 1.74E-05 |  |  |
| Amplification Peak 3 | 1p12 | chr1:120280136-120436508 | 156372 | 1.32E-07 |  |  |
| Amplification Peak 4 | 1q21.1 | chr1:146817574-147575336 | 757762 | 7.21E-10 |  |  |
| Amplification Peak 5 | 1q22 | chr1:153161633-153513199 | 351566 | 8.92E-10 |  |  |
| Amplification Peak 6 | 1q32.1 | chr1:202846333-203332179 | 485846 | 7.49E-12 |  |  |
| Amplification Peak 7 | 2p13.3 | chr2:71609327-72110343 | 501016 | 0.00032751 |  |  |
| Amplification Peak 8 | 2p11.2 | chr2:85751968-85881772 | 129804 | 6.25E-08 |  |  |
| Amplification Peak 9 | 2p11.2 | chr2:88834969-91219115 | 2384146 | 0.0025757 |  |  |
| Amplification Peak 10 | 2q13 | chr2:113736755-113797433 | 60678 | 1.04E-05 |  |  |
| Amplification Peak 11 | 2q37.3 | chr2:242460162-242951149 | 490987 | 5.11E-06 |  |  |
| Amplification Peak 12 | 3p25.1 | chr3:13377482-13781202 | 403720 | 1.55E-08 |  |  |
| Amplification Peak 13 | 3q21.1 | chr3:124568100-124657216 | 89116 | 0.00043735 |  |  |
| Amplification Peak 14 | 3q27.1 | chr3:185242075-185626705 | 384630 | 0.0029865 |  |  |
| Amplification Peak 15 | 4p16.1 | chr4:9713139-10018377 | 305238 | 4.12E-07 |  |  |
| Amplification Peak 16 | 4q31.3 | chr4:152946990-153284636 | 337646 | 0.012433 |  |  |
| Amplification Peak 17 | 4q34.3 | chr4:179891928-180567652 | 675724 | 0.00069217 |  |  |
| Amplification Peak 18 | 5p15.33 | chr5:1-929826 | 929825 | 0.00060261 |  |  |
| Amplification Peak 19 | 5q13.2 | chr5:69455975-70218333 | 762358 | 4.75E-10 |  |  |
| Amplification Peak 20 | 5q31.1 | chr5:131377220-131716238 | 339018 | 3.50E-07 |  |  |
| Amplification Peak 21 | 5q31.3 | chr5:138838226-139165107 | 326881 | 3.46E-08 |  |  |
| Amplification Peak 22 | 6p25.3 | chr6:1-308940 | 308939 | 4.75E-05 |  |  |
| Amplification Peak 23 | 6p21.33 | chr6:30711557-30855906 | 144349 | 1.24E-05 |  |  |
| Amplification Peak 24 | 6p21.1 | chr6:40778102-41069700 | 291598 | 2.00E-09 |  |  |
| Amplification Peak 25 | 6q21 | chr6:112384981-112577722 | 192741 | 2.43E-05 |  |  |
| Amplification Peak 26 | 6q27 | chr6:168106233-168341186 | 234953 | 5.61E-05 |  |  |
| Amplification Peak 27 | 7p22.3 | chr7:1-325772 | 325771 | 5.61E-05 |  |  |
| Amplification Peak 28 | 7p15.1 | chr7:30638462-30992557 | 354095 | 0.00080061 |  |  |
| Amplification Peak 29 | 7p13 | chr7:43943812-44401715 | 457903 | 6.55E-05 |  |  |
| Amplification Peak 30 | 7q22.1 | chr7:101852347-102096490 | 244143 | 6.54E-13 |  |  |
| Amplification Peak 31 | 7q35 | chr7:143529099-143705112 | 176013 | 2.80E-26 |  |  |
| Amplification Peak 32 | 8p23.1 | chr8:6749071-6861477 | 112406 | 1.32E-07 |  |  |
| Amplification Peak 33 | 8p23.1 | chr8:7217189-7817360 | 600171 | 6.56E-18 |  |  |
| Amplification Peak 34 | 8q24.3 | chr8:140564453-140933010 | 368557 | 2.00E-09 |  | [[10](#_ENREF_10)] |
| Amplification Peak 35 | 9p13.1 | chr9:38844930-38987239 | 142309 | 5.60E-17 |  |  |
| Amplification Peak 36 | 9p11.2 | chr9:43533913-43814520 | 280607 | 2.32E-08 |  |  |
| Amplification Peak 37 | 9q12 | chr9:65495416-65720561 | 225145 | 3.92E-10 |  |  |
| Amplification Peak 38 | 9q12 | chr9:68170618-70202699 | 2032081 | 2.00E-09 |  |  |
| Amplification Peak 39 | 9q22.1 | chr9:90610703-90825987 | 215284 | 0.0012444 |  |  |
| Amplification Peak 40 | 9q34.13 | chr9:132812064-133093759 | 281695 | 4.75E-05 |  |  |
| Amplification Peak 41 | 10p15.3 | chr10:1-569211 | 569210 | 0.035625 |  |  |
| Amplification Peak 42 | 10q11.22 | chr10:46125140-46833453 | 708313 | 2.86E-07 |  |  |
| Amplification Peak 43 | 10q22.3 | chr10:80944578-81192542 | 247964 | 2.50E-06 |  |  |
| Amplification Peak 44 | 10q26.13 | chr10:125794468-125835554 | 41086 | 3.18E-10 |  |  |
| Amplification Peak 45 | 11p15.1 | chr11:17460839-17667313 | 206474 | 1.02E-06 |  |  |
| Amplification Peak 46 | 11p11.2 | chr11:44820171-45194106 | 373935 | 0.00024143 |  |  |
| Amplification Peak 47 | 11q12.2 | chr11:60262557-60464995 | 202438 | 1.02E-06 |  |  |
| Amplification Peak 48 | 11q25 | chr11:133846180-134265155 | 418975 | 2.86E-07 |  |  |
| Amplification Peak 49 | 12p13.31 | chr12:5971066-7253635 | 1282569 | 0.00012515 |  |  |
| Amplification Peak 50 | 12q13.3 | chr12:55704300-55759222 | 54922 | 0.00017317 |  |  |
| Amplification Peak 51 | 12q24.33 | chr12:131220442-131942574 | 722132 | 2.05E-05 |  |  |
| Amplification Peak 52 | 13q14.3 | chr13:51253098-51372668 | 119570 | 0.0014461 |  |  |
| Amplification Peak 53 | 13q34 | chr13:110988834-111751354 | 762520 | 0.0034228 |  |  |
| Amplification Peak 54 | 14q11.2 | chr14:21740367-21941919 | 201552 | 1.10E-38 |  |  |
| Amplification Peak 55 | 14q32.33 | chr14:104789286-105053955 | 264669 | 8.70E-06 | *MTA1* |  |
| Amplification Peak 56 | 15q11.2 | chr15:19091070-19501824 | 410754 | 2.58E-28 |  |  |
| Amplification Peak 57 | 15q24.3 -q25.1 | chr15:75805187-76108174 | 302987 | 4.12E-07 |  |  |
| Amplification Peak 58 | 16p13.3 | chr16:2447186-2747857 | 300671 | 8.70E-06 |  |  |
| Amplification Peak 59 | 16p12.1 | chr16:22491335-22602661 | 111326 | 6.55E-05 |  |  |
| Amplification Peak 60 | 16p11.2 | chr16:31939480-33366588 | 1427108 | 6.89E-09 |  |  |
| Amplification Peak 61 | 16q22.2 | chr16:69405876-69755985 | 350109 | 0.00010585 |  |  |
| Amplification Peak 62 | 17p13.1 | chr17:7648062-7782605 | 134543 | 0.0012444 |  |  |
| Amplification Peak 63 | 17p11.2 | chr17:18752334-19091255 | 338921 | 5.99E-07 |  |  |
| Amplification Peak 64 | 17q21.31 | chr17:41655253-41788251 | 132998 | 5.78E-19 |  |  |
| Amplification Peak 65 | 17q21.31 | chr17:41720894-42179459 | 458565 | 4.21E-15 |  |  |
| Amplification Peak 66 | 17q25.3 | chr17:74711608-75436782 | 725174 | 0.00017317 |  |  |
| Amplification Peak 67 | 18q21.1 | chr18:42363427-42469389 | 105962 | 7.26E-06 |  |  |
| Amplification Peak 68 | 19p13.2 | chr19:8842291-8973270 | 130979 | 0.00080061 |  |  |
| Amplification Peak 69 | 19q13.2 | chr19:47380992-47538762 | 157770 | 0.0022224 |  |  |
| Amplification Peak 70 | 20p13 | chr20:1479132-1734626 | 255494 | 0.045938 |  |  |
| Amplification Peak 71 | 20q13.31 | chr20:54999411-55228593 | 229182 | 0.0010722 |  |  |
| Amplification Peak 72 | 21q22.3 | chr21:42481951-42721913 | 239962 | 0.00020602 |  |  |
| Amplification Peak 73 | 22q11.21 | chr22:18883524-19143810 | 260286 | 3.03E-06 |  |  |
| Amplification Peak 74 | 22q11.22 | chr22:21342728-21820910 | 478182 | 0.0012444 |  |  |
| Amplification Peak 75 | Xp22.33 | chrX:3727161-3928515 | 201354 | 4.21E-15 |  |  |
| Amplification Peak 76 | Xp21.1 | chrX:36644121-36721083 | 76962 | 1.95E-12 |  |  |
| Amplification Peak 77 | Xp11.23 | chrX:48429964-48479883 | 49919 | 6.94E-15 |  |  |
| Amplification Peak 78 | Xp11.22 | chrX:52520031-52580680 | 60649 | 5.29E-11 |  |  |
| Amplification Peak 79 | Xq26.3 | chrX:134667634-134826586 | 158952 | 5.28E-13 |  |  |
| Amplification Peak 80 | Xq28 | chrX:151741367-151956295 | 214928 | 4.12E-07 |  |  |
| Amplification Peak 81 | Xq28 | chrX:153012612-153202329 | 189717 | 5.97E-14 |  |  |
| Deletion Peak 1 | 1p36.33 | chr1:1-1018316 | 1018315 | 8.39E-07 |  |  |
| Deletion Peak 2 | 1p36.22 | chr1:9887627-10386762 | 499135 | 0.0014663 |  |  |
| Deletion Peak 3 | 1p36.11 | chr1:25405392-25772739 | 367347 | 0.00012151 |  |  |
| Deletion Peak 4 | 1p12-q21.1 | chr1:120407163-143108109 | 22700946 | 5.26E-06 |  |  |
| Deletion Peak 5 | 1q21.1 | chr1:145938613-147852780 | 1914167 | 2.04E-09 |  |  |
| Deletion Peak 6 | 1q22 | chr1:153508933-154023792 | 514859 | 0.00010938 | *DAP3* |  |
| Deletion Peak 7 | 2p11.2 | chr2:86919832-87842215 | 922383 | 5.27E-05 |  |  |
| Deletion Peak 8 | 2p11.2 | chr2:88244643-95006650 | 6762007 | 7.27E-05 |  |  |
| Deletion Peak 9 | 2q33.1-q33.2 | chr2:202821896-203814028 | 992132 | 0.00098778 |  |  |
| Deletion Peak 10 | 3p26.2 -p26.1 | chr3:4852759-6912690 | 2059931 | 0.018903 |  |  |
| Deletion Peak 11 | 3q22.3 | chr3:139641590-140169936 | 528346 | 0.00022181 |  |  |
| Deletion Peak 12 | 3q26.32 | chr3:176961046-179762212 | 2801166 | 6.06E-05 | *TBL1XR1* |  |
| Deletion Peak 13 | 4p14 | chr4:39290042-39876982 | 586940 | 0.0008178 |  |  |
| Deletion Peak 14 | 4q13.3 | chr4:71719391-72311863 | 592472 | 0.0088126 |  |  |
| Deletion Peak 15 | 4q21.3 -q22.1 | chr4:87997832-88305966 | 308134 | 0.002973 |  |  |
| Deletion Peak 16 | 4q31.22 | chr4:144672120-145788655 | 1116535 | 0.02386 |  | [[10](#_ENREF_10)] |
| Deletion Peak 17 | 5p15.33 | chr5:628466-929826 | 301360 | 0.00034695 |  |  |
| Deletion Peak 18 | 5q13.2 | chr5:69397934-70443511 | 1045577 | 3.87E-16 |  |  |
| Deletion Peak 19 | 5q33.3 | chr5:157214971-158552624 | 1337653 | 7.62E-08 | *EBF1* | [[10-13](#_ENREF_10)] |
| Deletion Peak 20 | 6p25.3 | chr6:1-340019 | 340018 | 4.22E-12 |  |  |
| Deletion Peak 21 | 6p22.1 | chr6:26331758-26481262 | 149504 | 5.00E-07 |  |  |
| Deletion Peak 22 | 6q13 | chr6:73961018-74495457 | 534439 | 0.0019127 |  |  |
| Deletion Peak 23 | 7p22.3 | chr7:695016-1462916 | 767900 | 2.21E-07 |  | [[14](#_ENREF_14)] |
| Deletion Peak 24 | 7p22.1 | chr7:5784298-6038883 | 254585 | 5.25E-08 |  |  |
| Deletion Peak 25 | 7p14.3 -p14.2 | chr7:34871249-35642862 | 771613 | 0.00060509 |  | [[14](#_ENREF_14)] |
| Deletion Peak 26 | 7p12.2 | chr7:50016384-50491610 | 475226 | 1.46E-10 | *IKZF1* | [[10-13](#_ENREF_10)] |
| Deletion Peak 27 | 7q35 | chr7:142847094-143291041 | 443947 | 1.73E-13 |  |  |
| Deletion Peak 28 | 7q35 | chr7:143561053-143738775 | 177722 | 1.21E-24 |  |  |
| Deletion Peak 29 | 8p23.3 | chr8:1-1709396 | 1709395 | 0.00025779 |  |  |
| Deletion Peak 30 | 8p23.1 | chr8:6864662-8228003 | 1363341 | 1.09E-32 |  |  |
| Deletion Peak 31 | 8q21.13 | chr8:81223792-81718685 | 494893 | 0.00098546 |  |  |
| Deletion Peak 32 | 9p21.3 | chr9:21494039-22007872 | 513833 | 1.34E-67 | *CDKN2A/B, MTAP* | [[7](#_ENREF_7), [10-13](#_ENREF_10), [15](#_ENREF_15)] |
| Deletion Peak 33 | 9p13.3 | chr9:33765293-34335864 | 570571 | 1.71E-07 |  |  |
| Deletion Peak 34 | 9q12 | chr9:43013849-67632305 | 24618456 | 1.50E-12 |  |  |
| Deletion Peak 35 | 9q12-q13 | chr9:47091551-70059769 | 22968218 | 6.14E-09 |  |  |
| Deletion Peak 36 | 9q34.11 | chr9:131550403-132004095 | 453692 | 0.0070206 |  |  |
| Deletion Peak 37 | 10p15.3 | chr10:287840-1100328 | 812488 | 0.027373 |  |  |
| Deletion Peak 38 | 10p11.21 | chr10:35949172-38165551 | 2216379 | 0.019151 |  |  |
| Deletion Peak 39 | 10q11.22 | chr10:46057695-47224204 | 1166509 | 3.20E-10 |  |  |
| Deletion Peak 40 | 10q26.3 | chr10:133958131-134818847 | 860716 | 0.00019181 |  |  |
| Deletion Peak 41 | 11p11.2 | chr11:47396598-47973063 | 576465 | 0.008209 |  |  |
| Deletion Peak 42 | 11q11 | chr11:48352876-54878808 | 6525932 | 0.00079419 |  |  |
| Deletion Peak 43 | 11q23.3 | chr11:116175122-116632445 | 457323 | 0.0020416 |  |  |
| Deletion Peak 44 | 11q23.3 | chr11:117639144-117911050 | 271906 | 0.002822 |  |  |
| Deletion Peak 45 | 12p13.33 | chr12:641695-1550190 | 908495 | 0.00095965 |  |  |
| Deletion Peak 46 | 12p13.2-p13.1 | chr12:11240452-13255118 | 2014666 | 0.00094784 | *ETV6, CDKN1B* | [[10-13](#_ENREF_10)] |
| Deletion Peak 47 | 12q21.33-q22 | chr12:90094910-91349761 | 1254851 | 1.75E-08 | *BTG1* | [[11](#_ENREF_11)] |
| Deletion Peak 48 | 12q24.21-q24.22 | chr12:113589074-115464271 | 1875197 | 0.0014663 |  | [[15](#_ENREF_15)] |
| Deletion Peak 49 | 12q24.33 | chr12:131960559-132349534 | 388975 | 0.00095193 |  | [[15](#_ENREF_15)] |
| Deletion Peak 50 | 13q14.2 | chr13:47707970-48206679 | 498709 | 0.0020416 | *RB1* | [[10-13](#_ENREF_10), [15](#_ENREF_15)] |
| Deletion Peak 51 | 13q14.3 | chr13:51473687-52107660 | 633973 | 0.019957 |  | [[10](#_ENREF_10), [15](#_ENREF_15)] |
| Deletion Peak 52 | 13q34 | chr13:110356845-111785722 | 1428877 | 0.00058651 |  |  |
| Deletion Peak 53 | 14q13.2 | chr14:34143096-34842445 | 699349 | 0.00076289 |  |  |
| Deletion Peak 54 | 14q32.33 | chr14:104960404-106368585 | 1408181 | 1.00E-12 | *MTA1* | [[13](#_ENREF_13), [14](#_ENREF_14)] |
| Deletion Peak 55 | 15q11.2 | chr15:1-19927929 | 19927928 | 1.35E-16 |  |  |
| Deletion Peak 56 | 15q13.2 | chr15:27889901-28759043 | 869142 | 6.71E-10 |  |  |
| Deletion Peak 57 | 16p13.3 | chr16:1-1610547 | 1610546 | 0.0045398 |  |  |
| Deletion Peak 58 | 16p12.1 | chr16:22291241-22738295 | 447054 | 0.0032369 |  |  |
| Deletion Peak 59 | 16p11.2 | chr16:31417111-45186808 | 13769697 | 2.05E-07 |  |  |
| Deletion Peak 60 | 16q22.1 | chr16:67278758-67917463 | 638705 | 3.81E-05 |  | [[15](#_ENREF_15)] |
| Deletion Peak 61 | 16q22.1 | chr16:68338792-68924238 | 585446 | 1.43E-05 |  | [[15](#_ENREF_15)] |
| Deletion Peak 62 | 17p13.3 | chr17:1-371298 | 371297 | 0.00060509 |  |  |
| Deletion Peak 63 | 17p11.2 | chr17:18349051-19091255 | 742204 | 2.50E-06 |  |  |
| Deletion Peak 64 | 17q21.31 | chr17:41604760-41854797 | 250037 | 6.11E-37 |  |  |
| Deletion Peak 65 | 18q11.2 | chr18:14129062-18008789 | 3879727 | 0.0016465 |  |  |
| Deletion Peak 66 | 18q23 | chr18:74857550-75839176 | 981626 | 0.034364 |  |  |
| Deletion Peak 67 | 19p13.3 | chr19:1-1780982 | 1780981 | 0.01277 | *TCF3* |  |
| Deletion Peak 68 | 19q13.12 | chr19:41528137-41851945 | 323808 | 0.019957 |  |  |
| Deletion Peak 69 | 20q13.12 | chr20:44784817-45763547 | 978730 | 0.00060749 |  |  |
| Deletion Peak 70 | 20q13.33 | chr20:61418805-62435964 | 1017159 | 0.0014663 |  |  |
| Deletion Peak 71 | 21q11.2 | chr21:1-14424908 | 14424907 | 0.00069114 |  |  |
| Deletion Peak 72 | 22q11.21 | chr22:18450385-19140235 | 689850 | 0.013399 |  |  |
| Deletion Peak 73 | 22q11.21 | chr22:19660347-20447121 | 786774 | 0.014819 | *MAPK1* |  |
| Deletion Peak 74 | 22q11.22 | chr22:20631876-21738494 | 1106618 | 0.019957 | *VPREB1* |  |
| Deletion Peak 75 | Xp22.33 | chrX:1-2160506 | 2160505 | 0.00076289 | *ASMTL, CRLF2, SLC25A6, IL3RA, CSF2RA, P2RY8* |  |
| Deletion Peak 76 | Xp11.22 | chrX:52322502-52745817 | 423315 | 2.14E-13 |  |  |
| Deletion Peak 77 | Xq24 | chrX:118587105-118923706 | 336601 | 8.16E-05 |  |  |
| Deletion Peak 78 | Xq26.3 | chrX:134537499-134889218 | 351719 | 7.62E-08 |  |  |
| Deletion Peak 79 | Xq28 | chrX:153010365-153194575 | 184210 | 1.40E-17 |  |  |

**Table E. Regions of significant recurrent amplification and deletion in the whole cohort of children with B-ALL (n=115) (q<0.05)**

| **Unique name** | **Cytoband** | **Wide peak limits** | **Peak boundaries (Mb)** | **q values** | **Candidate target gene(s)** | **Alterations referenced in other studies** |
| --- | --- | --- | --- | --- | --- | --- |
| Amplification Peak 1 | 1p36.33 | chr1:1-1069257 | 1069256 | 2.21E-05 |  |  |
| Amplification Peak 2 | 1p12 | chr1:120280136-120586489 | 306353 | 1.96E-05 |  |  |
| Amplification Peak 3 | 1q21.1 | chr1:146238438-147304143 | 1065705 | 3.80E-08 |  | [[4](#_ENREF_4), [6](#_ENREF_6), [16](#_ENREF_16)] |
| Amplification Peak 4 | 1q32.1 | chr1:202728890-203615042 | 886152 | 0.0032099 |  | [[4](#_ENREF_4), [6](#_ENREF_6), [16](#_ENREF_16)] |
| Amplification Peak 5 | 2p11.2 | chr2:87210773-87764945 | 554172 | 0.00020586 |  |  |
| Amplification Peak 6 | 2q11.2 | chr2:95746881-96057830 | 310949 | 0.0021957 |  |  |
| Amplification Peak 7 | 3p25.1 | chr3:13123271-13889579 | 766308 | 0.00095084 |  |  |
| Amplification Peak 8 | 4p16.1 | chr4:9698722-9937752 | 239030 | 0.049599 |  |  |
| Amplification Peak 9 | 5p15.33 | chr5:1-929826 | 929825 | 0.0045908 |  |  |
| Amplification Peak 10 | 5q13.2 | chr5:69471686-70271395 | 799709 | 3.46E-07 |  | [[16](#_ENREF_16)] |
| Amplification Peak 11 | 6p25.3 | chr6:1-288976 | 288975 | 3.47E-05 |  | [[4](#_ENREF_4)] |
| Amplification Peak 12 | 6p21.1 | chr6:40824099-41226769 | 402670 | 0.00048589 |  |  |
| Amplification Peak 13 | 6q27 | chr6:168074431-168353515 | 279084 | 0.022693 |  |  |
| Amplification Peak 14 | 7p22.3 | chr7:1-721398 | 721397 | 0.03922 |  |  |
| Amplification Peak 15 | 7q22.1 | chr7:101852347-102125707 | 273360 | 7.24E-26 |  |  |
| Amplification Peak 16 | 7q35 | chr7:143528315-143708603 | 180288 | 7.76E-20 |  |  |
| Amplification Peak 17 | 8p23.1 | chr8:6770696-7199608 | 428912 | 2.97E-06 |  |  |
| Amplification Peak 18 | 8p23.1 | chr8:7240497-7336245 | 95748 | 1.32E-27 |  |  |
| Amplification Peak 19 | 8q24.3 | chr8:142293632-143259887 | 966255 | 0.0032099 |  |  |
| Amplification Peak 20 | 9p13.1 | chr9:38897716-38987239 | 89523 | 1.12E-16 |  |  |
| Amplification Peak 21 | 9p11.2 | chr9:45182226-46567507 | 1385281 | 3.61E-12 |  |  |
| Amplification Peak 22 | 9q12 | chr9:65495416-65720561 | 225145 | 2.68E-17 |  |  |
| Amplification Peak 23 | 10q11.22 | chr10:46090376-46678317 | 587941 | 2.51E-05 |  |  |
| Amplification Peak 24 | 10q22.1 | chr10:72326342-72662418 | 336076 | 0.0019024 |  |  |
| Amplification Peak 25 | 11q25 | chr11:133846180-134265155 | 418975 | 0.0032099 |  |  |
| Amplification Peak 26 | 12q13.13 | chr12:50712382-51500995 | 788613 | 0.0041162 |  |  |
| Amplification Peak 27 | 14q11.2 | chr14:21878477-21941919 | 63442 | 3.57E-38 |  |  |
| Amplification Peak 28 | 15q11.2 | chr15:1-19927929 | 19927928 | 1.20E-15 |  |  |
| Amplification Peak 29 | 16p12.1 | chr16:22355503-22628366 | 272863 | 0.0091507 |  |  |
| Amplification Peak 30 | 16p11.2 | chr16:31939480-33366588 | 1427108 | 1.94E-12 |  |  |
| Amplification Peak 31 | 16q22.2 | chr16:69384372-69779277 | 394905 | 0.0051411 |  |  |
| Amplification Peak 32 | 17p11.2 | chr17:18881246-19075657 | 194411 | 0.012965 |  |  |
| Amplification Peak 33 | 17q21.31 | chr17:41596803-41755951 | 159148 | 1.91E-06 |  | [[6](#_ENREF_6)] |
| Amplification Peak 34 | 17q21.31 | chr17:41720894-41854797 | 133903 | 1.25E-11 |  | [[6](#_ENREF_6)] |
| Amplification Peak 35 | 22q11.23 | chr22:23926603-24259811 | 333208 | 0.0058214 |  | [[4](#_ENREF_4)] |
| Amplification Peak 36 | Xp22.33 | chrX:3727161-3928515 | 201354 | 1.54E-26 |  |  |
| Amplification Peak 37 | Xp21.1 | chrX:36588830-36721083 | 132253 | 0.00027854 |  |  |
| Amplification Peak 38 | Xp11.23 | chrX:48426949-48479883 | 52934 | 1.35E-05 |  |  |
| Amplification Peak 39 | Xp11.22 | chrX:52520031-52580680 | 60649 | 2.88E-09 |  |  |
| Amplification Peak 40 | Xq26.3 | chrX:134667634-134832139 | 164505 | 5.68E-14 |  |  |
| Amplification Peak 41 | Xq28 | chrX:153012612-153202329 | 189717 | 1.80E-18 |  |  |
| Deletion Peak 1 | 1p36.11 | chr1:25161288-25772739 | 611451 | 5.21E-05 |  |  |
| Deletion Peak 2 | 1p12 | chr1:120220612-144126558 | 23905946 | 0.00070562 |  |  |
| Deletion Peak 3 | 1q21.1 | chr1:145938613-147852780 | 1914167 | 7.18E-08 |  |  |
| Deletion Peak 4 | 1q21.2 | chr1:148063116-148145186 | 82070 | 3.32E-07 |  |  |
| Deletion Peak 5 | 2p11.2 | chr2:87002376-88108759 | 1106383 | 0.00052436 |  | [[16](#_ENREF_16)] |
| Deletion Peak 6 | 2p11.2 | chr2:88677247-95006650 | 6329403 | 0.00025941 |  | [[16](#_ENREF_16)] |
| Deletion Peak 7 | 2q11.1 | chr2:88819812-95076663 | 6256851 | 2.33E-06 |  |  |
| Deletion Peak 8 | 2q13 | chr2:111639109-112392452 | 753343 | 0.0203 |  |  |
| Deletion Peak 9 | 3p21.31 | chr3:46988351-50582891 | 3594540 | 0.031041 | *SETD2* | [[6](#_ENREF_6)] |
| Deletion Peak 10 | 3q26.32 | chr3:176961046-179762212 | 2801166 | 0.00012903 | *TBL1XR1* | [[4](#_ENREF_4), [17](#_ENREF_17)] |
| Deletion Peak 11 | 5p15.33 | chr5:1-2809848 | 2809847 | 0.031041 |  |  |
| Deletion Peak 12 | 5q13.2 | chr5:68897383-70841211 | 1943828 | 4.64E-16 |  | [[16](#_ENREF_16)] |
| Deletion Peak 13 | 5q23.2 | chr5:122972636-125793304 | 2820668 | 0.02638 |  |  |
| Deletion Peak 14 | 6p25.3 | chr6:1-340019 | 340018 | 0.00056364 |  |  |
| Deletion Peak 15 | 6q16.3 | chr6:84196466-116374594 | 32178128 | 0.0023118 | *FYN, FOXO3a, GRIK2, EPHA7, BLIMP1* | [[4](#_ENREF_4), [6](#_ENREF_6)] |
| Deletion Peak 16 | 7p22.3 | chr7:1-2692735 | 2692734 | 0.013712 |  |  |
| Deletion Peak 17 | 7p12.2 | chr7:49915313-51062174 | 1146861 | 0.0050684 | *IKZF1* | [[4](#_ENREF_4), [6](#_ENREF_6), [16](#_ENREF_16), [18](#_ENREF_18), [19](#_ENREF_19)] |
| Deletion Peak 18 | 7p11.2 | chr7:55599730-64109382 | 8509652 | 0.0015948 |  |  |
| Deletion Peak 19 | 7q22.1 | chr7:101816673-102245823 | 429150 | 1.41E-07 |  |  |
| Deletion Peak 20 | 7q34 | chr7:141583326-142286761 | 703435 | 1.82E-10 |  | [[16](#_ENREF_16)] |
| Deletion Peak 21 | 7q35 | chr7:142847094-143291041 | 443947 | 1.18E-06 |  |  |
| Deletion Peak 22 | 7q35 | chr7:143454900-143738775 | 283875 | 3.92E-22 |  |  |
| Deletion Peak 23 | 8p23.1 | chr8:6864662-8228003 | 1363341 | 1.48E-21 |  |  |
| Deletion Peak 24 | 9p21.3 | chr9:21283102-22007872 | 724770 | 1.15E-31 | *CDKN2A, CDKN2B, IFN, MTAP* | [[4](#_ENREF_4), [6](#_ENREF_6), [8](#_ENREF_8), [9](#_ENREF_9), [16-19](#_ENREF_16)] |
| Deletion Peak 25 | 9q12 | chr9:43013849-70059769 | 27045920 | 9.49E-09 |  |  |
| Deletion Peak 26 | 9q34.11 | chr9:129143576-132004095 | 2860519 | 0.04551 |  |  |
| Deletion Peak 27 | 10p11.21 | chr10:35949172-42904196 | 6955024 | 0.026213 |  |  |
| Deletion Peak 28 | 10q11.22 | chr10:46093738-47224204 | 1130466 | 4.82E-10 |  |  |
| Deletion Peak 29 | 11p11.12 | chr11:48352876-54957910 | 6605034 | 0.00087044 |  |  |
| Deletion Peak 30 | 11q14.3 | chr11:87678990-89826181 | 2147191 | 0.047413 |  | [[6](#_ENREF_6)] |
| Deletion Peak 31 | 11q23.3 | chr11:117849873-118493305 | 643432 | 0.02638 | *MLL* | [[4](#_ENREF_4), [6](#_ENREF_6)] |
| Deletion Peak 32 | 12p13.2 | chr12:11240452-12374611 | 1134159 | 3.78E-12 | *ETV6* | [[4](#_ENREF_4), [6](#_ENREF_6), [16](#_ENREF_16), [18](#_ENREF_18), [19](#_ENREF_19)] |
| Deletion Peak 33 | 12q21.33 | chr12:90094910-91349761 | 1254851 | 0.00014225 | *BTG1* | [[4](#_ENREF_4), [6](#_ENREF_6)] |
| Deletion Peak 34 | 12q24.33 | chr12:130166382-132150169 | 1983787 | 0.028262 |  |  |
| Deletion Peak 35 | 14q32.33 | chr14:104960404-106368585 | 1408181 | 0.0026421 | *MTA1* | [[16](#_ENREF_16), [18](#_ENREF_18)] |
| Deletion Peak 36 | 15q11.2 | chr15:1-20311079 | 20311078 | 4.64E-16 |  |  |
| Deletion Peak 37 | 15q13.3 | chr15:29734139-30704995 | 970856 | 0.011399 |  |  |
| Deletion Peak 38 | 16p12.1 | chr16:22291241-22738295 | 447054 | 0.004682 |  |  |
| Deletion Peak 39 | 16p11.2 | chr16:31417111-33114651 | 1697540 | 0.00018314 |  |  |
| Deletion Peak 40 | 16q22.1 | chr16:67025632-68924238 | 1898606 | 0.011473 |  | [[4](#_ENREF_4)] |
| Deletion Peak 41 | 17p11.2 | chr17:18623862-19091255 | 467393 | 0.0023118 |  | [[16](#_ENREF_16)] |
| Deletion Peak 42 | 17q21.32 | chr17:41788253-42230265 | 442012 | 6.24E-41 |  |  |
| Deletion Peak 43 | 19p13.3 | chr19:1009412-1780982 | 771570 | 3.32E-07 | *E2A (TCF3)* | [[4](#_ENREF_4)] |
| Deletion Peak 44 | 20q13.33 | chr20:60048310-62435964 | 2387654 | 0.025486 |  | [[16](#_ENREF_16), [19](#_ENREF_19)] |
| Deletion Peak 45 | 22q11.22 | chr22:20926300-21338812 | 412512 | 2.92E-08 | *VPREB1* |  |
| Deletion Peak 46 | Xp22.33 | chrX:1287039-2160506 | 873467 | 0.00014225 | *IL3RA, CRLF2, CSF2RA, CSF2RA, P2RY8, ASMTL, SLC25A6* | [[17](#_ENREF_17)] |
| Deletion Peak 47 | Xp11.22 | chrX:52322502-52745817 | 423315 | 2.15E-06 |  |  |
| Deletion Peak 48 | Xq26.3 | chrX:134537499-134896772 | 359273 | 7.67E-07 |  |  |
| Deletion Peak 49 | Xq28 | chrX:153010365-153299880 | 289515 | 1.30E-18 | *RPL10* |  |

**Table F. Regions of significant recurrent amplification and deletion in the whole cohort of adults with B-ALL (n=100) (q<0.05)**

| **Unique name** | **Cytoband** | **Wide peak limits** | **Peak boundaries (Mb)** | **q values** | **Candidate target gene(s)** | **Alterations referenced in other studies** |
| --- | --- | --- | --- | --- | --- | --- |
| Amplification Peak 1 | 1p36.13 | chr1:17747107-18782258 | 1035151 | 0.008641 |  |  |
| Amplification Peak 2 | 1p36.11 | chr1:25405392-25640963 | 235571 | 0.0072211 |  |  |
| Amplification Peak 3 | 1p12 | chr1:120280136-143614919 | 23334783 | 0.0072211 |  |  |
| Amplification Peak 4 | 1q21.2 | chr1:146145352-148097698 | 1952346 | 0.0076056 |  |  |
| Amplification Peak 5 | 1q32.1 | chr1:202797673-203615042 | 817369 | 0.001434 |  |  |
| Amplification Peak 6 | 2p11.2 | chr2:85751968-85959400 | 207432 | 0.00013941 |  |  |
| Amplification Peak 7 | 2p11.2 | chr2:87210773-87515141 | 304368 | 0.019537 |  |  |
| Amplification Peak 8 | 2p11.2 | chr2:88819812-89145543 | 325731 | 0.012872 |  |  |
| Amplification Peak 9 | 3p25.3 | chr3:10294940-11096335 | 801395 | 0.0048729 |  |  |
| Amplification Peak 10 | 4p16.1 | chr4:9737554-9937752 | 200198 | 0.0048729 |  |  |
| Amplification Peak 11 | 5p15.33 | chr5:1-929826 | 929825 | 0.0039204 |  |  |
| Amplification Peak 12 | 5q13.2 | chr5:68879577-70732403 | 1852826 | 5.20E-06 |  |  |
| Amplification Peak 13 | 5q31.3 | chr5:138834932-139347376 | 512444 | 0.014235 |  |  |
| Amplification Peak 14 | 6p25.3 | chr6:1-340019 | 340018 | 0.0016567 |  |  |
| Amplification Peak 15 | 6p21.1 | chr6:40731192-41122077 | 390885 | 3.18E-05 |  |  |
| Amplification Peak 16 | 6q27 | chr6:168075913-168341186 | 265273 | 0.00044512 |  |  |
| Amplification Peak 17 | 7p22.3 | chr7:1-683456 | 683455 | 0.010635 |  |  |
| Amplification Peak 18 | 7q22.1 | chr7:101857302-102096490 | 239188 | 1.73E-14 |  |  |
| Amplification Peak 19 | 7q35 | chr7:142942379-143234018 | 291639 | 0.00012432 |  |  |
| Amplification Peak 20 | 7q35 | chr7:143529099-143705112 | 176013 | 6.80E-18 |  |  |
| Amplification Peak 21 | 8p23.1 | chr8:6773736-7199608 | 425872 | 6.35E-06 |  |  |
| Amplification Peak 22 | 8p23.1 | chr8:7217189-7780825 | 563636 | 2.05E-13 |  |  |
| Amplification Peak 23 | 8q24.3 | chr8:142293632-143211592 | 917960 | 0.0025914 |  | [[10](#_ENREF_10)] |
| Amplification Peak 24 | 9p13.1 | chr9:38710557-38987239 | 276682 | 0.00010718 |  |  |
| Amplification Peak 25 | 9p11.2 | chr9:43533913-43817801 | 283888 | 1.44E-06 |  |  |
| Amplification Peak 26 | 9q12 | chr9:65495416-65720561 | 225145 | 2.16E-06 |  |  |
| Amplification Peak 27 | 9q34.2 | chr9:132680717-140273252 | 7592535 | 0.0076056 | *ABL1, NOTCH1* | [[10](#_ENREF_10)] |
| Amplification Peak 28 | 10q11.22 | chr10:46093738-47055209 | 961471 | 0.0016567 |  |  |
| Amplification Peak 29 | 10q22.3 | chr10:79717933-80939068 | 1221135 | 7.29E-05 |  |  |
| Amplification Peak 30 | 11p11.2 | chr11:44291778-45705036 | 1413258 | 0.0097563 |  |  |
| Amplification Peak 31 | 11q13.1 | chr11:64110077-64470933 | 360856 | 0.0097563 |  |  |
| Amplification Peak 32 | 11q25 | chr11:133846180-134265155 | 418975 | 0.00091592 |  |  |
| Amplification Peak 33 | 12p13.31 | chr12:5960626-7195121 | 1234495 | 0.02829 |  |  |
| Amplification Peak 34 | 12q13.13 | chr12:50675756-51596331 | 920575 | 0.0012494 |  |  |
| Amplification Peak 35 | 14q11.2 | chr14:21766590-21941919 | 175329 | 6.26E-42 |  |  |
| Amplification Peak 36 | 15q11.2 | chr15:1-19501824 | 19501823 | 2.72E-12 |  |  |
| Amplification Peak 37 | 15q23 | chr15:67611385-68211561 | 600176 | 0.0061023 |  |  |
| Amplification Peak 38 | 16p13.3 | chr16:1-1684220 | 1684219 | 0.006468 |  |  |
| Amplification Peak 39 | 16p11.2 | chr16:31950858-34141409 | 2190551 | 4.94E-05 |  |  |
| Amplification Peak 40 | 16q22.2 | chr16:69385462-69779277 | 393815 | 0.022071 |  |  |
| Amplification Peak 41 | 17p11.2 | chr17:18881246-19107433 | 226187 | 0.00032508 |  |  |
| Amplification Peak 42 | 17q21.31 | chr17:41618430-41755951 | 137521 | 9.73E-12 |  |  |
| Amplification Peak 43 | 17q21.32 | chr17:41755953-42230265 | 474312 | 9.15E-10 |  |  |
| Amplification Peak 44 | 18q21.1 | chr18:44439405-44648785 | 209380 | 0.014235 |  |  |
| Amplification Peak 45 | 19p13.2 | chr19:8842291-8977814 | 135523 | 0.049213 |  |  |
| Amplification Peak 46 | 20p13 | chr20:1479132-1817220 | 338088 | 0.049213 |  |  |
| Amplification Peak 47 | 21q22.3 | chr21:44691770-45864829 | 1173059 | 0.035193 |  |  |
| Amplification Peak 48 | 22q11.21 | chr22:18752219-19143810 | 391591 | 0.00044512 |  |  |
| Amplification Peak 49 | Xp22.33 | chrX:3727161-3928515 | 201354 | 1.07E-09 |  |  |
| Amplification Peak 50 | Xp11.4 | chrX:38965925-39412808 | 446883 | 0.00015778 |  |  |
| Amplification Peak 51 | Xp11.23 | chrX:47734204-47887269 | 153065 | 6.40E-07 |  |  |
| Amplification Peak 52 | Xp11.22 | chrX:52520031-52667743 | 147712 | 0.00091592 |  |  |
| Amplification Peak 53 | Xq26.3 | chrX:134667634-134826586 | 158952 | 7.86E-12 |  |  |
| Amplification Peak 54 | Xq28 | chrX:151741367-151984941 | 243574 | 0.002204 |  |  |
| Amplification Peak 55 | Xq28 | chrX:153012612-153202329 | 189717 | 5.30E-18 |  |  |
| Deletion Peak 1 | 1p36.33 | chr1:1-1018316 | 1018315 | 2.34E-05 |  |  |
| Deletion Peak 2 | 1p36.11 | chr1:25405392-25772739 | 367347 | 0.00028006 |  |  |
| Deletion Peak 3 | 1p12 | chr1:120407163-143582445 | 23175282 | 5.76E-05 |  |  |
| Deletion Peak 4 | 1q21.1 | chr1:145938613-147852780 | 1914167 | 5.21E-07 |  |  |
| Deletion Peak 5 | 2p11.2 | chr2:86919832-87842215 | 922383 | 0.002666 |  |  |
| Deletion Peak 6 | 2p11.2 | chr2:88677247-95006650 | 6329403 | 5.26E-06 |  |  |
| Deletion Peak 7 | 2q11.1 | chr2:88819812-95076663 | 6256851 | 0.0015431 |  |  |
| Deletion Peak 8 | 2q33.1 | chr2:202610740-203814028 | 1203288 | 0.049022 |  |  |
| Deletion Peak 9 | 3q29 | chr3:196604633-199501827 | 2897194 | 0.030599 | *MUC4* |  |
| Deletion Peak 10 | 4p14 | chr4:38947349-39876982 | 929633 | 0.039158 |  |  |
| Deletion Peak 11 | 5q13.2 | chr5:68897383-70367244 | 1469861 | 1.07E-11 |  |  |
| Deletion Peak 12 | 5q33.3 | chr5:157214971-158552624 | 1337653 | 4.02E-05 | *EBF1* | [[10](#_ENREF_10)] |
| Deletion Peak 13 | 6p25.3 | chr6:1-340019 | 340018 | 7.79E-08 |  |  |
| Deletion Peak 14 | 6p22.1 | chr6:26331758-26481262 | 149504 | 8.99E-05 |  |  |
| Deletion Peak 15 | 7p22.1 | chr7:5881089-6185296 | 304207 | 0.00046359 |  |  |
| Deletion Peak 16 | 7p12.2 | chr7:50016384-50491610 | 475226 | 1.02E-05 | *IKZF1* | [[10](#_ENREF_10)] |
| Deletion Peak 17 | 7q11.21 | chr7:63781191-64990938 | 1209747 | 0.0080183 |  |  |
| Deletion Peak 18 | 7q35 | chr7:142847094-143291041 | 443947 | 2.40E-10 |  |  |
| Deletion Peak 19 | 7q35 | chr7:143561053-143738775 | 177722 | 1.97E-20 |  |  |
| Deletion Peak 20 | 8p23.1 | chr8:6864662-7293525 | 428863 | 2.00E-20 |  |  |
| Deletion Peak 21 | 8q21.13 | chr8:81223792-81646456 | 422664 | 0.040171 |  |  |
| Deletion Peak 22 | 9p21.3 | chr9:21494039-22007872 | 513833 | 1.55E-52 | *CDKN2B, CDKN2A, MTAP* | [[10](#_ENREF_10), [15](#_ENREF_15)] |
| Deletion Peak 23 | 9q12 | chr9:43013849-70059769 | 27045920 | 3.89E-09 |  |  |
| Deletion Peak 24 | 10q11.22 | chr10:46057695-47224204 | 1166509 | 5.83E-06 |  |  |
| Deletion Peak 25 | 11p11.12 | chr11:48352876-54957910 | 6605034 | 0.0025457 |  |  |
| Deletion Peak 26 | 12q21.33 | chr12:90094910-91349761 | 1254851 | 1.05E-07 | *BTG1* |  |
| Deletion Peak 27 | 13q14.2 | chr13:47707970-48206679 | 498709 | 0.037492 | *RB1* | [[10](#_ENREF_10)] |
| Deletion Peak 28 | 14q32.33 | chr14:104960404-106368585 | 1408181 | 0.00023952 | *MTA1* |  |
| Deletion Peak 29 | 15q11.2 | chr15:1-19358754 | 19358753 | 8.26E-12 |  |  |
| Deletion Peak 30 | 15q13.2 | chr15:27889901-28759043 | 869142 | 0.00014721 |  |  |
| Deletion Peak 31 | 16p13.3 | chr16:1-4181019 | 4181018 | 0.034821 | *CREBBP* |  |
| Deletion Peak 32 | 16p11.2 | chr16:31738698-45186808 | 13448110 | 8.62E-07 |  |  |
| Deletion Peak 33 | 16q22.1 | chr16:68338792-68995478 | 656686 | 0.010421 |  |  |
| Deletion Peak 34 | 17p11.2 | chr17:18623862-18899702 | 275840 | 0.00058201 |  |  |
| Deletion Peak 35 | 17q21.31 | chr17:41604760-41854797 | 250037 | 4.44E-36 |  |  |
| Deletion Peak 36 | 17q21.32 | chr17:41788253-42230265 | 442012 | 1.06E-35 |  |  |
| Deletion Peak 37 | 19p13.3 | chr19:1-1619519 | 1619518 | 0.026982 | *E2A, TCF3* |  |
| Deletion Peak 38 | 20q13.33 | chr20:61104974-62435964 | 1330990 | 0.037492 |  |  |
| Deletion Peak 39 | 21q11.2 | chr21:1-14424908 | 14424907 | 0.022464 |  |  |
| Deletion Peak 40 | 22q11.22 | chr22:20631876-21738494 | 1106618 | 0.041907 | *VPREB1* |  |
| Deletion Peak 41 | Xp22.33 | chrX:1-2625810 | 2625809 | 0.019432 | *SHOX, IL3RA, CRLF2, CSF2RA, P2RY8, ASMTL, SLC25A6* |  |
| Deletion Peak 42 | Xp11.22 | chrX:52322502-52745817 | 423315 | 1.32E-10 |  |  |
| Deletion Peak 43 | Xq26.3 | chrX:134537499-134889218 | 351719 | 1.76E-06 |  |  |
| Deletion Peak 44 | Xq28 | chrX:153010365-153194575 | 184210 | 5.63E-15 |  |  |

**Table G. Regions of significant recurrent amplification and deletion in the whole cohort of children with T-ALL (n=27) (q<0.05)**

| **Unique name** | **Cytoband** | **Wide peak limits** | **Peak boundaries (Mb)** | **q values** | **Candidate target gene(s)** | **Alterations referenced in other studies** |
| --- | --- | --- | --- | --- | --- | --- |
| Amplification Peak 1 | 1q21.1 | chr1:146192984-147304143 | 1111159 | 0.011918 |  |  |
| Amplification Peak 2 | 7q35 | chr7:143528315-143708603 | 180288 | 5.06E-07 |  |  |
| Amplification Peak 3 | 8p23.1 | chr8:7217189-7880532 | 663343 | 8.71E-05 |  |  |
| Amplification Peak 4 | 9p13.1 | chr9:38880046-38987239 | 107193 | 1.36E-05 |  |  |
| Amplification Peak 5 | 9p11.2 | chr9:43533913-43817801 | 283888 | 0.0016344 |  |  |
| Amplification Peak 6 | 14q32.33 | chr14:103842701-105053955 | 1211254 | 0.00062998 | *MTA1* |  |
| Amplification Peak 7 | 15q11.2 | chr15:1-19993265 | 19993264 | 4.67E-06 |  |  |
| Amplification Peak 8 | 16p11.2 | chr16:31738698-34139878 | 2401180 | 0.00062998 |  |  |
| Amplification Peak 9 | 17q21.31 | chr17:41709706-41819693 | 109987 | 1.04E-09 |  |  |
| Amplification Peak 10 | Xq26.3 | chrX:134665842-134826586 | 160744 | 0.00055039 |  |  |
| Amplification Peak 11 | Xq28 | chrX:153012612-153202329 | 189717 | 9.63E-05 |  |  |
| Deletion Peak 1 | 1p36.11 | chr1:25161288-25772739 | 611451 | 0.011223 |  |  |
| Deletion Peak 2 | 5q13.2 | chr5:68691577-70841211 | 2149634 | 0.014646 |  |  |
| Deletion Peak 3 | 7q34 | chr7:141583326-142286761 | 703435 | 4.38E-11 |  |  |
| Deletion Peak 4 | 8p23.1 | chr8:6864662-8228003 | 1363341 | 2.60E-10 |  |  |
| Deletion Peak 5 | 9p21.3 | chr9:21494039-22441867 | 947828 | 6.37E-26 | *CDKN2B, CDKN2A, MTAP, DMRTA1* | [[4](#_ENREF_4), [6](#_ENREF_6)] |
| Deletion Peak 6 | 10q11.21 | chr10:45613179-47224204 | 1611025 | 0.0033731 |  |  |
| Deletion Peak 7 | 11p11.2 | chr11:47396598-47634695 | 238097 | 0.046142 |  |  |
| Deletion Peak 8 | 16q22.1 | chr16:66124962-67917463 | 1792501 | 0.0012011 | *CTCF* | [[4](#_ENREF_4)] |
| Deletion Peak 9 | 17q21.31 | chr17:41604760-42230265 | 625505 | 0.000118 |  |  |
| Deletion Peak 10 | 19p13.2 | chr19:10669513-11737858 | 1068345 | 0.042892 | *DNM2, EPOR* |  |
| Deletion Peak 11 | Xq28 | chrX:153010365-153299880 | 289515 | 1.72E-16 | *RPL10* |  |

**Table H. Regions of significant recurrent amplification and deletion in the whole cohort of adult with T-ALL (n=23) (q<0.05)**

| **Unique name** | **Cytoband** | **Wide peak limits** | **Peak boundaries (Mb)** | **q values** | **Candidate target gene(s)** | **Alterations referenced in other studies** |
| --- | --- | --- | --- | --- | --- | --- |
| Amplification Peak 1 | 1p32.3 | chr1:54552841-55184960 | 632119 | 0.014219 |  |  |
| Amplification Peak 2 | 6q27 | chr6:168074431-168341186 | 266755 | 0.045833 |  |  |
| Amplification Peak 3 | 7q35 | chr7:143528315-143712419 | 184104 | 0.00010825 |  |  |
| Amplification Peak 4 | 8q24.3 | chr8:139653372-141065903 | 1412531 | 0.0099799 |  | [[10](#_ENREF_10)] |
| Amplification Peak 5 | 9p13.1 | chr9:40063942-40327985 | 264043 | 1.65E-05 |  |  |
| Amplification Peak 6 | 9q34.13 | chr9:132564529-133101148 | 536619 | 0.01154 | *ABL1* | [[10](#_ENREF_10)] |
| Amplification Peak 7 | 14q11.2 | chr14:21444378-21987397 | 543019 | 0.021566 |  |  |
| Amplification Peak 8 | 15q11.2 | chr15:1-19929017 | 19929016 | 6.27E-08 |  |  |
| Amplification Peak 9 | 17q21.31 | chr17:41566477-41819693 | 253216 | 0.00017523 |  |  |
| Amplification Peak 10 | Xp11.22 | chrX:52520031-52580680 | 60649 | 0.00010825 |  |  |
| Amplification Peak 11 | Xq28 | chrX:153012612-153202329 | 189717 | 0.013274 |  |  |
| Deletion Peak 1 | 7q34 | chr7:141583326-142286761 | 703435 | 0.0080501 |  |  |
| Deletion Peak 2 | 8p23.1 | chr8:6864662-8228003 | 1363341 | 3.61E-06 |  |  |
| Deletion Peak 3 | 9p21.3 | chr9:21820890-22441867 | 620977 | 3.03E-20 | *CDKN2B, CDKN2A, MTAP, DMRTA1* | [[10](#_ENREF_10), [15](#_ENREF_15)] |
| Deletion Peak 4 | 17q21.31 | chr17:41604760-42230265 | 625505 | 9.96E-06 |  |  |
| Deletion Peak 5 | Xq28 | chrX:153010365-153299880 | 289515 | 0.00010052 | *RPL10* |  |

**Table I. CNAs associated with shorter OS in the groups of childhood patients with ALL**

| **Whole cohort of children with ALL (n=142)** | | | | | | |
| --- | --- | --- | --- | --- | --- | --- |
| **Unique name** | **Cytoband** | **Patients (n)** | **5-year death (n)** | **Median** | **5-year % OS** | **p**  **(Log-rank)** |
| **Deletion Peak 51** | 14q32.33 |  |  |  |  | 0.019 |
|  | No | 96 | 11 | NR | 88.5 |  |
|  | Yes | 30 | 7 | NR | 76.7 |  |
| **Deletion Peak 53** | 15q13.2 |  |  |  |  | 0.04 |
|  | No | 101 | 12 | NR | 88.1 |  |
|  | Yes | 25 | 6 | 96 | 76.0 |  |
| **Child group without good- or poor-risk cytogenetic^1^ (n=82)** | | | | | | |
| **Unique name** | **Cytoband** | **Patients (n)** | **5-year death (n)** | **Median** | **5-year % OS** | **p**  **(Log-rank)** |
| **Amplification Peak 2** | 1p36.11 |  |  |  |  | 0.036 |
|  | No | 55 | 5 | NR | 90.9 |  |
|  | Yes | 17 | 5 | NR | 70.6 |  |
| **Deletion Peak 19** | 6p25.3 |  |  |  |  | 0.032 |
|  | No | 62 | 7 | NR | 88.7 |  |
|  | Yes | 10 | 3 | 96 | 70.0 |  |
| **Deletion Peak 53** | 15q13.2 |  |  |  |  | 0.008 |
|  | No | 60 | 6 | NR | 90.0 |  |
|  | Yes | 12 | 4 | 96 | 66.7 |  |
| **Deletion Peak 55** | 16p13.11 |  |  |  |  | 0.021 |
|  | No | 55 | 5 | NR | 90.9 |  |
|  | Yes | 17 | 5 | NR | 70.6 |  |
| **Deletion Peak 58** | 17p13.1 |  |  |  |  | 0.027 |
|  | No | 63 | 8 | NR | 87.3 |  |
|  | Yes | 9 | 2 | 96 | 77.8 |  |
| ^1^ Includes 82 children with normal cytogenetics (n=67) and other abnormalities (n=15). This group excludes children with good-risk cytogenetics: hyperdiploidy and t(12;21) and poor-risk cytogenetics: t(9;22), t(v;11q23) and hypodiploidy. **Abbreviation**: NR, not reached. | | | | | | |

**Table J. CNAs associated with shorter OS in the groups of adult patients with ALL**

| **Whole cohort of adults with ALL (n=123)** | | | | | | |
| --- | --- | --- | --- | --- | --- | --- |
| **Unique name** | **Cytoband** | **Patients (n)** | **5-year death (n)** | **Median** | **5-year % OS** | **p**  **(Log-rank)** |
| **Amplification Peak 7** | 2p13.3 |  |  |  |  | 0.033 |
|  | No | 80 | 44 | 24 | 45.0 |  |
|  | Yes | 25 | 17 | 10 | 32.0 |  |
| **Amplification Peak 24** | 6p21.1 |  |  |  |  | 0.013 |
|  | No | 52 | 24 | 46 | 53.8 |  |
|  | Yes | 53 | 37 | 14 | 30.2 |  |
| **Amplification Peak 45** | 11p15.1 |  |  |  |  | 0.035 |
|  | No | 76 | 41 | 24 | 46.1 |  |
|  | Yes | 29 | 20 | 14 | 31.0 |  |
| **Amplification Peak 69** | 19q13.2 |  |  |  |  | 0.011 |
|  | No | 64 | 31 | 29 | 51.6 |  |
|  | Yes | 41 | 30 | 13 | 26.8 |  |
| **Amplification Peak 76** | Xp21.1 |  |  |  |  | 0.002 |
|  | No | 52 | 23 | 60 | 55.8 |  |
|  | Yes | 53 | 38 | 13 | 28.3 |  |
| **Deletion Peak 11** | 3q22.3 |  |  |  |  | 0.002 |
|  | No | 74 | 36 | 29 | 51.4 |  |
|  | Yes | 31 | 25 | 11 | 19.4 |  |
| **Deletion Peak 12** | 3q26.32 |  |  |  |  | 0.013 |
|  | No | 73 | 37 | 29 | 49.3 |  |
|  | Yes | 32 | 24 | 13 | 25.0 |  |
| **Deletion Peak 31** | 8q21.13 |  |  |  |  | 0.007 |
|  | No | 81 | 42 | 27 | 48.1 |  |
|  | Yes | 24 | 19 | 6 | 20.8 |  |
| **Deletion Peak 50** | 13q14.2 |  |  |  |  | 0.002 |
|  | No | 84 | 44 | 24 | 47.6 |  |
|  | Yes | 21 | 17 | 12 | 19.0 |  |
| **Broad Deletion** | 17p |  |  |  |  | 0.017 |
|  | No | 97 | 54 | 20 | 44.3 |  |
|  | Yes | 8 | 7 | 1 | 12.5 |  |
| **Adults without poor-risk cytogenetic ^1^ (n=67)** | | | | | | |
| **Unique name** | **Cytoband** | **Patients (n)** | **5-year death (n)** | **Median** | **5-year % OS** | **p**  **(Log-rank)** |
| **Amplification Peak 45** | 11p15.1 |  |  |  |  | 0.039 |
|  | No | 42 | 18 | 60 | 57.1 |  |
|  | Yes | 16 | 11 | 19 | 31.3 |  |
| **Amplification Peak 68** | 19p13.2 |  |  |  |  | 0.004 |
|  | No | 32 | 11 | 166 | 65.6 |  |
|  | Yes | 26 | 17 | 13 | 34.6 |  |
| **Amplification Peak 69** | 19q13.2 |  |  |  |  | 0.001 |
|  | No | 32 | 10 | 166 | 68.8 |  |
|  | Yes | 26 | 19 | 13 | 26.9 |  |
| **Amplification Peak 24** | 6p21.1 |  |  |  |  | 0.013 |
|  | No | 28 | 9 | 166 | 67.9 |  |
|  | Yes | 30 | 20 | 15 | 33.3 |  |
| **Amplification Peak 76** | Xp21.1 |  |  |  |  | 0.005 |
|  | No | 27 | 8 | 166 | 70.4 |  |
|  | Yes | 31 | 21 | 15 | 32.3 |  |
| **Deletion Peak 11** | 3q22.3 |  |  |  |  | 0.04 |
|  | No | 39 | 16 | 60 | 59.0 |  |
|  | Yes | 19 | 13 | 15 | 31.6 |  |
| **Deletion Peak 12** | 3q26.32 |  |  |  |  | 0.023 |
|  | No | 36 | 14 | NR | 61.1 |  |
|  | Yes | 22 | 15 | 15 | 31.8 |  |
| **Deletion Peak 50** | 13q14.2 |  |  |  |  | 0.001 |
|  | No | 44 | 18 | 166 | 59.1 |  |
|  | Yes | 14 | 11 | 9 | 21.4 |  |
| **Deletion Peak 26** | 7p12.2 |  |  |  |  | 0.016 |
|  | No | 34 | 13 | 166 | 61.8 |  |
|  | Yes | 24 | 16 | 17 | 33.3 |  |
| **Broad Deletion** | 17p |  |  |  |  | 0.021 |
|  | No | 52 | 24 | 48 | 53.8 |  |
|  | Yes | 6 | 5 | 1 | 16.7 |  |
| **Adults with poor risk cytogenetic^2^ (n=50)** | | | | | | |
| **Unique name** | **Cytoband** | **Patients (n)** | **5-year death (n)** | **Median** | **5-year % OS** | **p**  **(Log-rank)** |
| **Amplification Peak 20** | 5q31.1 |  |  |  |  | 0.019 |
|  | No | 28 | 17 | 20 | 39.3 |  |
|  | Yes | 14 | 12 | 5 | 14.3 |  |
| **Amplification Peak 41** | 10p15.3 |  |  |  |  | 0.01 |
|  | No | 34 | 21 | 17 | 38.2 |  |
|  | Yes | 8 | 8 | 5 | 0 |  |
| **Deletion Peak 6** | 1q22 |  |  |  |  | 0.005 |
|  | No | 32 | 19 | 15 | 40.6 |  |
|  | Yes | 10 | 10 | 1 | 0 |  |
| **Deletion Peak 11** | 3q22.3 |  |  |  |  | 0.018 |
|  | No | 31 | 18 | 15 | 41.9 |  |
|  | Yes | 11 | 11 | 9 | 0 |  |
| **Deletion Peak 12** | 3q26.32 |  |  |  |  | 0.028 |
|  | No | 33 | 21 | 14 | 36.4 |  |
|  | Yes | 9 | 8 | 3 | 11.1 |  |
| **Deletion Peak 44** | 11q23.3 |  |  |  |  | 0.04 |
|  | No | 34 | 21 | 14 | 38.2 |  |
|  | Yes | 8 | 8 | 3 | 0 |  |
| **Deletion Peak 60** | 16q22.1 |  |  |  |  | 0.017 |
|  | No | 25 | 14 | 17 | 44 |  |
|  | Yes | 17 | 15 | 9 | 11.8 |  |
|  | | | | | | |

1 Includes patients without t(9;22), t(v;11q23) and hypodiploidy.

2 Includes patients with t(9;22), t(v;11q23) and hypodiploidy.

**Table K. CNAs associated with shorter EFS in children with B-ALL**

| **CNAs associated with shorter EFS in the whole cohort of children with B-ALL (n=115)*** | | | | | | |
| --- | --- | --- | --- | --- | --- | --- |
| **Unique name** | **Cytoband** | **Patients (n)** | **5-year death (n)** | **Median** | **5-year % OS** | **p**  **(Log-rank)** |
| **Deletion Peak 2** | 1p12 |  |  |  |  | 0.027 |
|  | No | 82 | 15 | NR | 81.7 |  |
|  | Yes | 21 | 9 | 62 | 57.1 |  |
| **Deletion Peak 17** | 7p12.2 |  |  |  |  | 0.031 |
|  | No | 85 | 16 | NR | 81.2 |  |
|  | Yes | 18 | 8 | 62 | 55.6 |  |
| *There were no associations between CNAs and OS in children with B-ALL. **Abbreviation**: NR, not reached.  **Table L. CNAs associated with shorter OS in children with T-ALL**   \| **CNAs associated with shorter OS in the whole cohort of children with T-ALL (n=27)*** \| \| \| \| \| \| \| \| --- \| --- \| --- \| --- \| --- \| --- \| --- \| \| **Unique name** \| **Cytoband** \| **Patients (n)** \| **5-year death (n)** \| **Median** \| **5-year % OS** \| **p**  **(Log-rank)** \| \| Amplification Peak 11 \| Xq28 \|  \|  \|  \|  \| 0.008 \| \| No \| 18 \| 2 \| NR \| 88.9 \| \| Yes \| 7 \| 4 \| 25 \| 42.9 \| \| *There were no associations between CNAs and EFS in children with T-ALL. **Abbreviation**: NR, not reached. \| \| \| \| \| \| \| | | | | | | |

**Table M. CNAs associated with shorter OS and EFS in adults with B-ALL**

| **CNAs associated with shorter OS in the whole cohort of adults with B-ALL (n=100)** | | | | | | |
| --- | --- | --- | --- | --- | --- | --- |
| **Unique name** | **Cytoband** | **Patients (n)** | **5-year death (n)** | **Median** | **5-year % OS** | **p**  **(Log-rank)** |
| Amplification Peak 6 | 2p11.2 |  |  |  |  | 0.023 |
|  | No | 66 | 36 | 22 | 45.5 |  |
|  | Yes | 17 | 14 | 10 | 17.6 |  |
| Amplification Peak 9 | 3p25.3 |  |  |  |  | 0.018 |
|  | No | 71 | 39 | 22 | 45.1 |  |
|  | Yes | 12 | 11 | 7 | 8.3 |  |
| Amplification Peak 15 | 6p21.1  No  Yes | 49  34 | 22  28 | 46  7 | 55.1  17.6 | <0.0001 |
| Amplification Peak 16 | 6q27  No  Yes | 67  16 | 37  13 | 22  4 | 44.8  18.8 | 0.027 |
| Amplification Peak 29 | 10q22.3  No  Yes | 54  29 | 27  23 | 29  9 | 50.0  20.7 | 0.007 |
| Amplification Peak 31 | 11q13.1  No  Yes | 67  16 | 36  14 | 22  10 | 46.3  12.5 | 0.027 |
| Amplification Peak 44 | 18q21  No  Yes | 59  24 | 29  21 | 46  7 | 50.8  12.5 | <0.0001 |
| Amplification Peak 54 | Xq28  No  Yes | 41  42 | 20  30 | 46  11 | 51.2  28.6 | 0.024 |
| Deletion Peak 10 | 4p14  No  Yes | 71  12 | 39  11 | 22  3 | 45.1  8.3 | 0.002 |
| Deletion Peak 21 | 8q21.13  No  Yes | 74  9 | 42  8 | 20  1 | 43.2  11.1 | 0.012 |
| Deletion Peak 27 | 13q14.2  No  Yes | 72  11 | 40  10 | 22  4 | 44.4  9.1 | 0.009 |
| Deletion Peak 30 | 15q13.2  No  Yes | 64  19 | 34  17 | 29  6 | 48.4  10.5 | 0.003 |
| **CNAs associated with shorter EFS in the whole cohort of adults with B-ALL (n=100)** | | | | | | |
| **Unique name** | **Cytoband** | **Patients (n)** | **5-year death (n)** | **Median** | **5-year % OS** | **p**  **(Log-rank)** |
| Amplification Peak 1 | 1p36.13 |  |  |  |  | 0.034 |
|  | No | 56 | 35 | 14 | 37.5 |  |
|  | Yes | 22 | 18 | 3 | 18.2 |  |
| Amplification Peak 9 | 3p25.3 |  |  |  |  | 0.018 |
|  | No | 65 | 41 | 12 | 36.9 |  |
|  | Yes | 13 | 12 | 1 | 7.7 |  |
| Amplification Peak 10 | 4p16.1 |  |  |  |  | 0.030 |
|  | No | 62 | 39 | 14 | 37.1 |  |
|  | Yes | 16 | 14 | 3 | 12.5 |  |
| Amplification Peak 15 | 6p21.1 |  |  |  |  | <0.0001 |
|  | No | 44 | 22 | 38 | 50.0 |  |
|  | Yes | 34 | 31 | 4 | 8.8 |  |
| Amplification Peak 29 | 10q22.3 |  |  |  |  | 0.019 |
|  | No | 48 | 28 | 17 | 41.7 |  |
|  | Yes | 30 | 25 | 5 | 16.7 |  |
| Amplification Peak 31 | 11q13.1  No  Yes | 61  17 | 37  16 | 14  5 | 39.3  5.9 | 0.011 |
| Amplification Peak 34 | 12q13.13  No  Yes | 53  25 | 32  21 | 14  3 | 39.6  16.0 | 0.019 |
| Amplification Peak 44 | 18q21.1  No  Yes | 54  24 | 31  22 | 20  3 | 42.6  8.3 | <0.0001 |
| Amplification Peak 47 | 21q22.3  No  Yes | 45  33 | 25  28 | 17  5 | 44.4  15.2 | 0.029 |
| Amplification Peak 54 | Xq28  No  Yes | 37  41 | 20  33 | 32  5 | 45.9  19.5 | 0.008 |
| Deletion Peak 3 | 1p12  No  Yes | 59  19 | 37  16 | 17  5 | 37.3  15.8 | 0.034 |
| Deletion Peak 10 | 4p14  No  Yes | 65  13 | 41  12 | 14  1 | 36.9  7.7 | 0.002 |
| Deletion Peak 21 | 8q21.13  No  Yes | 68  10 | 44  9 | 12  1 | 35.3  10.0 | 0.023 |
| Deletion Peak 30 | 15q13.2  No  Yes | 57  21 | 34  19 | 14  4 | 40.4  9.5 | 0.009 |
| Deletion Peak 33 | 16q22.1  No  Yes | 62  16 | 39  14 | 14  1 | 37.1  12.5 | 0.009 |
|  | | | | | | |

**Supplementary Information References**

1. van Dongen JJ, Macintyre EA, Gabert JA, Delabesse E, Rossi V, Saglio G, et al. Standardized RT-PCR analysis of fusion gene transcripts from chromosome aberrations in acute leukemia for detection of minimal residual disease. Report of the BIOMED-1 Concerted Action: investigation of minimal residual disease in acute leukemia. Leukemia. 1999;13(12):1901-28. Epub 1999/12/22. PubMed PMID: 10602411.

2. Lai W, Choudhary V, Park PJ. CGHweb: a tool for comparing DNA copy number segmentations from multiple algorithms. Bioinformatics. 2008;24(7):1014-5. doi: 10.1093/bioinformatics/btn067. PubMed PMID: 18296463; PubMed Central PMCID: PMC2516369.

3. Beroukhim R, Getz G, Nghiemphu L, Barretina J, Hsueh T, Linhart D, et al. Assessing the significance of chromosomal aberrations in cancer: methodology and application to glioma. Proc Natl Acad Sci U S A. 2007;104(50):20007-12. doi: 10.1073/pnas.0710052104. PubMed PMID: 18077431; PubMed Central PMCID: PMC2148413.

4. Mullighan CG, Goorha S, Radtke I, Miller CB, Coustan-Smith E, Dalton JD, et al. Genome-wide analysis of genetic alterations in acute lymphoblastic leukaemia. Nature. 2007;446(7137):758-64. Epub 2007/03/09. doi: nature05690 [pii] 10.1038/nature05690. PubMed PMID: 17344859.

5. Kawamata N, Ogawa S, Zimmermann M, Kato M, Sanada M, Hemminki K, et al. Molecular allelokaryotyping of pediatric acute lymphoblastic leukemias by high-resolution single nucleotide polymorphism oligonucleotide genomic microarray. Blood. 2008;111(2):776-84. doi: 10.1182/blood-2007-05-088310. PubMed PMID: 17890455; PubMed Central PMCID: PMC2200831.

6. Kuiper RP, Schoenmakers EF, van Reijmersdal SV, Hehir-Kwa JY, van Kessel AG, van Leeuwen FN, et al. High-resolution genomic profiling of childhood ALL reveals novel recurrent genetic lesions affecting pathways involved in lymphocyte differentiation and cell cycle progression. Leukemia. 2007;21(6):1258-66. Epub 2007/04/20. doi: 2404691 [pii]10.1038/sj.leu.2404691. PubMed PMID: 17443227.

7. Strefford JC, Worley H, Barber K, Wright S, Stewart AR, Robinson HM, et al. Genome complexity in acute lymphoblastic leukemia is revealed by array-based comparative genomic hybridization. Oncogene. 2007;26(29):4306-18. Epub 2007/01/24. doi: 1210190 [pii]10.1038/sj.onc.1210190. PubMed PMID: 17237825.

8. Simons A, Stevens-Kroef M, El Idrissi-Zaynoun N, van Gessel S, Weghuis DO, van den Berg E, et al. Microarray-based genomic profiling as a diagnostic tool in acute lymphoblastic leukemia. Genes Chromosomes Cancer. 2011;50(12):969-81. doi: 10.1002/gcc.20919. PubMed PMID: 21882283.

9. Rabin KR, Man TK, Yu A, Folsom MR, Zhao YJ, Rao PH, et al. Clinical utility of array comparative genomic hybridization for detection of chromosomal abnormalities in pediatric acute lymphoblastic leukemia. Pediatr Blood Cancer. 2008;51(2):171-7. Epub 2008/02/07. doi: 10.1002/pbc.21488. PubMed PMID: 18253961.

10. Okamoto R, Ogawa S, Nowak D, Kawamata N, Akagi T, Kato M, et al. Genomic profiling of adult acute lymphoblastic leukemia by single nucleotide polymorphism oligonucleotide microarray and comparison to pediatric acute lymphoblastic leukemia. Haematologica. 2010;95(9):1481-8. Epub 2010/05/04. doi: haematol.2009.011114 [pii]10.3324/haematol.2009.011114. PubMed PMID: 20435627; PubMed Central PMCID: PMC2930948.

11. Safavi S, Hansson M, Karlsson K, Biloglav A, Johansson B, Paulsson K. Novel gene targets detected by genomic profiling in a consecutive series of 126 adults with acute lymphoblastic leukemia. Haematologica. 2015;100(1):55-61. doi: 10.3324/haematol.2014.112912. PubMed PMID: 25261097; PubMed Central PMCID: PMC4281313.

12. Paulsson K, Cazier JB, Macdougall F, Stevens J, Stasevich I, Vrcelj N, et al. Microdeletions are a general feature of adult and adolescent acute lymphoblastic leukemia: Unexpected similarities with pediatric disease. Proc Natl Acad Sci U S A. 2008;105(18):6708-13. Epub 2008/05/07. doi: 0800408105 [pii]10.1073/pnas.0800408105. PubMed PMID: 18458336; PubMed Central PMCID: PMC2373322.

13. Muhlbacher V, Haferlach T, Kern W, Zenger M, Schnittger S, Haferlach C. Array-based comparative genomic hybridization detects copy number variations with prognostic relevance in 80% of ALL with normal karyotype or failed chromosome analysis. Leukemia. 2015. doi: 10.1038/leu.2015.276. PubMed PMID: 26449660.

14. Yasar D, Karadogan I, Alanoglu G, Akkaya B, Luleci G, Salim O, et al. Array comparative genomic hybridization analysis of adult acute leukemia patients. Cancer Genet Cytogenet. 2010;197(2):122-9. Epub 2010/03/03. doi: S0165-4608(09)00670-0 [pii]10.1016/j.cancergencyto.2009.11.018. PubMed PMID: 20193845.

15. Usvasalo A, Raty R, Harila-Saari A, Koistinen P, Savolainen ER, Vettenranta K, et al. Acute lymphoblastic leukemias with normal karyotypes are not without genomic aberrations. Cancer Genet Cytogenet. 2009;192(1):10-7. Epub 2009/06/02. doi: S0165-4608(09)00133-2 [pii]10.1016/j.cancergencyto.2009.02.015. PubMed PMID: 19480931.

16. Steinemann D, Cario G, Stanulla M, Karawajew L, Tauscher M, Weigmann A, et al. Copy number alterations in childhood acute lymphoblastic leukemia and their association with minimal residual disease. Genes Chromosomes Cancer. 2008;47(6):471-80. Epub 2008/03/04. doi: 10.1002/gcc.20557. PubMed PMID: 18311775.

17. Olsson L, Castor A, Behrendtz M, Biloglav A, Forestier E, Paulsson K, et al. Deletions of IKZF1 and SPRED1 are associated with poor prognosis in a population-based series of pediatric B-cell precursor acute lymphoblastic leukemia diagnosed between 1992 and 2011. Leukemia. 2014;28(2):302-10. doi: 10.1038/leu.2013.206. PubMed PMID: 23823658.

18. Baughn LB, Biegel JA, South ST, Smolarek TA, Volkert S, Carroll AJ, et al. Integration of cytogenomic data for furthering the characterization of pediatric B-cell acute lymphoblastic leukemia: a multi-institution, multi-platform microarray study. Cancer genetics. 2015;208(1-2):1-18. doi: 10.1016/j.cancergen.2014.11.003. PubMed PMID: 25678190.

19. Bungaro S, Dell'Orto MC, Zangrando A, Basso D, Gorletta T, Lo Nigro L, et al. Integration of genomic and gene expression data of childhood ALL without known aberrations identifies subgroups with specific genetic hallmarks. Genes Chromosomes Cancer. 2009;48(1):22-38. Epub 2008/09/23. doi: 10.1002/gcc.20616. PubMed PMID: 18803328.
