# Supplementary figures and images for: Genome-Wide DNA Copy Number Analysis of Acute Lymphoblastic Leukemia Identifies New Genetic Markers Associated with Clinical Outcome
Source: PLoS One. 2016 Feb 12;11(2):e0148972. doi: 10.1371/journal.pone.0148972 (PMC4752220; doi:10.1371/journal.pone.0148972)

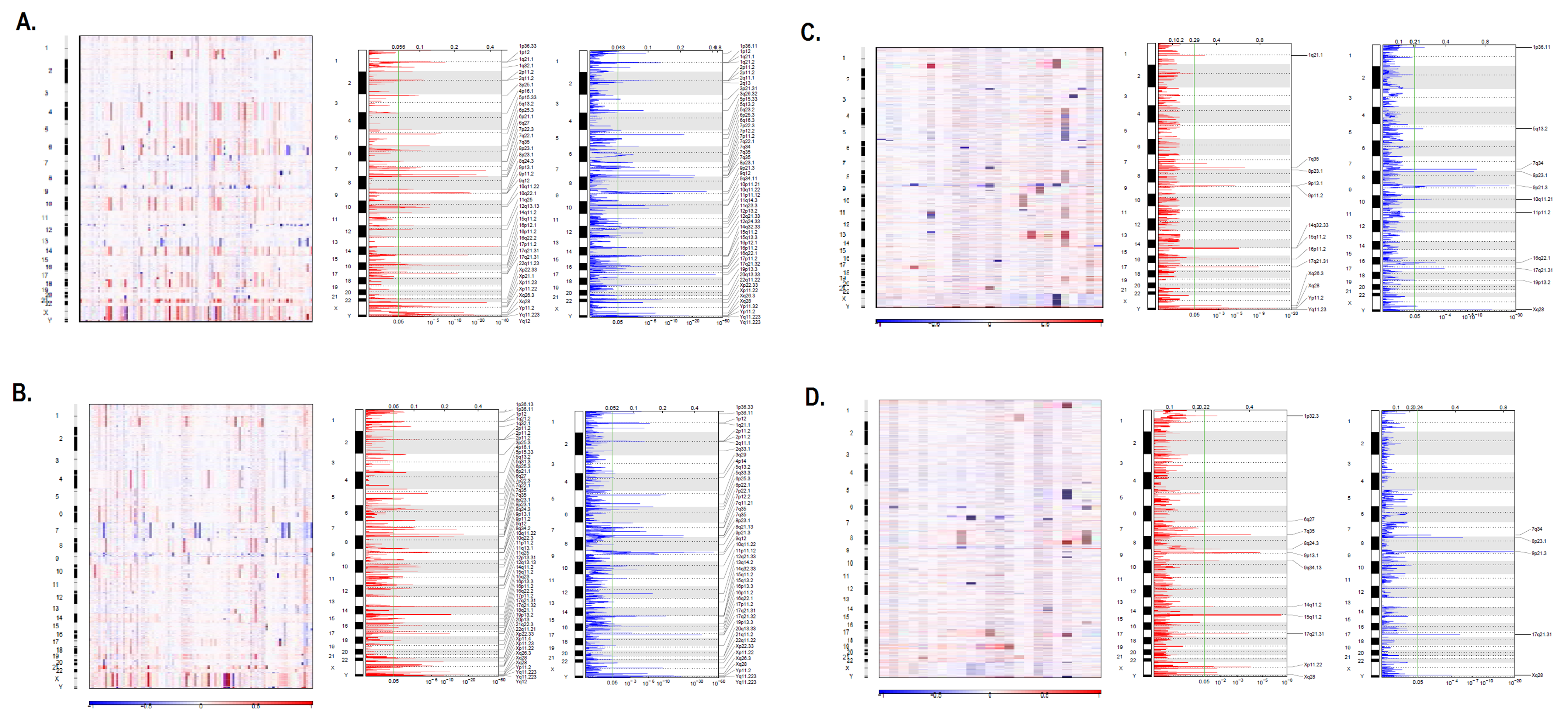

Supplement: S1 Fig — (A). Log2-ratio copy number heatmap of array-based comparative genomic hybridization (aCGH) data in child B-ALL (n = 115)/adult B-ALL (n = 100) and child T-ALL (n = 27)/adult T-ALL (n = 23) (gains: red; losses: blue). (B). Regions of significant recurrent amplification and deletion in child B-ALL (n = 115)/adult B-ALL (n = 100) and child T-ALL (n = 27)/adult T-ALL (n = 23) patients (q<0.05). (TIF) [file pone.0148972.s002.tif]

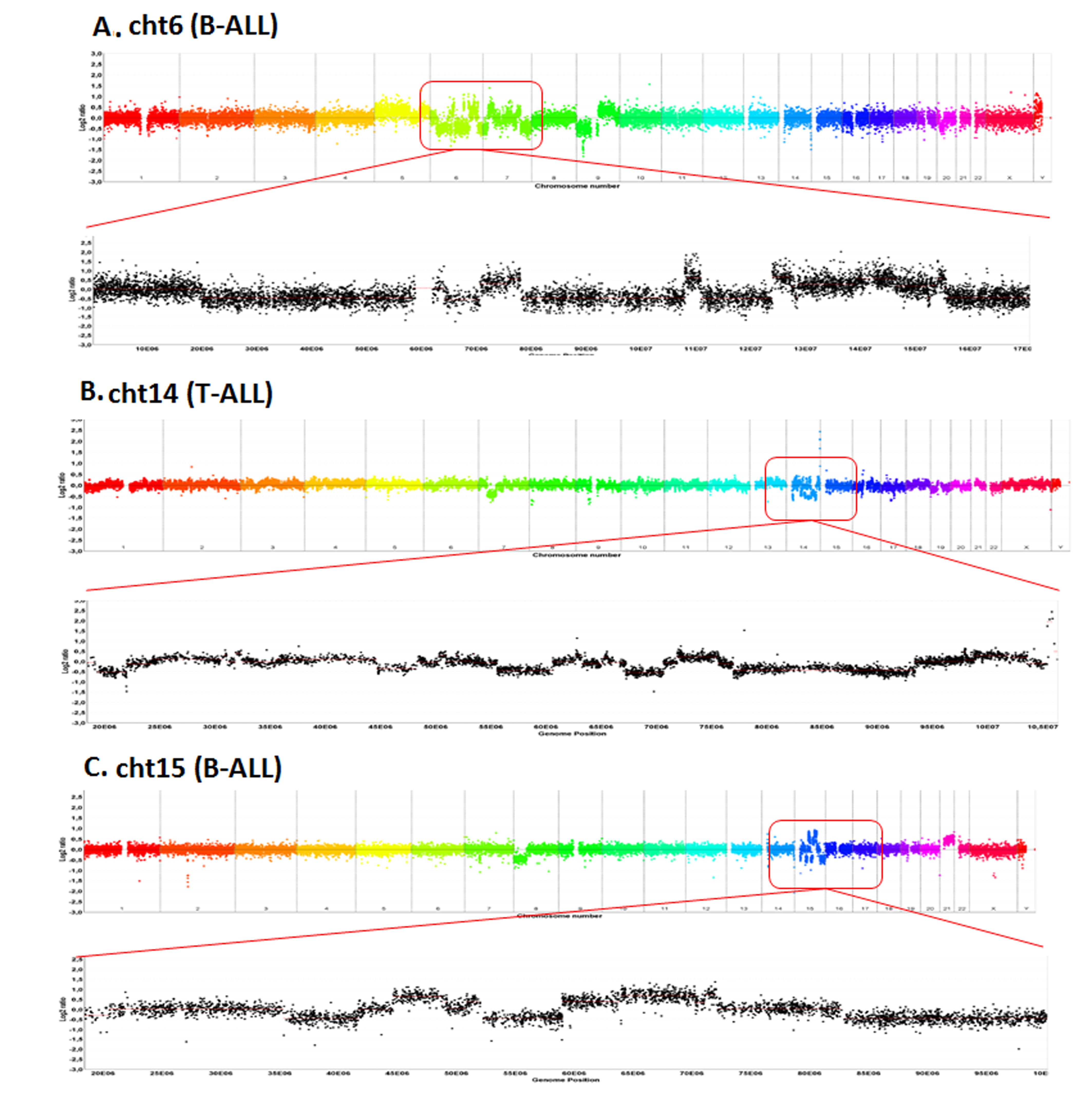

Supplement: S2 Fig — As is typical of chromothripsis (cth), copy-number profiles showed multiple oscillations between two DNA copy-number states. (A) B-ALL patient with cth on chromosome 6. (B) T-ALL patient with cth on chromosomes 14. (C) B-ALL patient with cth on chromosome 15. (TIF) [file pone.0148972.s003.tif]
